# Nifuroxazide and 4-Hydroxybenzhydrazone Derivatives as New Antiparasitic (*Trypanosoma cruzi* and *Leishmania mexicana*) and Anti-*Mycobacterium tuberculosis* Agents

**DOI:** 10.3390/pharmaceutics17050621

**Published:** 2025-05-07

**Authors:** Timoteo Delgado-Maldonado, Diana V. Navarrete-Carriola, Lenci K. Vázquez-Jiménez, Alma D. Paz-González, Baojie Wan, Scott Franzblau, Othman Mueen Mohammed, Lorena Rodríguez-Páez, Charmina Aguirre-Alvarado, Verónica Alcántara-Farfán, Joaquín Cordero-Martínez, Debasish Bandyopadhyay, Adriana Moreno-Rodríguez, Gildardo Rivera

**Affiliations:** 1 Laboratorio de Biotecnología Farmacéutica, Centro de Biotecnología Genómica, Instituto Politécnico Nacional, Reynosa 88710, Mexico; titi_999@live.com (T.D.-M.); civky82@gmail.com (D.V.N.-C.); lenka.18@hotmail.com (L.K.V.-J.); apazg@ipn.mx (A.D.P.-G.); 2 Institute for Tuberculosis Research, College of Pharmacy, University of Illinois at Chicago, 833 S. Wood St., Chicago, IL 60612, USA; baojie@uic.edu (B.W.); sgf@uic.edu (S.F.); 3 Department of Medical Laboratories Techniques, College of Health and Medical Technology, University of Al Maarif, Al Anbar 31001, Iraq; zznnmmslds@gmail.com; 4 Departamento de Bioquímica, Escuela Nacional de Ciencias Biológicas, Instituto Politécnico Nacional, Ciudad de México 11340, Mexico; lrodrig@ipn.mx (L.R.-P.); caguirrea@ipn.mx (C.A.-A.); valcantaraf@ipn.mx (V.A.-F.); jcorderom@ipn.mx (J.C.-M.); 5 School of Integrative Biological and Chemical Sciences (SIBCS), University of Texas Rio Grande Valley, Edinburg, TX 78539, USA; debasish.bandyopadhyay@utrgv.edu; 6 School of Earth, Environmental, and Marine Sciences (SEEMS), University of Texas Rio Grande Valley, Edinburg, TX 78539, USA; 7 Laboratorio de Estudios Epidemiológicos, Clínicos, Diseños Experimentales e Investigación, Facultad de Ciencias Químicas, Universidad Autónoma “Benito Juárez” de Oaxaca, Avenida Universidad S/N, Oaxaca 68120, Mexico

**Keywords:** hydrazone moiety, nifuroxazide, cysteine protease inhibitors, *Leishmania mexicana*, *Trypanosoma cruzi*, anti-*Mycobacterium tuberculosis*

## Abstract

**Background/Objectives:** Nifuroxazide (Nfz) is a drug that has been used as a scaffold for designing antimicrobial and antiparasitic agents. This study aimed to synthesize and evaluate in vitro of Nfz and twenty-five 4-hydroxybenzhydrazone derivatives as potential anti-*Trypanosoma cruzi*, anti-*Leishmania mexicana*, and anti-*Mycobacterium tuberculosis* agents. **Methods:** The compounds were synthesized by condensing 4-hydroxybenzhydrazide with appropriate aldehydes in acidic conditions and structurally confirmed by spectroscopic techniques. All compounds were evaluated in vitro against *T. cruzi* strains (NINOA and A1), *L. mexicana* (M379 and FCQEPS strains), and *M. tuberculosis* (H37Rv strain), followed by enzymatic assays against *T. cruzi* cysteine proteases. **Results:** Compound **Nfz-24** (IC_50_ = 6.8 μM) had better trypanocidal activity than the reference drugs benznidazole (IC_50_ > 30 μM) and nifurtimox (IC_50_ > 7 μM) against the NINOA strain, and **Nfz-8** (IC_50_ = 7.2 μM) was the compound most active against the A1 strain with a high inhibition of *T. cruzi* cysteine proteases (IC_50_ = 4.6 μM) and low cytotoxic effects (CC_50_ >100 μM). On the other hand, compound **Nfz-5** (IC_50_ = 5.2 μM) had a 25-fold better leishmanicidal effect than glucantime (IC_50_ > 125 μM) against the *L. mexicana* M379 strain, and compound **Nfz-13** had the best leishmanicidal effects (IC_50_ = 10.2 μM) against the FCQEPS strain. Finally, **Nfz**, **Nfz-1**, and **Nfz-2** had minimum inhibitory concentration (MIC) values of 12.3, 5.1, and 18.8 μg/mL against *M. tuberculosis*, respectively. **Conclusions:** In summary, these results suggest that the compounds **Nfz-1**, **Nfz-2**, **Nfz-5**, **Nfz-8**, **Nfz-10**, **Nfz-15**, **Nfz-24**, and **Nfz-25** are candidates for further studies to develop new and more potent anti-*T. cruzi*, anti-leishmaniasis, and anti-*M. tuberculosis* agents.

## 1. Introduction

**Figure 1:**
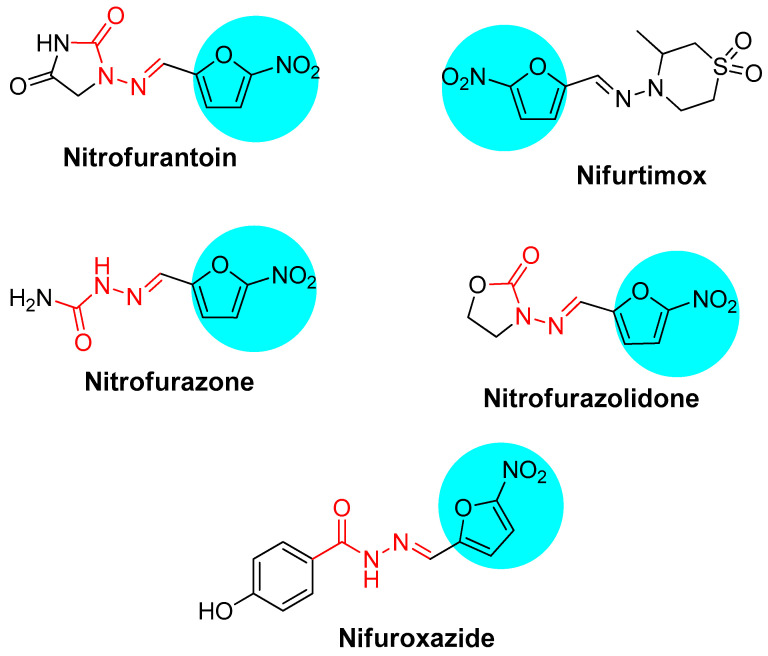
Chemical structures of some approved 5-nitrofuran (blue circle) drugs containing the *N*-acylhydrazone framework (red).

Nitrofurans are a notable class of heterocycles renowned for their antimicrobial properties. They feature a nitro group attached to a furan ring, which enables them to effectively combat bacterial and parasitic infections by generating free radicals that damage microbial DNA [1]. Nitrofuran drugs (Figure 1) are widely used for treating bacterial or protozoa infections [2]. However, despite their efficacy, their use is associated with potential side effects, such as gastrointestinal discomfort and concerns about toxicity and microbial resistance [3]. Therefore, the current research is focused on enhancing their therapeutic efficacy while minimizing adverse effects and resistance issues [4].

**Figure 2:**
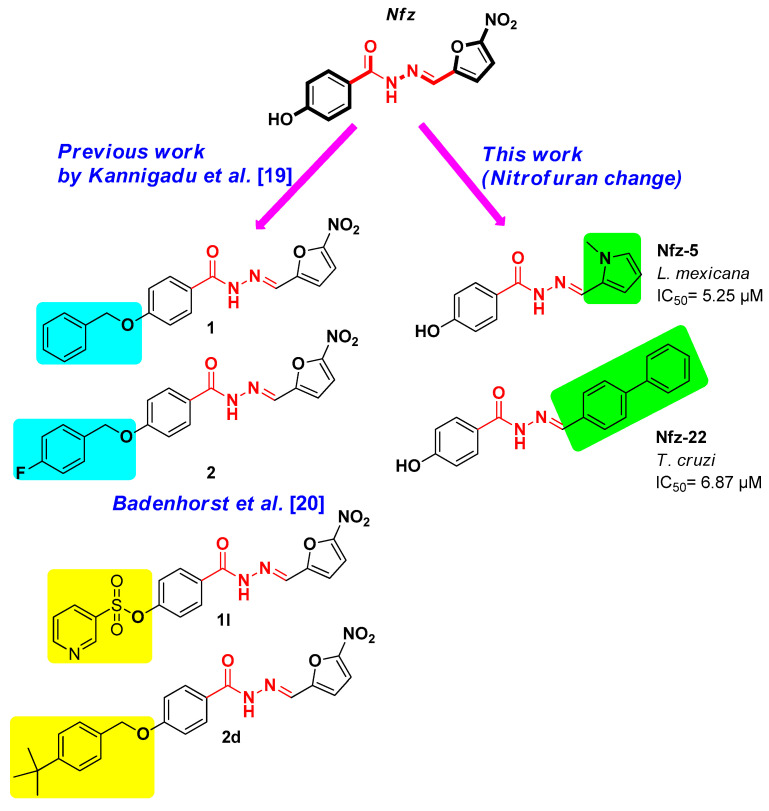
Structural modification of nifuroxazide to obtain new derivatives with anti-*T. cruzi*, anti-*L. mexicana*, and anti-*Mycobacterium tuberculosis* activity. The pink arrows indicate the final compounds reported by Kannigadu et al. [19], Badenhorst et al. [20], and those obtained in this investigation. The blue square highlights the chemical modification to Nfz performed by Kannigadu et al., and the yellow square indicates the substituents incorporated into Nfz reported by Badenhorst et al. The green box shows the substituents incorporated into the 4-hydroxybenzhydrazide fragment in this work.

Nifuroxazide (Nfz) (Figure 1) is a nitrofuran derivative that presents the hydrazone framework, which is described as a pharmacophore with a broad spectrum of biological activity, including anti-*Mycobacterium tuberculosis* [5,6,7,8,9,10,11,12]. Nfz is used for the treatment of acute infectious diarrhea and colitis [13], although it also exhibits antibacterial [14] and anticancer activities, among others [8,15,16]. Recent studies have demonstrated that Nfz has antiparasitic effects, including trypanocidal and leishmanicidal activities [17,18]. Therefore, molecular modifications maintaining the nitrofuran moiety to improve biological activity have been carried out. For example, Kannigadu et al., designed and synthesized the *O*-alkylated and *N*-alkylated analogs of *O*-benzylated nifuroxazide as leishmanicidal agents (Figure 2). Compounds 1 (IC_50_ = 0.25 to 1.22 µM) and 2 (IC_50_ = 0.23 to 0.86 µM) were the most effective against promastigotes of the *Leishmania donovani* 1S, *L. donovani* 9515, and *Leishmania major* IR-173 strains with a high selectivity index (SI) [19]. Additionally, Badenhorst et al. reported the synthesis of a series of Nfz-derived compounds incorporating a sulfonate and *O*-benzyl substituted moiety. Their results showed that compound 1l (the pyridine ring bearing sulfonate, Figure 2) had a potent effect against the intracellular amastigotes of the *L. donovani* 9515 strain and *L. major* IR-173 strain with IC_50_ values of 3.26 µM and 1.26 µM, respectively. Meanwhile, compound 2d (IC_50_ = 0.19 µM, Figure 2) had a high effect against amastigotes of the *L. major* IR-173 strain [20]. However, nitrofuran moiety has been related to cytotoxic effects in normal cell lines [21]. Therefore, in this study, Nfz and a series of 4-hydroxybenzhydrazone derivatives were proposed, changing the nitrofuran moiety for bioisosteric and new aromatic groups (Figure 2) that could improve or maintain the biological effects against the protozoa *Trypanosoma cruzi* and *Leishmania mexicana*, and their potential anti-*M. tuberculosis* activities were evaluated in an in vitro model. Additionally, these structural modifications could reduce the cytotoxic effects in normal cell lines. Finally, the mechanism of action as potential cruzain inhibitors was explored.

## 2. Materials and Methods

### 2.1. Chemistry

All reagents were purchased from Sigma Aldrich Chemical Co. (Toluca, Mexico) and used without purifying them further. Thin-layer chromatography (TLC) aluminum oxide matrix Z234214-1PAK sheets from Sigma Aldrich^®^ were used to monitor the reaction. Solvents (ethanol) employed were of analytical grade. Infrared (IR) spectra were recorded on an FT-IR Bruker Tensor 27 spectrophotometer coupled to a Bruker (Bruker Corporation, Billerica, MA, USA) platinum ATR cell. Mass spectrometry ESI–MS analyses were conducted on an Ultra Performance Liquid Chromatography–Mass Spectrometer (UPLC-MS) waters. Nuclear magnetic resonance (NMR) spectra were measured on a Bruker Ultrashield-500 spectrometer (Bruker Corporation, Billerica, MA, USA), using solvents (CDCl_3_-*d_6_* and DMSO-*d_6_*) and, in some cases, TMS as a reference. Chemical shifts are presented on the δ scale (ppm). Multiplicities are indicated as s (singlet), d (doublet), t (triplet), m (multiplet), or br (broad).

The general procedure for the preparation of (*E*)-*N’*-benzylidene-4-hydroxybenzohydrazide derivatives (Nfz series) was as follows: 4-hydroxybenzhydrazide (1 mmol) and correspondent aldehyde (1 mmol) were added at room temperature in 50 mL round-bottomed flask containing 20 mL ethanol in constant agitation. Then, acetic acid drops were used as a catalyst. The mixture was refluxed for 3–6 h, and the progress of the reaction was monitored by TLC. The reaction product (precipitate) was isolated by filtration and three successive washes using heat *n*-hexane (3 × 20 mL). The final product was obtained without further purification.

### 2.2. Biological Evaluations

#### 2.2.1. Trypanocidal Activity

Two *T. cruzi* isolates were used to evaluate the trypanocidal activity: in 1986, NINOA (MHOM/MX/1994/NINOA) strain via the xenodiagnosis from an acute case of trypanosomiasis in Mexico and strain A1, obtained in 2018 from *Triatoma dimidiata* in Tehuantepec, Mexico. To maintain the strains, the liver infusion tryptose (LIT) medium was supplemented with 10% fetal bovine serum (FBS) and 0.1% penicillin–streptomycin. To preserve them, parasites were transferred into a new culture medium each week. The activity of compounds, including the drugs nifurtimox (Nfx) and benznidazole (Bzn), was assessed against *T. cruzi* epimastigotes. A stock solution of all compounds was prepared at 10 mg/mL, using dimethyl sulfoxide (DMSO) as a diluent. Serial dilutions were performed using LIT medium until concentrations of 100 to 0.46 µg/mL of each compound were obtained. *T. cruzi* epimastigotes (1 × 10^6^/well) were cultured in 96-well microtiter plates and incubated at 28 °C for 48 h with the reference drugs and samples at different concentrations (0.78 to 200 µM) in a final volume of 200 µL. DMSO was included as a negative control. Next, 20 µL of resazurin solution (2.5 mM) was added to each well and incubated for 3 h. All assays were performed in triplicate. The half-maximum inhibitory concentration (IC_50_) values were calculated by probit analysis.

#### 2.2.2. Leishmanicidal Activity

For leishmanicidal assays, the reference strain (MNYC/BZ/62/M379) and the isolate (MHOM/MX/2017/UABJO17FCQEPS) of *L. mexicana* were used. Promastigotes were cultured in RPMI 1640 medium supplemented with 10% FBS, 100 U/mL penicillin-100 mg/mL streptomycin, and glutamine (2 mM). 100 µL parasites (5 × 10^5^/mL) were added in a 96-well plate, and 100 µL of appropriate concentrations (0.78 to 200 µM) of each sample dissolved in DMSO and incubated for 48 h at 26 °C. Wells containing promastigotes with DMSO 0.2% were used as a negative control, and the reference drug glucantime was included as the positive control. The 3-(4,5-Dimethylthiazol-2-yl)-2,5-diphenyltetrazolium bromide (MTT) was used to measure metabolic activity. The IC_50_ values were calculated with the probit statistical tool. All samples were evaluated in triplicate in three independent experiments.

#### 2.2.3. Anti-*Mycobacterium* Activity

The reference strain H37Rv (ATCC 27294) of *M. tuberculosis* was utilized to assess anti-*M. tuberculosis* activity using the microplate Alamar Blue Assay (MABA), as previously reported [22,23]. Samples underwent evaluation in triplicate independent experiments. Isoniazid, rifampicin, moxifloxacin, and linezolid were included as reference drugs, and the minimum drug concentration that achieved 90% inhibition was recorded as the minimum inhibitory concentration (MIC).

#### 2.2.4. Cytotoxicity

Cytotoxicity was assessed on J774.2 mouse macrophage cells (ATCC^®^ TIB-67, ATCC, Manassas, VA, USA). Cells were cultured in RPMI medium supplemented with 10% SFB, 100 U µg/mL penicillin-100 mg/mL streptomycin, and glutamine (2 mM) at 37 °C and in a 5% CO_2_ atmosphere. A known concentration of cells (1 × 10^6^/mL) was incubated with the compounds at different concentrations (0.78 to 200 µM) for 48 h at 37 °C in a CO_2_ atmosphere (5%). Wells containing cells treated with 0.2% DMSO were used as a negative control. MTT was employed to evaluate the metabolic activity. Next, the cell viability was calculated (%), and the cytotoxic concentration (CC_50_) was determined with probit analysis. All samples were evaluated in triplicate in three independent experiments. The selectivity index (SI) was calculated as the quotient of CC_50_/IC_50_.

#### 2.2.5. *T. cruzi* Cysteine Proteases Inhibition Assay

*T. cruzi* cruzain has been validated as a drug target and *N*-acyl hydrazone derivatives as inhibitors; therefore, we decided to investigate the ability of **Nfz** and the 4-hydroxybenzhydrazone derivatives to inhibit cysteine proteases like *T. cruzi* cruzain. Briefly, proteins from *T. cruzi* epimastigotes of the NINOA strain were obtained and quantified using the Bradford method [24]. The activity assay was performed using 5 µM of the fluorogenic substrate Z-Phe-Arg-MCA, 1 µg of protein extract, and different concentrations of the tested compounds **Nfz**, **Nfz-1**, **Nfz-4**, **Nfz-8**, and **Nfz-11** (0.01 to 1000 µg/mL, depending on their solubility). The buffer reaction solution contains 50 mM Na_2_HPO_4_, 100 mM NaCl, 5 mM EDTA, and 2.5 mM dithiothreitol (pH 6.5). A DMSO solution (2%) was included as negative control. Substrate hydrolysis was continuously measured for 1 h using a SpectraMax M5 spectrofluorometer (Molecular Devices) with excitation at 380 nm and emission at 440 nm at room temperature. Fluorescence values were normalized by setting the untreated control (no inhibitor) as 100%. IC_50_ values were calculated from semi-logarithmic concentration-response curves using a nonlinear regression approach implemented via the Dose-Response Analysis (DRA) package in R software (version 4.3.1), and results were expressed in µM [25,26].

## 3. Results

### 3.1. Synthesis

**Scheme 1:**
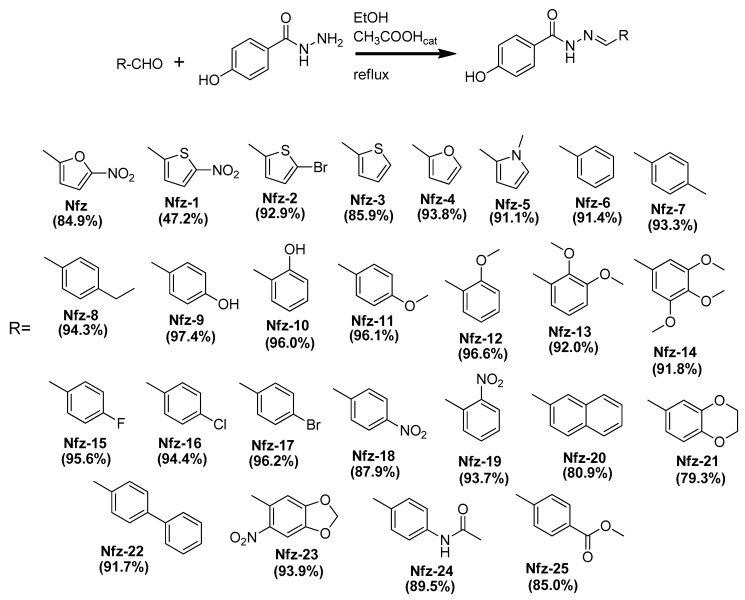
Synthetic route and percentage of yield of nifuroxazide and 4-hydroxybenzhydrazone derivatives obtained.

Nifuroxazide (**Nfz**) and twenty-five 4-hydroxybenzhydrazone derivatives were synthesized through a condensation reaction between 4-hydroxybenzhydrazide (1 mmol) and the corresponding aldehyde (1 mmol) in refluxing ethanol and in the presence of acetic acid as a catalyst (Scheme 1). In general, yields obtained were high (>80%), except for **Nfz-1** (<50%). All compounds were characterized by FT-IR, NMR, and UPLC-MS (see Supplementary Material). The ^1^H-NMR spectrum confirmed the structure of the compounds by the typical signal presence of the imine bond (N=CH, 8 ppm) and the N-H bond (12–11.5 ppm). In addition, their molecular mass was corroborated by the *m*/*z* spectrum, where the majority ion (M+H) for each of the synthesized molecules was observed. These results coincide with spectral data for some previously reported compounds [18,27].

### 3.2. Trypanocidal Activity

Table 1 shows the trypanocidal activity of **Nfz** and 4-hydroxybenzhydrazone derivatives against *T. cruzi* epimastigotes from two strains (A1 and NINOA). In general, the A1 strain had a greater sensitivity to **Nfz** and the 4-hydroxybenzhydrazone derivatives than the NINOA strain. **Nfz** was more active against the A1 strain (IC_50_ = 10.4 µM) than against the NINOA strain (IC_50_ = 36 µM). Compound **Nfz-1** exhibited superior anti-*T. cruzi* activity in the tested strains compared to previous reports against the Y strain [18]. The most potent compound against the A1 strain was **Nfz-8** (IC_50_ = 7.2 µM), a *para*-ethyl phenyl derivative. However, compound **Nfz-15** (IC_50_ = 8.7 µM) had comparable trypanocidal activity. Additionally, derivatives **Nfz-3** (IC_50_ = 19.9 µM) and **Nfz-11** (IC_50_ = 18.1 μM) had a similar effect to Nfx (IC_50_ = 19.3 μM). Meanwhile, **Nfz** and twelve 4-hydroxybenzhydrazone derivatives (**Nfz-1**, **Nfz-3**, **Nfz-4**, **Nfz-8, Nfz-9**, **Nfz-11**, **Nfz-13**, **Nfz-15**, **Nfz-17**, **Nfz-19**, **Nfz-22**, and **Nfz-25**) had better activity than reference drug Bzn (IC_50_ = 39 μM) against the A1 strain. Compounds **Nfz-2** (IC_50_ = 4.7 μM) and **Nfz-24** (IC_50_ = 6.8 μM) had an equal or similar trypanocidal effect against epimastigotes of the NINOA strain than Nfx (IC_50_ = 7 μM). Interestingly, compounds **Nfz**, **Nfz-1**, **Nfz-4**, **Nfz-8**, and **Nfz-11** had a good anti-*T. cruzi* activity against both strains. Therefore, these compounds were selected to investigate their potential mechanism of action as potential cruzain inhibitors.

### 3.3. Inhibition of T. cruzi Cysteine Protease Activity

To investigate the mechanism of action of **Nfz** and the more active 4-hydroxybenzhydrazone derivatives, an enzymatic assay with *T. cruzi* cysteine proteases was performed (Table 2). **Nfz** had a good inhibition on cruzain. Compounds **Nfz-1** and **Nfz-8** had a strong inhibition with IC_50_ values of 17.6 µM and 4.6 µM, respectively.

### 3.4. Leishmanicidal Activity

**Nfz** and 4-hydroxybenzhydrazone derivatives had variable biological behavior against both strains of *L. mexicana* (Table 1). **Nfz-5** (IC_50_ = 5.2 μM) and **Nfz-14** (IC_50_ = 10.2 μM) had the best leishmanicidal activity against the M379 and FCQEPS strains, respectively. Also, **Nfz-12** and **Nfz-23** had good activity against the M379 strain (IC_50_ < 20 μM). Additionally, ten 4-hydroxybeznhydrazone derivatives had a better biological effect than glucantime (IC_50_ = 133.9 μM) against the M379 strain. On the other hand, **Nfz-1** only had an IC_50_ value below 40 μM against the FCQEPS strain. Therefore, **Nfz-1**, **Nfz-12**, and **Nfz-23** could be structurally optimized to increase their potency.

### 3.5. Cytotoxicity on Macrophages

**Nfz** and the 4-hydroxybeznhydrazone derivatives had variable cytotoxicity values, ranging from 2.45 to >200 μM (Table 3). Three compounds were cytotoxic to the cell macrophages, with CC_50_ < 29 μM, **Nfz-18** (4-nitrophenyl) being the most toxic. Compounds with less cytotoxicity displayed CC_50_ values greater than 100 μM. The selectivity index (SI) was determined for all compounds, including the reference drugs (Table 3).

### 3.6. Anti-Mycobacterium Activity

To explore the antimicrobial properties, all compounds were tested for their in vitro growth inhibitory activity against the human pathogen *Mycobacterium tuberculosis* H37Rv strain (Table 4).

## 4. Discussion

### 4.1. Structure–Activity Relationship (SAR) Against T. cruzi

**Figure 3:**
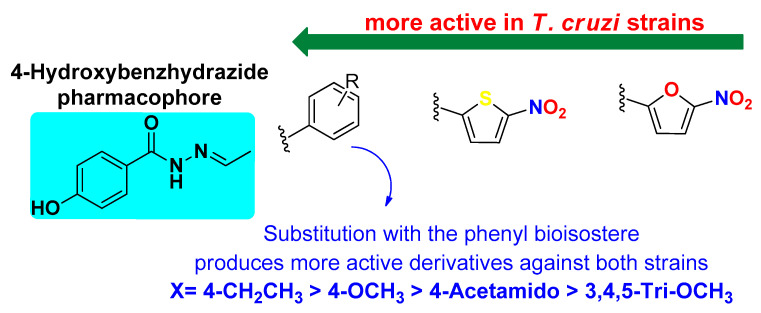
SAR analysis of Nfz and **4-hydroxybenzhydrazone** derivatives against *T. cruzi*. The blue square highlights the retained 4-Hydroxybenzhydrazone pharmacophore, and the green arrow indicates the substituents most active.

A SAR analysis against the epimastigotes of the NINOA strain was carried out to discover the effects of different substituents (Figure 3). The **Nfz** was the starting point with a trypanocidal activity of 36 µM. The replacement of the 5-nitrofuran ring with 5-nitrothiophene in **Nfz-1** improved the activity three times more (IC_50_ = 11.9 μM). This may be because the thiophene heterocycle exhibits better lipophilicity compared to furan [28]. The substitution of the -NO_2_ group with bromine at the 5-position on the thienyl ring (**Nfz-2**) substantially increases the biological activity (IC_50_ = 4.7 µM). The unsubstituted thienyl ring (**Nfz-3**) was poorly active (IC_50_ > 50 µM). Compound **Nfz-4** with the furyl ring without nitro group had better trypanocidal activity (IC_50_ = 18.8 µM) than Nfz and Bzn (IC_50_ = 30 µM). However, the addition of the *N*-methyl-pyrrole ring in **Nfz-5** caused inactivity. The phenyl ring was introduced as a bioisostere of the furyl and thienyl rings; however, compound **Nfz-6** was inactive. However, the addition of a methyl group (**Nfz-7**) and an ethyl group (**Nfz-8**) at the *para*-position improved the trypanocidal activity, with IC_50_ values of 26 and 14.4 µM, respectively. Compound **Nfz-9**, substituted with an electron-donating group (−OH) at the *para*-position, had an activity comparable to **Nfz-7**. However, the same group at the *ortho*-position (**Nfz-10**) drastically diminished the effect against epimastigotes (IC_50_ > 100 µM), suggesting that steric effects are key in the trypanocidal activity. **Nfz-11**, with the methoxy group (electron-donating) at the *para*-position on the phenyl ring, enhanced the activity with an IC_50_ value of 28.2 µM, like the drug Bzn. Remarkably, the methoxy group at the *ortho*-position (**Nfz-12**) caused inactivity, while **Nfz-13** with the methoxy group at the *ortho-* and *meta*-position had an IC_50_ value greater than 60 µM. However, **Nfz-14** with the trimethoxy group exhibited the same trypanocidal effect as **Nfz-11**. These findings suggest that *ortho* and *ortho*/*meta* substitutions with methoxy groups are unfavorable for the activity against *T. cruzi*. The substitutions with the fluorine (**Nfz-15**), chlorine (**Nfz-16**), and bromine (**Nfz-17**) atoms at the *para*-position on the phenyl ring caused a loss of trypanocidal activity. **Nfz-18** substituted with the nitro group at the *para*-position had an IC_50_ value less (44.5 µM) than Bzn; unexpectedly, the incorporation of the nitro group at the *ortho*-position (**Nfz-19**) improved the activity (IC_50_ = 36.4 µM). Compounds **Nfz-20**, **Nfz-21**, **Nfz-22**, and **Nfz-23** with bulky substituents such as 2-naphthyl, 2-benzodioxanyl, 4-biphenyl, and 6-nitropiperonyl, respectively, caused a loss of the activity, except **Nfz-22** (IC_50_ = 6.8 µM), which had a potent trypanocidal effect. Finally, compound **Nfz-24** (IC_50_ = 22.1 µM), with the acetamido moiety at the *para*-position had the same activity as **Nfz-8**. The compound **Nfz-25** with the methyl 4-formylbenzoate group had no activity.

On the other hand, **Nfz** and **Nfz-1** had a strong trypanocidal (IC_50_ < 15 µM) effect against the A1 strain. However, the substitution of the nitro group by a bromine atom (**Nfz-2**) decreases drastically the biological activity (IC_50_ > 100 µM). However, **Nfz-3** (IC_50_ = 19.9 µM) with an unsubstituted thienyl ring had similar activity to **Nfz-2**. Likewise, **Nfz-4** with the furyl ring had similar activity (IC_50_ = 28.8 µM) to Bzn. Once again, the compound **Nfz-5** was inactive (IC_50_ > 100 µM). These results suggest that the nitro group on the heterocycle ring is essential for the trypanocidal activity against the A1 strain.

On the other hand, the change of nitrofuran by phenyl ring (**Nfz-6**) and the addition of methyl group at the *para*-position on the phenyl ring (**Nfz-7**) does not retain the trypanocidal activity (IC_50_ > 70 μM). Meanwhile, the incorporation of the *para*-ethyl group (**Nfz-8**) had a strong trypanocidal effect against the A1 strain (IC_50_ = 7.2 μM), even better than Nfx (IC_50_ = 19.3 µM). Therefore, the elongation of the aliphatic chain at the *para*-position on the phenyl ring significantly improves the trypanocidal activity. Compound **Nfz-9** substituted with hydroxy group at *para*-position was unfavorable (IC_50_ > 100 µM). Surprisingly, the same group at the *ortho*-position had a strong effect against *T. cruzi* epimastigotes (**Nfz-10**, IC_50_ = 8.7 µM). Regarding the compounds with methoxy substitutions, only **Nfz-11** (IC_50_ = 18.1 µM) and **Nfz-14** (IC_50_ = 29.2 µM) exhibited trypanocidal activity comparable to the reference drugs Bzn and Nfx. Derivatives with halogen substituents **Nfz-15**, **Nfz-16**, and **Nfz-17** evidenced trypanocidal activity below 50 µM. Compound **Nfz-18** with the *para*-nitro phenyl was inactive (IC_50_ > 100 µM); however, the nitro group at the *ortho*-position improved the biological effects (**Nfz-19**, IC_50_ = 42.4 µM). For derivatives **Nfz-20**, **Nfz-21**, **Nfz-22**, and **Nfz-23** incorporating bulky groups, only derivatives **Nfz-20** (IC_50_ = 24.4 µM) and **Nfz-23** (IC_50_ = 13.5 µM) exhibited a trypanocidal effect comparable to the drugs Bzn and Nfx. Finally, compound **Nfz-24** (IC_50_ = 22.1 µM), with the acetamido group, had trypanocidal activity comparable to Nfx and better than Bzn, while **Nfz-25** (methyl 4-formylbenzoate) was inactive.

### 4.2. Structure–Activity Relationship (SAR) Against L. mexicana

**Figure 4:**
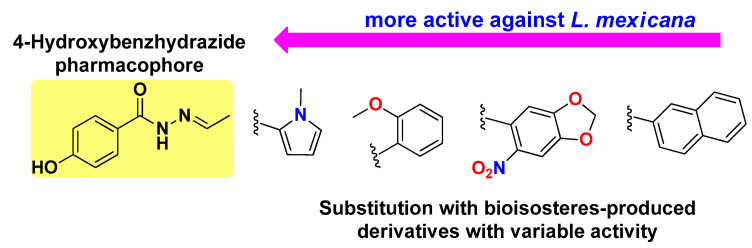
SAR analysis of Nfz and the 4-hydroxybenzhydrazone derivatives against *L. mexicana.* The yellow square highlights the retained 4-Hydroxybenzhydrazone pharmacophore, and the pink arrow indicates the substituents most active.

The SAR analysis against *L. mexicana* showed that the heterocyclic moiety exhibited variable behavior in both strains (Figure 4). **Nfz** and the derivatives **Nfz-1**, **Nfz-2**, and **Nfz-3** had no leishmanicidal effects against strain M379. Only **Nfz-5** with an *N*-methyl-pyrrole ring had a potent leishmanicidal effect (IC_50_ = 5.2 μM). On the other hand, the derivatives **Nfz-6**, **Nfz-7**, and **Nfz-8** were inactive (IC_50_ > 90 μM). These findings suggest that aliphatic substituents do not contribute to the leishmanicidal activity against the M379 strain. Compounds with the *para*-hydroxy phenyl (**Nfz-9**) and *ortho*-hydroxy phenyl (**Nfz-10**) had IC_50_ values greater than 50 µM. Hence, these substitutions were not favorable in terms of potency. For the derivatives with methoxy substituents, only **Nfz-12** had a ten-fold better activity (IC_50_ = 11.5 µM) than glucantime (IC_50_ = 133.9 µM). Compound **Nfz-15** (4-fluorophenyl) had an IC_50_ value in the 30 µM range, while **Nfz-16** and **Nfz-17** exhibited activity greater than 60 µM. No significant leishmanicidal activity was observed for **Nfz-18** and **Nfz-19** (IC_50_ > 100 µM). A similar pattern was noted for compounds with bulky substituents (**Nfz-21** and **Nfz-22**). Only **Nfz-20** and **Nfz-23** had good leishmanicidal activity, with IC_50_ values below 30 µM. Compound **Nfz-24** (4-acetamidophenyl) presented an IC_50_ value of 54.1 µM, while **Nfz-25** (methyl 4-formylbenzoate) had an improved leishmanicidal effect.

On the other hand, the FCQEPS strain was less susceptible to the **Nfz** and the 4-hydroxybenzhydrazone derivatives. **Nfz-1** with the bioisostere replacement (5-nitrothienyl) had good activity compared to the drug glucantime (IC_50_ > 120 µM). Interestingly, **Nfz-14** with the trimethoxy group on the phenyl ring had the best leishmanicidal activity (IC_50_ = 10.2 µM). The rest of the compounds demonstrated lower activities (>50 µM). These results suggest that the FCQEPS strain exhibits resistance to most of the molecules evaluated.

### 4.3. Cytotoxicity and Selectivity Index (SI) on Macrophages

According to the results, **Nfz** and the 4-hydroxybenzhydrazone derivatives had variable cytotoxicity values, ranging from 2.45 to >200 μM (Table 2). Compound **Nfz-18** (4-nitrophenyl) was the most toxic. The compounds with less cytotoxicity were **Nfz-3**, **Nfz-4**, **Nfz-8**, **Nfz-10**, **Nfz-13**, **Nfz-14**, **Nfz-17**, **Nfz-19**, **Nfz-21**, **Nfz-24**, and **Nfz-25** (CC_50_ >100 μM).

The selectivity index (SI) was calculated for all compounds, including the reference drugs. For the A1 strain of *T. cruzi*, **Nfz** had an SI of 4, while **Nfz-8** (SI > 13.7) and **Nfz-15** (SI = 15.6) showed the best SI values compared to Bzn (SI = 3.4). **Nfz-24**, which demonstrated moderate trypanocidal activity, had SI values greater than 7 for both strains. Future studies on this compound could focus on improving its potency and selectivity. For the NINOA strain, compounds **Nfz-2** and **Nfz-24** exhibited SI values greater than Bzn (SI = 4.4) and even exceeded those recommended in the literature [29]. Lastly, **Nfz-22** (Biphenyl-bulky group) demonstrated a strong activity only against the NINOA strain of *T. cruzi*, with an SI value greater than 6. Based on these findings, our results suggest that **Nfz-8** and **Nfz-24** derivatives are promising structures for optimization to enhance their potential therapeutic profile.

### 4.4. Inhibition of T. cruzi Cysteine Protease Activity

An enzymatic assay with *T. cruzi* cysteine proteases revealed that compounds **Nfz-1** and **Nfz-8** had strong inhibition, with IC_50_ values in the micromolar range. Based on the above, it can be inferred that their trypanocidal activity is due to cruzain inhibition. These results are highlighted compared to previous reports for related N-acylhydrazone derivatives (IC_50_ > 40 µM) [30]. Finally, compounds **Nfz**, **Nfz-4**, and **Nfz-11** with trypanocidal activity did not inhibit the proteases, suggesting that their mechanism of action is not directly related to cruzain.

### 4.5. Anti-Mycobacterium Activity

**Figure 5:**
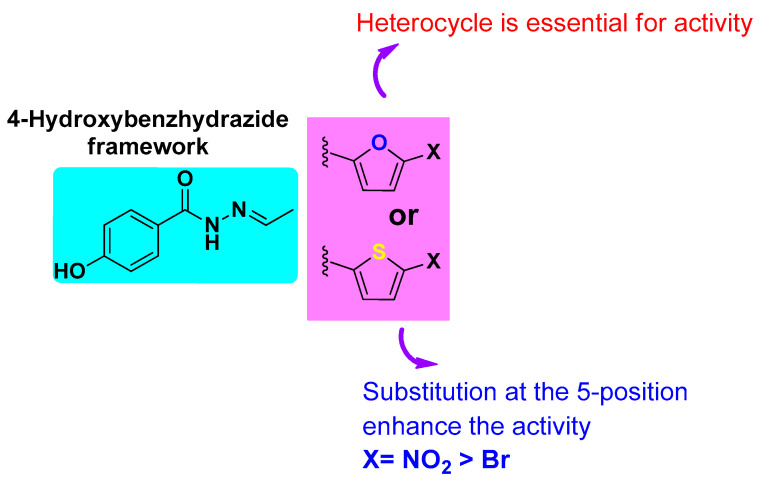
SAR analysis of **Nfz** and its analogs against the *M. tuberculosis* H37Rv strain. The blue square highlights the retained 4-Hydroxybenzhydrazone pharmacophore, and the pink square indicates the heterocycle substituents more active.

Compounds with the best anti-*M. tuberculosis* effects (MIC < 20 µg/mL) were **Nfz**, **Nfz-1**, and **Nfz-2**, which contain a nitro-substituted heterocycle (furyl or thienyl). Additionally, compounds **Nfz-10**, **Nfz-16**, **Nfz-17**, and **Nfz-25** had an MIC < 50 µg/mL, while the rest of the derivatives had lower activity. The SAR analysis exposed that the 4-hydroxybenzhydrazide fragment combined with 5-nitrothienyl was the most potent compared to its analogue 5-nitrofuryl. Furthermore, incorporating a bromine atom at the 5-position of thienyl slightly decreased the activity against *M. tuberculosis*. Finally, none of the twenty-five compounds reported here outperformed the reference drugs. Figure 5 summarizes the short SAR against *M. tuberculosis* of **Nfz** and their analogs. However, **Nfz**, **Nfz-1**, and **Nfz-2** are interesting molecules to continue studying to improve their structural optimization and antimicrobial activity.

## 5. Conclusions

In this study, **Nfz** and twenty-five 4-hydroxybenzhydrazone derivatives were easily obtained with good yields. **Nfz** and its analogs **Nfz-1**, **Nfz-4**, **Nfz-8**, **Nfz-9**, **Nfz-11**, and **Nfz-13** had better micromolar trypanocidal activities than reference drugs against two strains of *T. cruzi*. Furthermore, **Nfz-2** and **Nfz-24** exhibited potent strain-specific activity against NINOA. Meanwhile, **Nfz-15** was selective for the A1 strain. Finally, the enzymatic assays demonstrated the mode of action of **Nfz-1** and **Nfz-8** as cysteine protease inhibitors. Therefore, these compounds could serve as a scaffold for developing more potent and selective drugs against *T. cruzi*, targeting cysteine proteases with minimal cytotoxicity (CC_50_ > 100 µM).

On the other hand, **Nfz-5**, **Nfz-10**, and **Nfz-25** had potent antiparasitic activity against the reference strain M379 of *L. mexicana*. Compound **Nfz-13** was highlighted as the most effective against the FCQEPS strain. These findings encourage us to continue studying the potential of **Nfz** and the 4-hydroxybenzhydrazone derivatives’ bearing as leishmanicidal agents and to elucidate the mechanism of action against strains of *L. mexicana.*

Finally, **Nfz** had an acceptable effect against *M. tuberculosis*, highlighting **Nfz-1** as more potent with a MIC value of 5.1 µg/mL. Therefore, this compound is a promising candidate for further studies in the development of anti-mycobacterial agents. These results contribute to the study of **Nfz** and the 4-hydroxybenzhydrazone derivatives as new anti-*T. cruzi*, anti-*L. mexicana,* and anti-*M. tuberculosis* agents, encouraging future research aimed at structural optimization to develop new and more potent drugs for these infectious diseases.

## Figures and Tables

**Table 1:** Half-maximal inhibitory concentration (IC_50_ in µM) of Nfz and 4-hydroxybenzhydrazone derivatives against *T. cruzi* and *L. mexicana* strains.

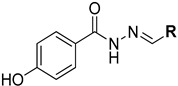					
Compound	-R	*T. cruzi*		*L. mexicana*	
		NINOA (µM)	A1 (µM)	^1^ M379 (µM)	^2^ FCQEPS (µM)
Nfz	5-Nitro-furyl	36.00 ± 1.2	10.41 ± 1.6	>200	51.17 ± 4.2
**Nfz-1**	5-Nitro-thienyl	11.94 ± 3.2	14.00 ± 1.9	>200	32.91 ± 7.0
**Nfz-2**	5-Bromo-thienyl	4.77 ± 5.6	>100	>200	98.83 ± 5.2
**Nfz-3**	Thienyl	51.00 ± 2.8	19.97 ± 1.9	>200	49.79 ± 5.0
**Nfz-4**	Furyl	18.99 ± 2.1	28.89 ± 3.4	83.90 ± 6.9	>200
**Nfz-5**	*N*-Methylpyrrole	>100	78.52 ± 4.7	5.25 ± 5.8	>200
**Nfz-6**	Phenyl	>100	74.39 ± 4.4	94.22 ± 0.9	>200
**Nfz-7**	4-Methylphenyl	26.02 ± 2.9	75.17 ± 8.8	>200	102.77 ± 2.1
**Nfz-8**	4-Ethylphenyl	14.45 ± 4.4	7.28 ± 2.2	>200	>200
**Nfz-9**	4-Hidroxyl	24.88 ± 3.2	>100	>200	94.28 ± 5.9
**Nfz-10**	2-Hidroxyl	>100	8.77 ± 1.6	66.71 ± 2.9	>200
**Nfz-11**	4-Methoxyphenyl	28.23 ± 1.4	18.15 ± 1.4	>200	>200
**Nfz-12**	2-Methoxyphenyl	>100	>100	11.56 ± 2.1	60.33 ± 4.7
**Nfz-13**	2,3-diMethoxyphenyl	63.09 ± 1.4	>100	161.69 ± 6.9	124.52 ± 1.1
**Nfz-14**	3,4,5-TriMethoxyphenyl	28.02 ± 1.9	29.23 ± 4.4	>200	10.28 ± 0.8
**Nfz-15**	4-Fluorophenyl	>100	32.63 ± 2.8	35.09 ± 4.7	101.72 ± 2.7
**Nfz-16**	4-Chlorophenyl	>100	40.22 ± 2.7	67.54 ±4.0	78.09 ± 3.6
**Nfz-17**	4-Bromophenyl	>100	33.59 ± 3.9	62.03 ± 2.5	52.12 ± 4.8
**Nfz-18**	4-Nitrophenyl	44.58 ± 6.3	>100	>200	57.77 ± 2.6
**Nfz-19**	2-Nitrophenyl	36.45 ± 2.2	42.46 ± 6.7	146.72 ± 0.7	>200
**Nfz-20**	2-Naphtyl	>100	24.48 ± 2.0	28.45 ± 2.8	>200
**Nfz-21**	2-benzodioxanyl	>100	49.04 ± 2.1	71.51 ± 4.9	>200
**Nfz-22**	4-Biphenyl	6.87 ± 1.2	>100	>200	133.16 ± 1.4
**Nfz-23**	6-Nitropiperonyl	>100	13.52 ± 3.4	15.96 ± 2.0	>200
**Nfz-24**	4-Acetamidophenyl	14.18 ± 3.5	22.19 ± 1.4	54.16 ± 4.0	55.76 ± 3.5
**Nfz-25**	Methyl 4-formylbenzoate	>100	>100	32.53 ± 6.1	>200
Benznidazole		30.3 ± 0.3	39.08 ± 0.7	-	-
Nifurtimox		7.09 ± 0.2	19.30 ± 0.8		
Glucantime		-	-	133.96 ± 3.1	125.23 ± 1.6

^1^ M379: MNYC/BZ/62/M379 TCC; ^2^ FCQEPS: MHOM/MX/2017/UABJO17FCQEPS.

**Table 2:** The half-maximal inhibitory concentration (IC_50_ in µM) of Nfz and 4-hydroxybenzhydrazone derivatives on cysteine proteases of *T. cruzi*.

Compound	Structure	IC_50_ in µM
Nfz	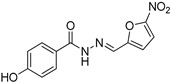	89.49
**Nfz-1**	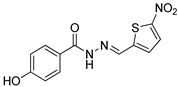	17.64
**Nfz-4**	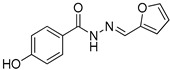	69.95
**Nfz-8**	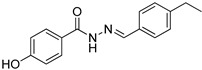	4.60
**Nfz-11**	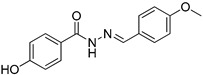	3805.1

**Table 3:** Cytotoxicity and selectivity index profile of Nfz and 4-hydroxybenzhydrazone derivatives.

Compound	−R	Macrophages J774.2 (µM)	*T. cruzi* SI		*L. mexicana* SI	
			NINOA	A1	M379	FCQEPS
Nfz	5-NO_2_-Furyl	42.23 ± 2.3	1.17	4.05	0.21	0.82
**Nfz-1**	5-NO_2_-Thienyl	43.27 ± 2.7	3.62	3.09	<0.21	1.21
**Nfz-2**	5-Br-Thienyl	95.48 ± 4.2	20.01	<0.95	<0.47	0.96
**Nfz-3**	Thienyl	213.75 ± 2.9	4.19	10.70	<1.06	4.29
**Nfz-4**	Furyl	>100	>5.26	>3.46	<1.19	<0.50
**Nfz-5**	*N*-Methylpyrrole	21.89 ± 4.4	<0.21	0.27	4.16	>0.10
**Nfz-6**	C_6_H_5_	28.77 ± 1.5	<0.28	0.38	0.30	>0.14
**Nfz-7**	4-Methylphenyl	91.68 ± 5.1	3.52	1.21	<0.45	0.89
**Nfz-8**	4-Ethylphenyl	>100	>6.92	>13.73	<0.50	<0.50
**Nfz-9**	4-Hidroxyl	82.71 ± 4.4	3.32	<0.82	<0.41	0.80
**Nfz-10**	2-Hidroxyl	137.17 ± 2.6	<1.37	15.64	2.05	>0.68
**Nfz-11**	4-Methoxyphenyl	46.59 ± 4.9	1.65	2.56	<0.23	<0.23
**Nfz-12**	2-Methoxyphenyl	76.45 ± 3.6	>0.76	>0.76	6.61	1.26
**Nfz-13**	2,3-diMethoxyphenyl	120.29 ± 0.2	1.91	<1.20	0.74	0.96
**Nfz-14**	3,4,5-TriMethoxyphenyl	126.75 ± 6.5	4.52	4.33	<0.63	12.32
**Nfz-15**	4-Fluorine	74.00 ± 3.4	<0.74	2.26	2.10	0.72
**Nfz-16**	4-Chlorine	83.24 ± 3.2	<0.83	2.06	1.23	1.06
**Nfz-17**	4-Bromine	>100	1.00	>2.97	<1.61	<1.91
**Nfz-18**	4-Nitro	2.45 ± 2.9	0.05	<0.02	<0.01	0.04
**Nfz-19**	2-Nitro	>100	>2.74	>2.35	<0.68	<0.50
**Nfz-20**	2-Naphtyl	88.30 ± 3.9	<0.88	3.60	3.10	>0.44
**Nfz-21**	2-benzodioxanyl	>100	1.00	>2.03	<1.39	<0.50
**Nfz-22**	4-Biphenyl	43.49 ± 2.5	6.33	<0.43	<0.21	0.32
**Nfz-23**	6-Nitropiperonyl	68.87 ± 6.6	<0.68	5.09	4.31	>0.34
**Nfz-24**	4-Acetamidophenyl	173.37 ± 5.2	12.22	7.81	3.20	3.10
**Nfz-25**	Methyl 4-formylbenzoate	>100	1.00	1.00	<3.07	<0.50
Benznidazole		133.90 ± 0.2	4.41	3.42	-	-
Glucantime		>200	-	-	>2.03	>2.18

**Table 4:** Anti-mycobacterial activity of Nfz and 4-hydroxybenzhydrazone derivatives.

Compound	MABA MIC (µg/mL)
Nfz	12.3
**Nfz-1**	5.1
**Nfz-2**	18.8
**Nfz-3**	>100
**Nfz-4**	>100
**Nfz-5**	>100
**Nfz-6**	>100
**Nfz-7**	98.3
**Nfz-8**	79.4
**Nfz-9**	94.1
**Nfz-10**	47.6
**Nfz-11**	>100
**Nfz-12**	>100
**Nfz-13**	157.8
**Nfz-14**	>200
**Nfz-15**	>100
**Nfz-16**	46.4
**Nfz-17**	43.6
**Nfz-18**	186.4
**Nfz-19**	>100
**Nfz-20**	>100
**Nfz-21**	68.7
**Nfz-22**	>100
**Nfz-23**	>100
**Nfz-24**	>100
**Nfz-25**	45.9
Isoniazid	0.039
Rifampin	0.074
Moxifloxacin	0.184
Linezolid	0.330

## Data Availability

Data is contained within the article and Supplementary Materials.

## Supplementary Materials

The following supporting information can be downloaded at https://www.mdpi.com/article/10.3390/pharmaceutics17050621/s1. The Supplementary Materials correspond to the spectral data of infrared spectrometry (FT-IR), nuclear magnetic resonance (NMR), and UPLC-MS of the compounds synthesized.

## References

[1] Brune K. (2022). Chemistry and Pharmacology of Nitrofurans. J. Med. Chem..

[2] Gupta K., Hooton T.M., Naber K.G. (2017). Antimicrobial Use for Urinary Tract Infections: The Role of Nitrofurantoin. Clin. Infect. Dis..

[3] Sok D., Hwang Y.J., Yoon J. (2020). Safety and Efficacy of Nitrofurantoin in Clinical Practice. Therapeut. Clin. Risk Manag..

[4] Nicolas M., McEwan G., Brown J. (2021). Advances in Nitrofurans and Their Therapeutic Potential. Expert Rev. Anti-Infect. Ther..

[5] Roquini V., Mengarda A.C., Cajas R.A., Martins-da-Silva M.F., Godoy-Silva J., Santos G.A., Espírito-Santo M.C.C., Pavani T.F., Melo V.A., Salvadori M.C. (2023). The Existing Drug Nifuroxazide as an Antischistosomal Agent: In Vitro, In Vivo, and In Silico Studies of Macromolecular Targets. Microbiol. Spectrum.

[6] Toledano-Magaña Y., García-Ramos J.G., Navarro-Olivarria M., Flores-Alamo M., Manzanera-Estrada M., Ortiz-Frade L., Galindo-Murillo R., Ruiz-Azuara L., Meléndrez-Luevano R., Cabrera-Vivas B.M. (2015). Potential Amoebicidal Activity of Hydrazone Derivatives: Synthesis, Characterization, Electrochemical Behavior, Theoretical Study, and Evaluation of the Biological Activity. Molecules.

[7] Said E., Zaitone S.A., Eldosoky M., Elsherbiny N.M. (2018). Nifuroxazide, a STAT3 Inhibitor, Mitigates Inflammatory Burden and Protects Against Diabetes-Induced Nephropathy in Rats. Chem.-Biol. Interact..

[8] Luo L., Xu F., Peng H., Luo Y., Tian X., Battaglia G., Zhang H., Gong Q., Gu Z., Luo K. (2020). Stimuli-Responsive Polymeric Prodrug-Based Nanomedicine Delivering Nifuroxazide and Doxorubicin Against Primary Breast Cancer and Pulmonary Metastasis. J. Control. Release.

[9] Popiołek Ł. (2021). Updated Information on Antimicrobial Activity of Hydrazide–Hydrazones. Int. J. Mol. Sci..

[10] Kannigadu C., Aucamp J., N’Da D.D. (2022). Exploring Novel Nitrofuranyl Sulfonohydrazides as Anti-Leishmania and Anti-Cancer Agents: Synthesis, In Vitro Efficacy and Hit Identification. Chem. Biol. Drug Des..

[11] Atta A., Fahmy S., Rizk O., Sriram D., Mahran M.A., Labouta I.M. (2018). Structure-Based Design of Some Isonicotinic Acid Hydrazide Analogues as Potential Antitubercular Agents. Bioorg. Chem..

[12] Teneva Y., Simeonova R., Valcheva V., Angelova V.T. (2023). Recent Advances in Anti-Tuberculosis Drug Discovery Based on Hydrazide-Hydrazone and Thiadiazole Derivatives Targeting InhA. Pharmaceuticals.

[13] Sidorov N.G., Kravchenko A.D., Poddubikov A.V., Arzumanian V.G. (2019). Synthesis and Study of the Antimicrobial Activity of Nifuroxazide Derivatives. Microbiol. Indep. Res. J..

[14] Masunari A., Tavares L.C. (2007). A New Class of Nifuroxazide Analogues: Synthesis of 5-Nitrothiophene Derivatives with Antimicrobial Activity Against Multidrug-Resistant *Staphylococcus aureus*. Bioorg. Med. Chem..

[15] Bailly C. (2019). Toward a Repositioning of the Antibacterial Drug Nifuroxazide for Cancer Treatment. Drug Discov. Today.

[16] Gan C., Zhang Q., Liu H., Wang G., Wang L., Li Y., Tan Z., Yin W., Yao Y., Xie Y. (2022). Nifuroxazide Ameliorates Pulmonary Fibrosis by Blocking Myofibroblast Genesis: A Drug Repurposing Study. Respir. Res..

[17] Kaiser M., Mäser P., Tadoori L.P., Ioset J.R., Brun R. (2015). Antiprotozoal Activity Profiling of Approved Drugs: A Starting Point Toward Drug Repositioning. PLoS ONE.

[18] Paula F.R., Jorge S.D., de Almeida L.V., Pasqualoto K.F., Tavares L.C. (2009). Molecular Modeling Studies and In Vitro Bioactivity Evaluation of a Set of Novel 5-Nitro-Heterocyclic Derivatives as Anti-*T. cruzi* Agents. Bioorg. Med. Chem..

[19] Kannigadu C., Aucamp J., N’Da D.D. (2021). Synthesis and In Vitro Antileishmanial Efficacy of Benzyl Analogues of Nifuroxazide. Drug Dev. Res..

[20] Badenhorst G.D., Kannigadu C., Aucamp J., David D.D. (2022). Probing O-Substituted Nifuroxazide Analogues Against *Leishmania*: Synthesis, In Vitro Efficacy, and Hit/Lead Identification. Eur. J. Pharm. Sci..

[21] Nepali K., Lee H.Y., Liou J.P. (2018). Nitro-Group-Containing Drugs. J. Med. Chem..

[22] Franzblau S.G., Witzig R.S., McLaughlin J.C., Torres P., Madico G., Hernandez A., Degnan M.T., Cook M.B., Quenzer V.K., Ferguson R.M. (1998). Rapid, Low-Technology MIC Determination with Clinical *Mycobacterium tuberculosis* Isolates by Using the Microplate Alamar Blue Assay. J. Clin. Microbiol..

[23] Palos I., Luna-Herrera J., Lara-Ramírez E.E., Loera-Piedra A., Fernández-Ramírez E., Aguilera-Arreola M.G., Paz-González A.D., Monge A., Wan B., Franzblau S. (2018). Anti-*Mycobacterium tuberculosis* Activity of Esters of Quinoxaline 1,4-Di-N-Oxide. Molecules.

[24] Bradford M.M. (1976). A Rapid and Sensitive Method for the Quantitation of Microgram Quantities of Protein Utilizing the Principle of Protein-Dye Binding. Anal. Biochem..

[25] Santos C.C., Sant’anna C., Terres A., Cunha-e-Silva N.L., Scharfstein J., de A. Lima A.P. (2005). Chagasin, the Endogenous Cysteine-Protease Inhibitor of *Trypanosoma cruzi*, Modulates Parasite Differentiation and Invasion of Mammalian Cells. J. Cell Sci..

[26] Ritz C., Baty F., Streibig J.C., Gerhard D. (2016). Dose-Response Analysis Using R. PLoS ONE.

[27] Rando D.G., Avery M.A., Tekwani B.L., Khan S.I., Ferreira E.I. (2008). Antileishmanial Activity Screening of 5-Nitro-2-Heterocyclic Benzylidene Hydrazides. Bioorg. Med. Chem..

[28] Hollósy F., Seprödi J., Orfi L., Erös D., Kéri G., Idei M. (2002). Evaluation of Lipophilicity and Antitumor Activity of Parallel Carboxamide Libraries. J. Chromatogr. B Analyt. Technol. Biomed. Life Sci..

[29] Indrayanto G., Satrio Putra G., Suhud F., Al-Majed A.A. (2021). Chapter Six—Validation of In-Vitro Bioassay Methods: Application in Herbal Drug Research. Profiles of Drug Substances, Excipients and Related Methodology.

[30] Carvalho S.A., Feitosa L.O., Soares M., Costa T.E., Henriques M.G., Salomão K., de Castro S.L., Kaiser M., Brun R., Wardell J.L. (2012). Design and Synthesis of New (E)-Cinnamic N-Acylhydrazones as Potent Antitrypanosomal Agents. Eur. J. Med. Chem..

