# Supplementary material for: Nifuroxazide and 4-Hydroxybenzhydrazone Derivatives as New Antiparasitic (Trypanosoma cruzi and Leishmania mexicana) and Anti-Mycobacterium tuberculosis Agents
Source: Pharmaceutics. 2025 May 7;17(5):621. doi: 10.3390/pharmaceutics17050621 (PMC12114619; doi:10.3390/pharmaceutics17050621)
Supplement: Supplementary file 1 [file pharmaceutics-17-00621-s001.zip › pharmaceutics-3585353-supplementary.pdf]

Nifuroxazide and Their Analogues as New Antiparasitic (*Trypanosoma cruzi* and *Leishmania mexicana*) and Anti-*Mycobacterium tuberculosis* Agents

Timoteo Delgado-Maldonado[1], Diana V. Navarrete-Carriola[1], Lenci K. Vázquez-Jiménez[1], Alma D. Paz-González[1], Baojie Wan[2], Scott Franzblau[2], Othman Mueen Alkubaisi[3], Lorena Rodriguez-Paez[4], Charmina Aguirre-Alvarado[4], Verónica Alcántara-Farfán[4], Joaquín Cordero-Martínez[4], Debasish Bandyopadhyay[5], Gildardo Rivera[1*], and Adriana Moreno-Rodríguez[5*]

[1]Laboratorio de Biotecnología Farmacéutica, Centro de Biotecnología Genómica, Instituto Politécnico Nacional, 88710, Reynosa, México; titi_999@live.com (T.D.-M.); giriveras@ipn.mx (G.R.); (D.V.N.C.); (L.K.V.J.); (A.D.P.G.)

[2]Institute for Tuberculosis Research, College of Pharmacy, University of Illinois at Chicago, 833 S. Wood St., Chicago, IL 60612, USA; (B.W.); (S.F.)

[3]Department of Medical Laboratories Techniques, College of Health and Medical Technology, University of Al Maarif, Al Anbar, 31001, Iraq; (O.M.A.)

[4]Departamento de Bioquímica, Escuela Nacional de Ciencias Biológicas, Instituto Politécnico Nacional, Ciudad de México 11340, México; (L.R.P.); (C.A.A); (V.A.F.); (J.C.M.)

[5]School of Integrative Biological and Chemical Sciences (SIBCS) & School of Earth, Environmental, and Marine Sciences (SEEMS), University of Texas Rio Grande Valley, Edinburg, TX 78539, USA

[6]Laboratorio de Estudios Epidemiológicos, Clínicos, Diseños Experimentales e Investigación, Facultad de Ciencias Químicas, Universidad Autónoma "Benito Juárez" de Oaxaca, Avenida Universidad S/N, Ex Hacienda Cinco Señores, Oaxaca 68120, México; arimor@hotmail.com (A.M-R.)

*Correspondence: giriveras@ipn.mx; arimor@hotmail.com

Supplementary Material

The supplementary material corresponds to the spectral data of infrared spectrometry (FT-IR), nuclear magnetic resonance (NMR), and UPLC-MS of the compounds synthesized.

(E)-4-hydroxy-N′-((5-nitrofuran-2-yl)methylene)benzohydrazide (**Nfz**). This compound was obtained in 84.98 % from 4-Hydroxybenzhydrazide and 5-Nitro-2-furaldehyde. FT-IR (v cm^{-1}): 3365 (N-H), 1671 (C=O), 1606 (C=N). 1H NMR (500 MHz, DMSO) δ 12.04 (s, 1H), 10.26 (s, 1H), 8.38 (s, 1H), 7.93 – 7.84 (m, 1H), 7.31 (d, J = 3.9 Hz, 2H), 7.25 (d, J = 3.9 Hz, 1H), 6.89 (t, J = 8.0 Hz, 2H). m/z calculated: 275.22 Da, found: 276.04 [M+H] Da.

(E)-4-hydroxy-N′-((5-nitrothiophen-2-yl)methylene)benzohydrazide (**Nfz-1**). This compound was obtained in 47.21 % from 4-Hydroxybenzhydrazide and 5-Nitro-2-thiophenecarboxaldehyde. FT-IR (v cm^{-1}): 3368 (N-H), 1665 (C=O), 1603 (C=N). ^{1}H NMR (500 MHz, DMSO) δ 12.03 (s, 1H), 10.04 (s, 1H), 8.67 (s, 1H), 8.13 (d, J = 4.3 Hz, 1H), 7.82 (s, 1H), 7.69 (d, J = 8.2 Hz, 1H), 7.56 (d, J = 4.3 Hz, 1H), 6.87 (s, 1H), 6.78 (d, J = 8.2 Hz, 1H). m/z calculated: 291.28 Da, found: 291.98 [M+H] Da.

(E)-N′-((5-bromothiophen-2-yl)methylene)-4-hydroxybenzohydrazide (**Nfz-2**). This compound was obtained in 92.95 % from 4-Hydroxybenzhydrazide and 5-Bromo-2-thiophenecarboxaldehyde. FT-IR (v cm^{-1}): 3230 (N-H), 1621 (C=O), 1600 (C=N). ^{1}H-NMR (500 MHz, DMSO) δ 11.68 (s, 1H), 10.23 (s, 1H), 8.55 (s, 1H), 7.77 (d, J = 8.3 Hz, 2H), 7.30–7.23 (m, 2H), 6.86 (d, J = 8.3 Hz, 2H). m/z calculated: 325.18 Da, found: 325.49 [M], 327.47 [M+2H] Da.

(E)-4-hydroxy-N′-(thiophen-2-ylmethylene)benzohydrazide (**Nfz-3**). This compound was obtained 85.98 % from 4-Hydroxybenzhydrazide and 2-Thiophenecarboxaldehyde. FT-IR (v cm^{-1}): 3251 (N-H), 1632 (C=O), 1596 (C=N). ^{1}H NMR (500 MHz, DMSO) δ 11.64 (s, 1H), 8.64 (s, 1H), 7.79 (d,

J = 8.2 Hz, 2H), 7.64 (d, *J* = 5.0 Hz, 1H), 7.43 (d, *J* = 3.5 Hz, 1H), 7.13 (t, *J* = 4.3 Hz, 1H), 6.86 (d, *J* = 8.3 Hz, 2H). *m/z* calculated: 246.28 Da, found: 247.07 [M+H] Da.

(E)-N′-(furan-2-ylmethylene)-4-hydroxybenzohydrazide (**Nfz-4**). This compound was obtained in 93.89 % from 4-Hydroxybenzhydrazide and 2-Furaldehyde. FT-IR (v cm^{-1}): 3249 (N-H), 1619 (C=O), 1604 (C=N). ^{1}H-NMR (500 MHz, DMSO) δ 11.62 (s, 1H), 10.22 (s, 1H), 8.31 (s, 1H), 7.82 (s, 1H), 7.78 (d, *J* = 8.3 Hz, 2H), 6.88 (dd, *J* = 17.9, 5.8 Hz, 3H), 6.63 (d, *J* = 3.3 Hz, 1H). *m/z* calculated: 230.22 Da, found: 231.12 [M+H] Da.

(E)-4-hydroxy-N′-((1-methyl-1H-pyrrol-2-yl)methylene)benzohydrazide (**Nfz-5**). This compound was obtained in 91.14 % from 4-Hydroxybenzhydrazide and N-Methyl-2-pyrrolecarboxaldehyde. FT-IR (v cm^{-1}): 3241 (N-H), 1631 (C=O), 1604 (C=N). ^{1}H-NMR (500 MHz, DMSO) δ 11.31 (s, 1H), 10.07 (s, 1H), 8.36 (s, 1H), 7.77 (d, *J* = 8.2 Hz, 2H), 6.95 (s, 1H), 6.85 (d, *J* = 8.2 Hz, 2H), 6.48 (s, 1H), 6.12 – 6.08 (m, 1H), 3.86 (s, 3H). ^{13}C-NMR (150 MHz, DMSO) δ 162.73, 160.93, 140.63, 129.87, 128.17, 127.74, 124.64, 115.44, 114.41, 108.73, 36.28. *m/z* calculated: 243.26 Da, found: 244.14 [M+H] Da.

(E)-N′-benzylidene-4-hydroxybenzohydrazide (**Nfz-6**). This compound was obtained in 91.46 % from 4-Hydroxybenzhydrazide and benzaldehyde. FT-IR (v cm^{-1}): 3218 (N-H), 1614 (C=O), 1548 (C=N). ^{1}H-NMR (500 MHz, DMSO) δ 11.64 (s, 1H), 10.12 (s, 1H), 8.44 (s, 1H), 7.82 (d, *J* = 8.2 Hz, 2H), 7.70 (dd, *J* = 13.3, 7.7 Hz, 2H), 7.45 (m, *J* = 8.7 Hz, 3H), 6.87 (d, *J* = 8.3 Hz, 1H), 6.78 (d, *J* = 8.3 Hz, 1H). *m/z* calculated: 240.26 Da, found: 241.69 [M+H] Da.

(E)-4-hydroxy-N′-(4-methylbenzylidene)benzohydrazide (**Nfz-7**). This compound was obtained in 93.31 % from 4-Hydroxybenzhydrazide and *p*-Tolualdehyde. FT-IR (v cm^{-1}): 3206 (N-H), 1621 (C=O), 1603 (C=N). ^{1}H NMR (500 MHz, DMSO) δ 11.58 (s, 1H), 10.12 (s, 1H), 8.40 (s, 1H), 7.81 (d,

J = 8.2 Hz, 2H), 7.61 (d, J = 7.7 Hz, 2H), 7.27 (d, J = 7.7 Hz, 2H), 6.87 (d, J = 8.2 Hz, 2H), 2.35 (s, 3H). m/z calculated: 254.28 Da, found: 255.14 [M+H] Da.

(E)-N′-(4-ethylbenzylidene)-4-hydroxybenzohydrazide (**Nfz-8**). This compound was obtained in 94.33 % from 4-Hydroxybenzhydrazide and 4-Ethylbenzaldehyde. FT-IR (v cm^{-1}): 3238 (N-H), 1641 (C=O), 1606 (C=N). 1H NMR (500 MHz, DMSO) δ 11.59 (s, 1H), 10.12 (s, 1H), 8.41 (s, 1H), 7.81 (d, J = 8.4 Hz, 2H), 7.63 (d, J = 7.7 Hz, 2H), 7.30 (d, J = 7.8 Hz, 2H), 6.87 (d, J = 8.4 Hz, 2H), 2.64 (q, J = 7.6 Hz, 2H), 1.20 (t, J = 7.6 Hz, 3H). m/z calculated: 268.31 Da, found: 269.14 [M+H] Da.

(E)-4-hydroxy-N′-(4-hydroxybenzylidene)benzohydrazide (**Nfz-9**). This compound was obtained in 97.40 % from 4-Hydroxybenzhydrazide and 4-Hydroxybenzaldehyde. FT-IR (v cm^{-1}): 3453 (N-H), 1600 (C=O), 1500 (C=N). 1H NMR (500 MHz, DMSO) δ 11.47 (s, 1H), 10.18 (s, 1H), 9.98 (s, 1H), 8.31 (s, 1H), 7.79 (d, J = 8.2 Hz, 2H), 7.55 (d, J = 8.1 Hz, 2H), 6.85 (dd, J = 10.7, 8.2 Hz, 4H). m/z calculated: 256.26 Da, found: 257.11 [M+H] Da.

(E)-4-hydroxy-N′-(2-hydroxybenzylidene)benzohydrazide (**Nfz-10**). This compound was obtained in 96.06 % from 4-Hydroxybenzhydrazide and Salicylaldehyde. FT-IR (v cm^{-1}): 3318 (N-H), 1638 (C=O), 1585 (C=N). ^{1}H-NMR (500 MHz, DMSO) δ 11.94 (s, 1H), 11.43 (s, 1H), 10.28 (s, 1H), 8.60 (s, 1H), 7.83 (d, J = 8.2 Hz, 2H), 7.51 (d, J = 7.6 Hz, 1H), 7.29 (t, J = 7.8 Hz, 1H), 6.96 – 6.86 (m, 3H). m/z calculated: 256.26 Da, found: 257.11 [M+H] Da.

(E)-4-hydroxy-N′-(4-methoxybenzylidene)benzohydrazide (**Nfz-11**). This compound was obtained in 96.17 % from 4-Hydroxybenzhydrazide and 4-Methoxybenzaldehyde. FT-IR (v cm^{-1}): 2930 (CH sp^2), 1660 (C=O), 1624 (C=N), 1061 (C-O-C). ^{1}H-NMR (500 MHz, DMSO) δ 11.53 (s, 1H), 10.19 (s, 1H), 8.36 (s, 1H), 7.79 (dd, J = 8.7, 1.8 Hz, 2H), 7.67 (d, J = 8.3 Hz, 2H), 7.04 – 6.99 (m, 2H), 6.89 – 6.83 (m, 2H), 3.80 (d, J = 2.0 Hz, 3H). m/z calculated: 270.28 Da, found: 271.11 [M+H] Da.

(E)-4-hydroxy-N′-(2-methoxybenzylidene)benzohydrazide (**Nfz-12**). This compound was obtained in 96.63 % from 4-Hydroxybenzhydrazide and 2-Methoxybenzaldehyde. FT-IR (v cm^{-1}): 3087 (N-H), 1601 (C=O), 1553 (C=N). ^{1}H-NMR (500 MHz, DMSO) δ 11.76 (s, 1H), 10.15 (s, 1H), 8.60 (s, 1H), 8.13 (s, 1H), 8.01 (dd, J = 6.2, 3.3 Hz, 1H), 7.85 (d, J = 8.4 Hz, 2H), 7.57 (dd, J = 6.3, 3.2 Hz, 2H), 6.89 (d, J = 8.4 Hz, 2H), 2.51 (t, J = 2.1 Hz, 3H). m/z calculated: 270.28 Da, found: 271.65 [M+H] Da.

(E)-N′-(2,3-dimethoxybenzylidene)-4-hydroxybenzohydrazide (**Nfz-13**). This compound was obtained in 92.01 % from 4-Hydroxybenzhydrazide and 2,3-dimethoxybenzaldehyde. FT-IR (v cm^{-1}): 3157 (N-H), 1603 (C=O), 1573 (C=N), 1061 (C-O-C). ^{1}H-NMR (500 MHz, DMSO) δ 11.68 (s, 1H), 10.12 (s, 1H), 8.72 (s, 1H), 7.83 (d, J = 8.2 Hz, 2H), 7.47 (d, J = 7.5 Hz, 1H), 7.17 – 7.08 (m, 2H), 6.87 (d, J = 8.2 Hz, 2H), 3.84 (s, 3H), 3.80 (s, 3H). ^{13}C-NMR (150 MHz, DMSO) δ 163.11, 161.16, 153.14, 148.36, 142.86, 130.12, 129.26, 128.44, 124.81, 124.26, 117.45, 115.47, 115.28, 114.49, 61.68, 56.22. m/z calculated: 300.31 Da, found: 301.08 [M+H] Da.

(E)-4-hydroxy-N′-(3,4,5-trimethoxybenzylidene)benzohydrazide (**Nfz-14**). This compound was obtained in 91.80 % from 4-Hydroxybenzhydrazide and 3,4,5-Trimethoxybenzaldehyde. FT-IR (v cm^{-1}): 3277 (N-H), 1640 (C=O), 1605 (C=N). ^{1}H-NMR (500 MHz, DMSO) δ 11.66 (s, 1H), 10.21 (s, 1H), 8.34 (s, 1H), 7.79 (d, 2H), 7.03 (s, 2H), 6.86 (d, 2H), 3.83 (s, 6H), 3.70 (s, 3H). ^{13}C-NMR (150 MHz, DMSO) δ 163.28, 161.14, 153.65, 147.37, 139.53, 130.52, 130.12, 124.36, 115.49, 104.65, 60.59, 56.41. m/z calculated: 330.34 Da, found: 331.03 [M+H] Da.

(E)-N′-(4-fluorobenzylidene)-4-hydroxybenzohydrazide (**Nfz-15**). This compound was obtained in 95.61 % from 4-Hydroxybenzhydrazide and 4-Fluorobenzaldehyde. FT-IR (v cm^{-1}): 3242 (N-H), 1626 (C=O), 1597 (C=N). ^{1}H-NMR (500 MHz, DMSO) δ 11.68 (s, 1H), 10.23 (s, 1H), 8.41 (s, 1H), 7.81-7.77 (m, 3H), 7.29 (t, J = 8.6 Hz, 3H), 6.87 (d, J = 8.1 Hz, 2H). m/z calculated: 258.25 Da, found: 259.08 [M+H] Da.

(E)-N′-(4-chlorobenzylidene)-4-hydroxybenzohydrazide (**Nfz-16**). This compound was obtained in 94.48 % from 4-Hydroxybenzhydrazide and 4-Chlorobenzaldehyde. FT-IR (*v* cm^{-1}): 3281 (N-H), 1604 (C=O), 1575 (C=N). ^{1}H-NMR (500 MHz, DMSO) δ 11.71 (s, 1H), 10.14 (s, 1H), 8.43 (s, 1H), 7.81 (d, *J* = 8.3 Hz, 2H), 7.74 (d, *J* = 8.1 Hz, 2H), 7.52 (d, *J* = 8.1 Hz, 2H), 6.87 (d, *J* = 8.3 Hz, 2H). *m*/*z* calculated: 274.70 Da, found: 275.08 [M+H] Da.

(E)-N′-(4-bromobenzylidene)-4-hydroxybenzohydrazide (**Nfz-17**). This compound was obtained in 96.20 % from 4-Hydroxybenzhydrazide and 4-Bromobenzaldehyde. FT-IR (*v* cm^{-1}): 3287 (N-H), 1620 (C=O), 1604 (C=N). ^{1}H-NMR (500 MHz, DMSO) δ 11.73 (s, 1H), 10.23 (s, 1H), 8.39 (s, 1H), 7.80 (d, *J* = 8.2 Hz, 2H), 7.66 (q, *J* = 8.3 Hz, 4H), 6.90–6.84 (m, 2H). *m*/*z* calculated: 319.15 Da, found: 318.93 [M-H], 320.94 [M+H] Da.

(E)-4-hydroxy-N′-(4-nitrobenzylidene)benzohydrazide (**Nfz-18**). This compound was obtained in 87.99 % from 4-Hydroxybenzhydrazide and 4-Nitrobenzaldehyde. FT-IR (*v* cm^{-1}): 3338 (N-H), 1655 (C=O), 1604 (C=N). ^{1}H-NMR (500 MHz, DMSO) δ 11.96 (s, 1H), 10.28 (s, 1H), 8.51 (s, 1H), 8.29 (d, *J* = 8.3 Hz, 2H), 7.97 (d, *J* = 8.3 Hz, 2H), 7.82 (d, *J* = 8.2 Hz, 2H), 6.91–6.86 (m, 2H). *m*/*z* calculated: 285.25 Da, found: 286.04 [M+H] Da.

(E)-4-hydroxy-N′-(2-nitrobenzylidene)benzohydrazide (**Nfz-19**). This compound was obtained in 93.78 % from 4-Hydroxybenzhydrazide and 2-Nitrobenzaldehyde. FT-IR (*v* cm^{-1}): 3222 (N-H), 1639 (C=O), 1605 (C=N). ^{1}H-NMR (500 MHz, DMSO) δ 12.02 (s, 1H), 10.27 (s, 1H), 8.83 (s, 1H), 8.06 (d, *J* = 8.1 Hz, 1H), 7.82 (dd, *J* = 17.5, 8.2 Hz, 3H), 7.66 (t, *J* = 7.8 Hz, 2H), 6.88 (d, *J* = 8.2 Hz, 2H). *m*/*z* calculated: 285.25 Da, found: 286.04 [M+H] Da.

(E)-4-hydroxy-N′-(naphthalen-2-ylmethylene)benzohydrazide (**Nfz-20**). This compound was obtained in 80.97 % from 4-Hydroxybenzhydrazide and 2-Naphthaldehyde. FT-IR (*v* cm^{-1}): 3324 (N-H), 1649 (C=O), 1602 (C=N). ^{1}H-NMR (500 MHz, DMSO) δ 11.76 (s, 1H), 10.15 (s, 1H), 8.60 (s, 1H),

8.13 (s, 1H), 8.05 – 7.92 (m, 4H), 7.85 (d, *J* = 8.4 Hz, 2H), 7.57 (dd, *J* = 6.3, 3.2 Hz, 2H), 6.89 (d, *J* = 8.4 Hz, 2H). *m/z* calculated: 290.32 Da, found: 291.07 [M+H] Da.

(E)-N′-((2,3-dihydrobenzo[b][1,4]dioxin-6-yl)methylene)-4-hydroxybenzohydrazide (**Nfz-21**). This compound was obtained in 79.38 % from 4-Hydroxybenzhydrazide and 1,4-Benzodioxan-6-carboxaldehyde. FT-IR (*v* cm^{-1}): 3337 (N-H), 1645 (C=O), 1604 (C=N). ^{1}H-NMR (500 MHz, DMSO) δ 11.53 (s, 1H), 10.10 (s, 1H), 8.31 (s, 1H), 7.79 (d, *J* = 8.2 Hz, 2H), 7.23 – 7.16 (m, 2H), 6.93 (d, *J* = 8.2 Hz, 1H), 6.86 (d, *J* = 8.2 Hz, 2H), 4.29 (s, 4H). ^{13}C-NMR (150 MHz, DMSO) δ 165.56, 162.59, 147.00, 145.57, 144.07, 130.05, 128.40, 124.40, 121.09, 117.92, 115.56, 115.47, 64.75, 64.49. *m/z* calculated: 298.29 Da, found: 299.06 [M+H] Da.

(E)-N′-([1,1′-biphenyl]-4-ylmethylene)-4-hydroxybenzohydrazide (**Nfz-22**). This compound was obtained in 91.71 % from 4-Hydroxybenzhydrazide and Biphenyl-4-carboxaldehyde. FT-IR (*v* cm^{-1}): 3273 (N-H), 1645 (C=O), 1605 (C=N). ^{1}H-NMR (500 MHz, DMSO) δ 11.76 (s, 1H), 10.15 (s, 1H), 8.60 (s, 1H), 8.13 (s, 2H), 8.03-7.94 (m, 4H), 7.85 (d, *J* = 8.4 Hz, 2H), 7.57 (dd, *J* = 6.3, 3.2 Hz, 3H), 6.89 (d, *J* = 8.4 Hz, 2H). ^{13}C-NMR (150 MHz, DMSO) δ 146.87, 141.82, 139.83, 134.11, 130.15, 129.50, 128.32, 128.03, 127.51, 127.12, 115.51. *m/z* calculated: 316.35 Da, found: 317.65 [M+H] Da.

(E)-4-hydroxy-N′-((6-nitrobenzo[d][1,3]dioxol-5-yl)methylene)benzohydrazide (**Nfz-23**). This compound was obtained in 93.90 % from 4-Hydroxybenzhydrazide and 6-Nitropiperonal. FT-IR (*v* cm^{-1}): 3270 (N-H), 1640 (C=O), 1601 (C=N). ^{1}H-NMR (500 MHz, DMSO) δ 11.96 (s, 1H), 10.17 (s, 1H), 8.85 (s, 1H), 7.83 (d, *J* = 8.3 Hz, 2H), 7.68 (s, 1H), 7.48 (s, 1H), 6.87 (d, *J* = 8.3 Hz, 2H), 6.30 (s, 2H). ^{13}C-NMR (150 MHz, DMSO) δ 161.38, 152.25, 149.24, 143.48, 142.49, 130.28, 126.47, 123.90, 115.51, 105.59, 105.45, 104.26. *m/z* calculated: 329.26 Da, found: 330.01 [M+H] Da.

(E)-N-(4-((2-(4-hydroxybenzoyl)hydrazono)methyl)phenyl)acetamide (**Nfz-24**). This compound was obtained in 89.50 % from 4-Hydroxybenzhydrazide and 4-Acetamidobenzaldehyde.

FT-IR (v cm^{-1}): 3325 (N-H), 3252 (N-H), 1675 (C=O), 1649 (C=O), 1589 (C=N). ^{1}H-NMR (500 MHz, DMSO) δ 11.58 (s, 1H), 10.15 (s, 1H), 8.35 (s, 1H), 7.80 (d, J = 8.2 Hz, 2H), 7.66 (s, 4H), 6.86 (d, J = 8.2 Hz, 2H), 2.07 (s, 3H). m/z calculated: 297.31 Da, found: 298.64 [M+H] Da.

(E)-methyl 4-((2-(4-hydroxybenzoyl)hydrazono)methyl)benzoate (**Nfz-25**). This compound was obtained in 85.08 % from 4-Hydroxybenzhydrazide and Methyl 4-formylbenzoate. FT-IR (v cm^{-1}): 3292 (N-H), 1692 (C=O), 1659 (C=O), 1609 (C=N). ^{1}H-NMR (500 MHz, DMSO) δ 11.83 (s, 1H), 10.19 (d, J = 14.5 Hz, 1H), 8.48 (s, 1H), 8.02 (d, J = 7.9 Hz, 2H), 7.88–7.80 (m, 4H), 6.88 (d, J = 8.2 Hz, 2H), 3.87 (s, 3H). ^{13}C-NMR (150 MHz, DMSO) δ 166.34, 161.31, 145.89, 139.47, 130.70, 130.25, 130.12, 127.55, 124.10, 115.52, 52.71. m/z calculated: 298.29 Da, found: 299.08 [M+H] Da.

Spectrums of Nfz

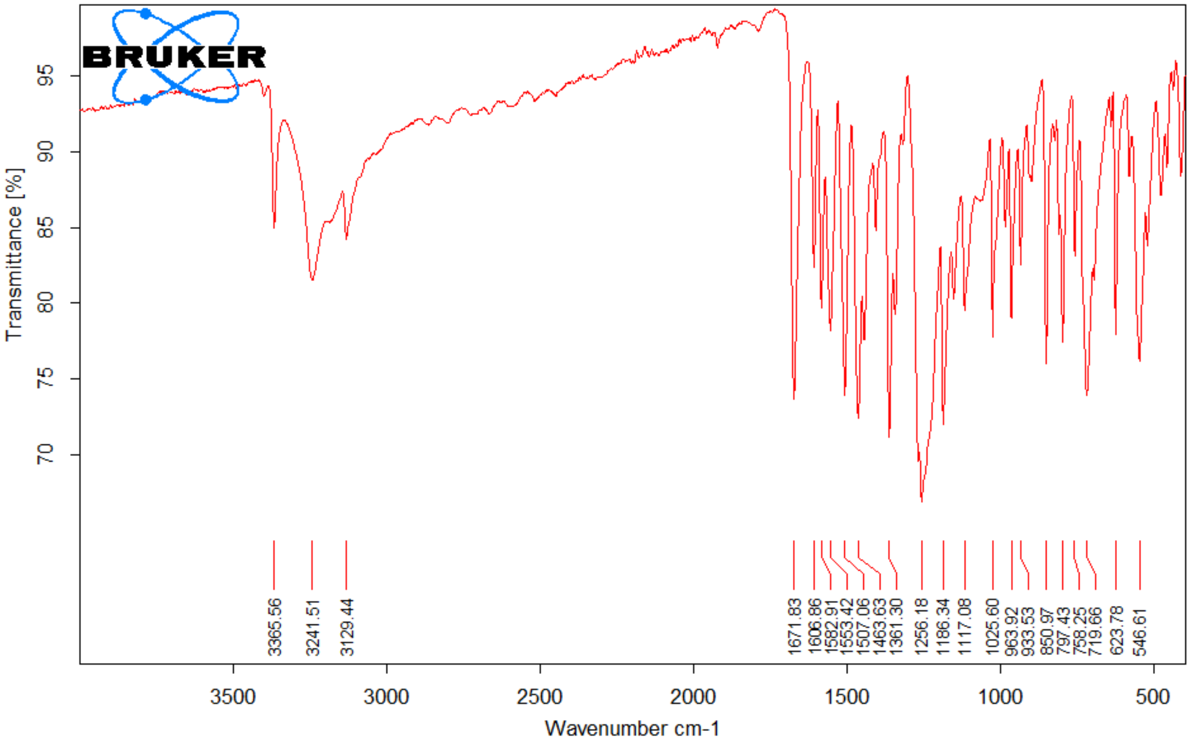

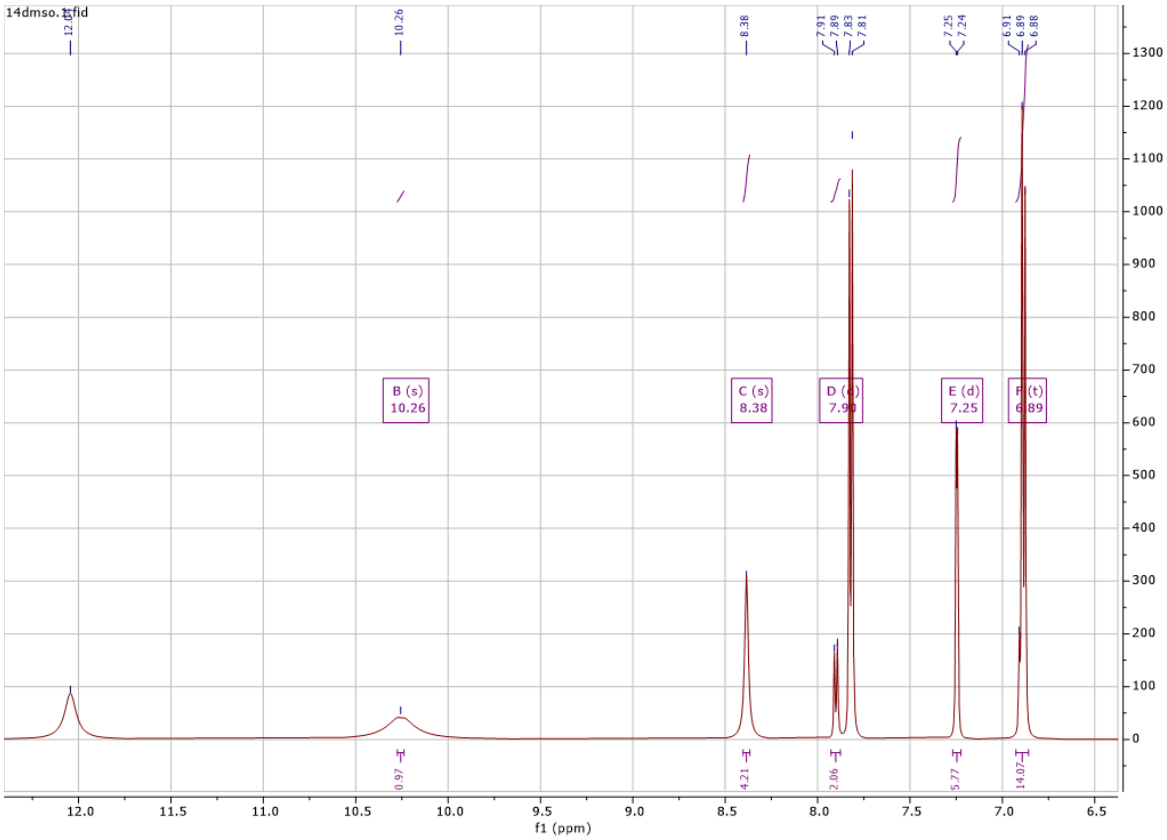

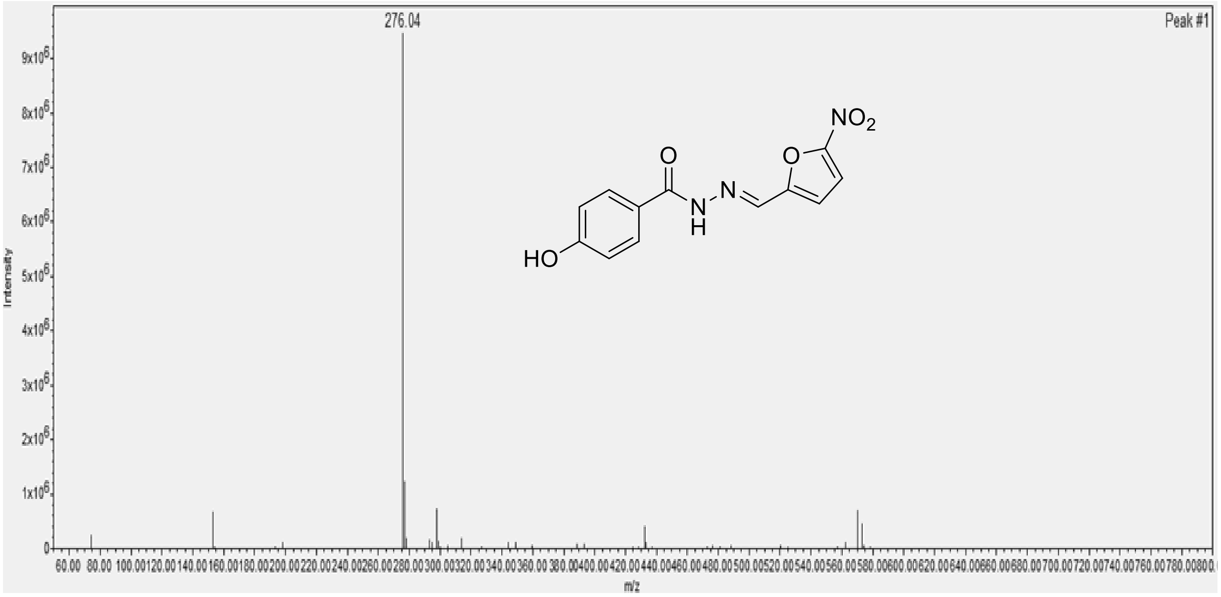

Spectrums of **Nfz-1**

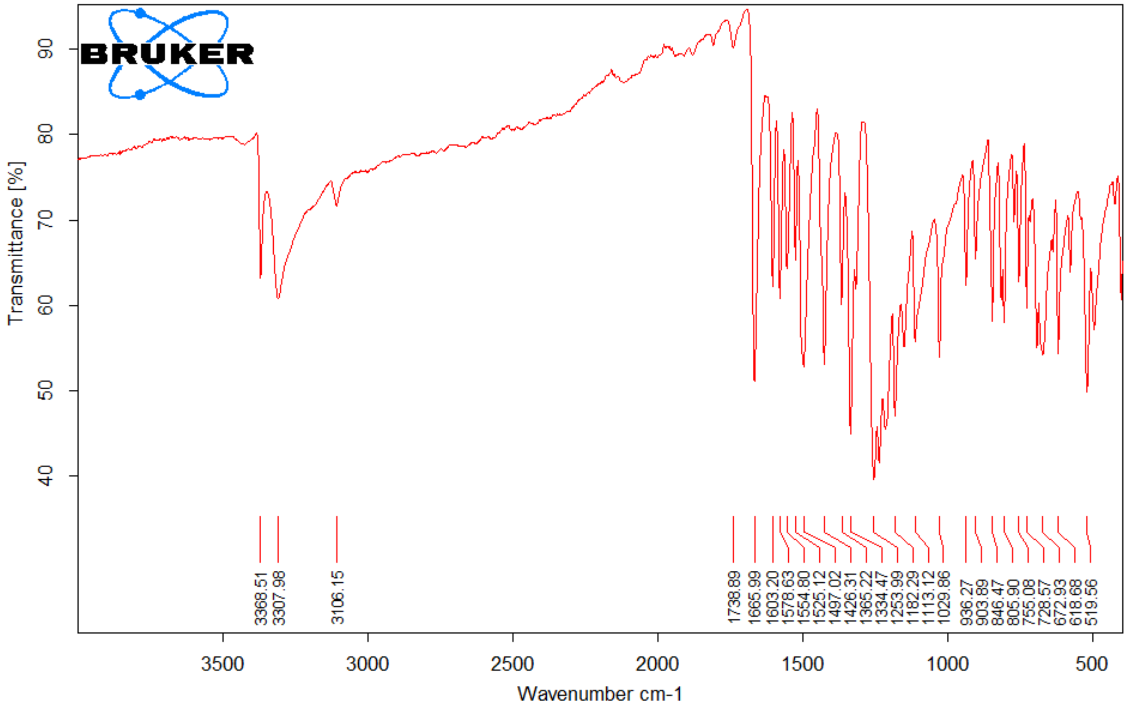
BRUKER
Transmittance [%]
Wavenumber cm-1
3368.51
3307.98
3106.15
1738.89
1665.99
1603.20
1578.63
1554.80
1525.12
1497.02
1426.31
1365.22
1334.47
1253.99
1182.29
1113.12
1029.86
936.27
903.89
846.47
805.90
755.08
728.57
672.93
618.68
519.56

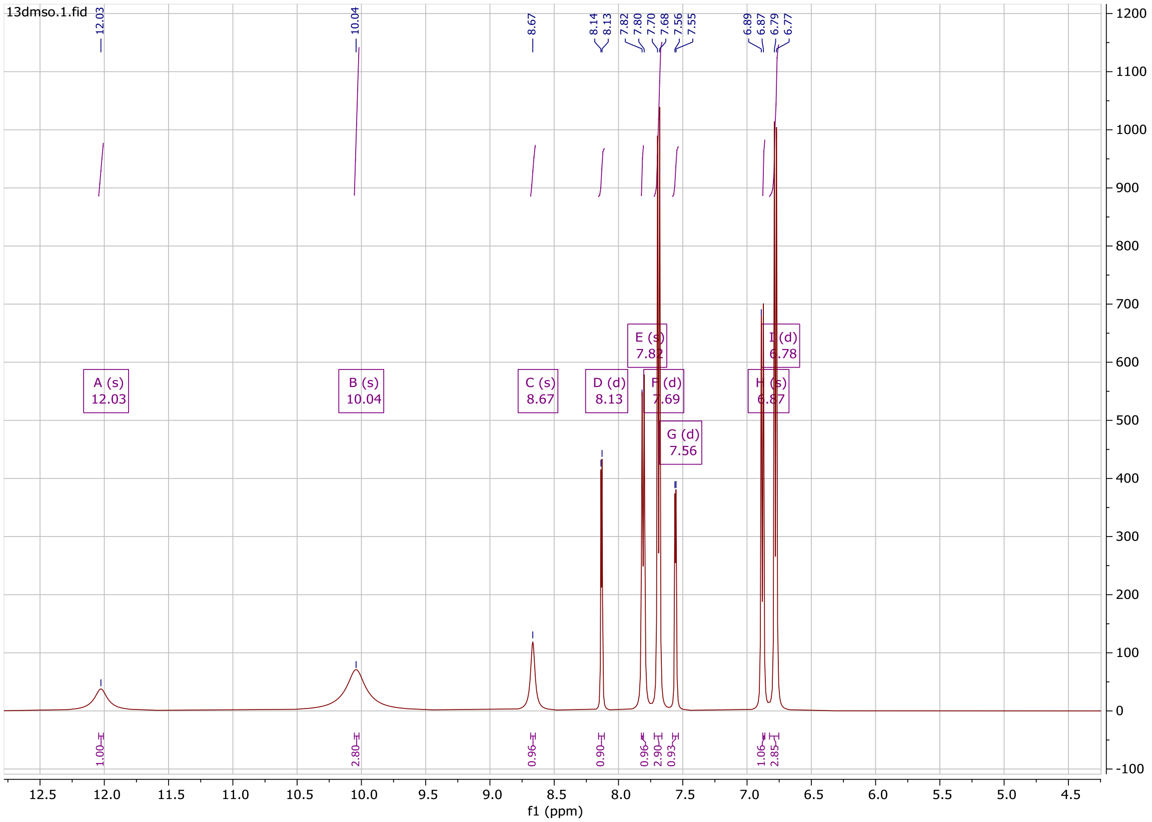
13dmso.1.fid
A (s) 12.03
B (s) 10.04
C (s) 8.67
D (d) 8.13
E (s) 7.82
F (d) 7.69
G (d) 7.56
H (s) 6.87
I (d) 6.78
f1 (ppm)

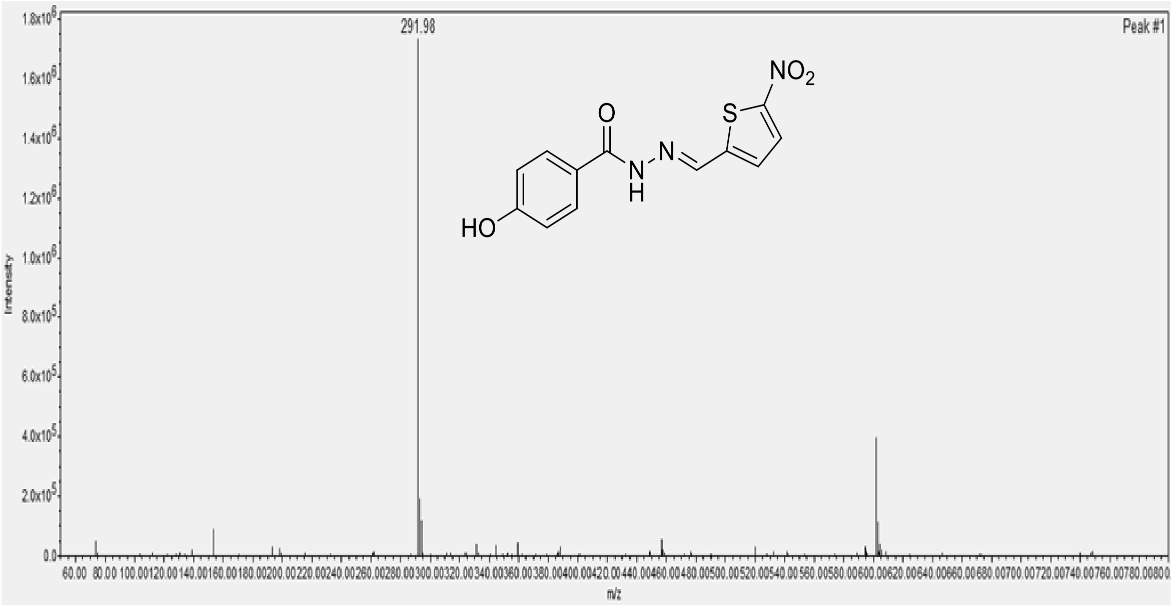

Spectrums of **Nfz-2**

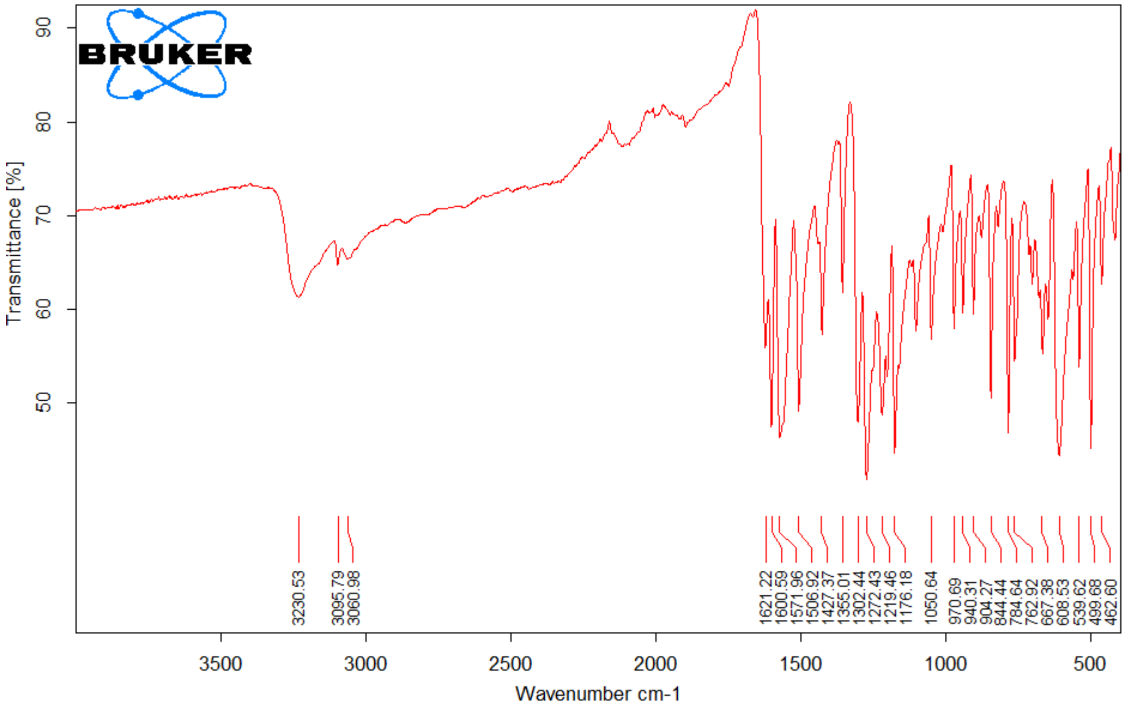

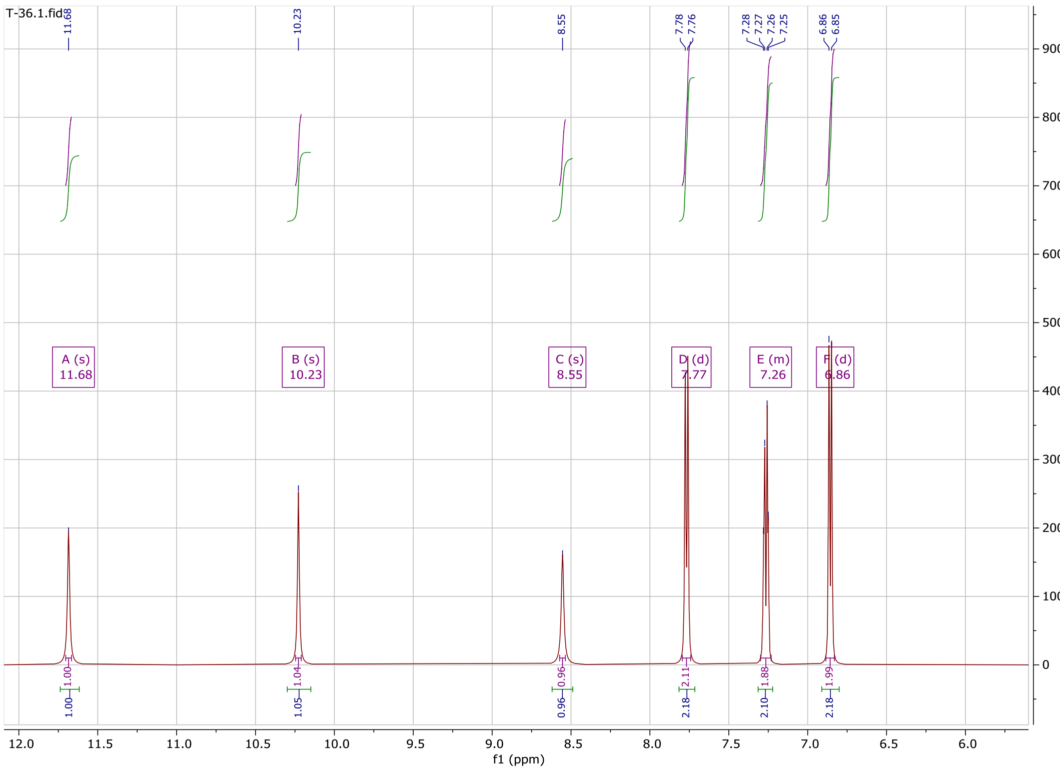

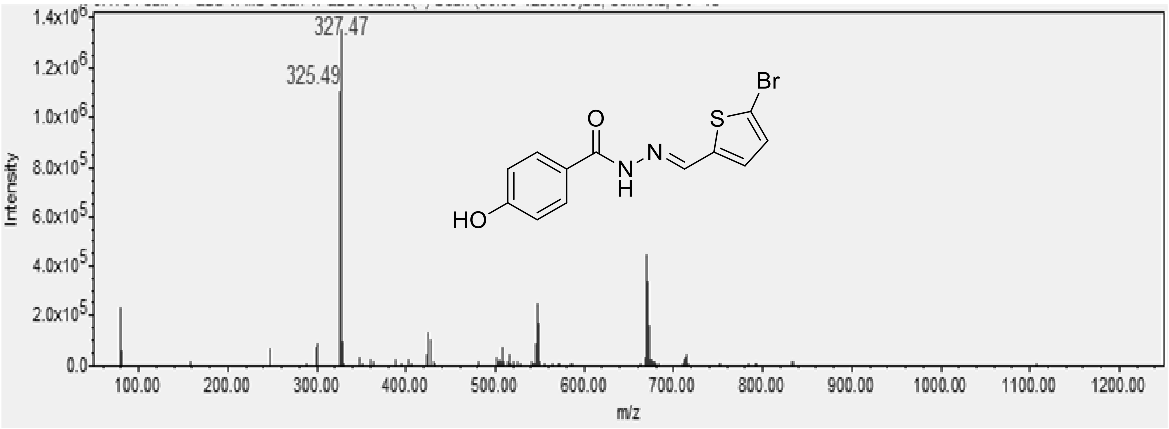

Spectrums of **Nfz-3**

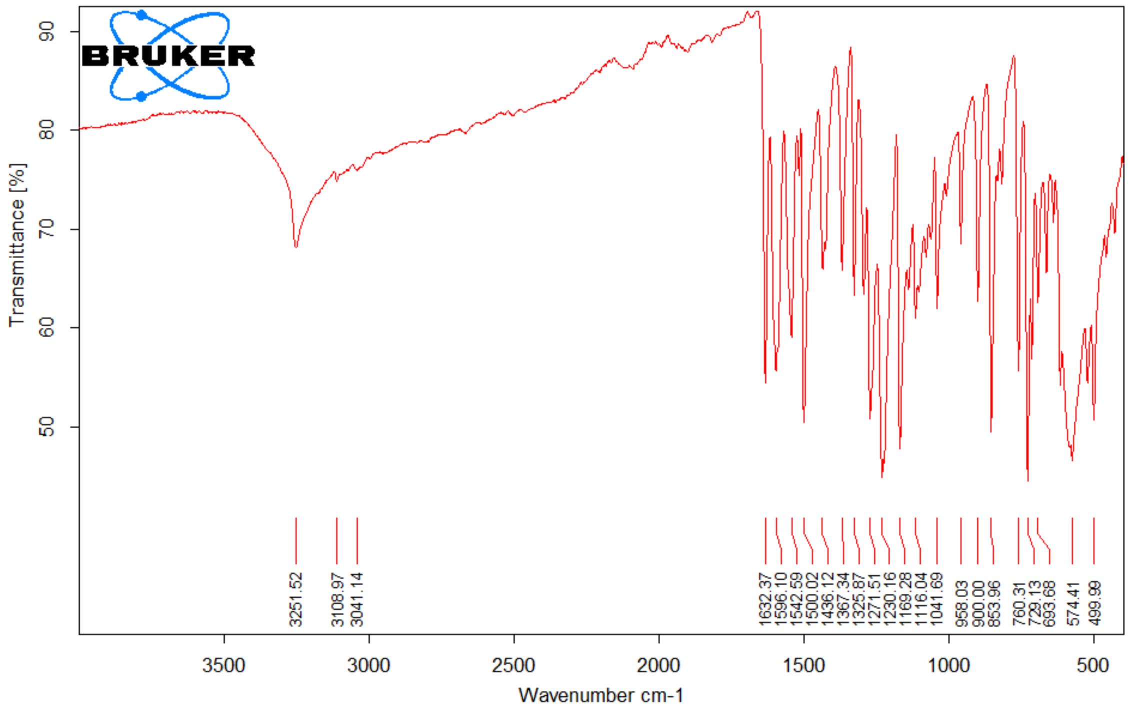
BRUKER
Transmittance [%]
Wavenumber cm-1
3251.52
3108.97
3041.14
1632.37
1596.10
1542.59
1500.02
1436.12
1367.34
1325.87
1271.51
1230.16
1169.28
1116.04
1041.69
958.03
900.00
853.96
760.31
729.13
693.68
574.41
499.99

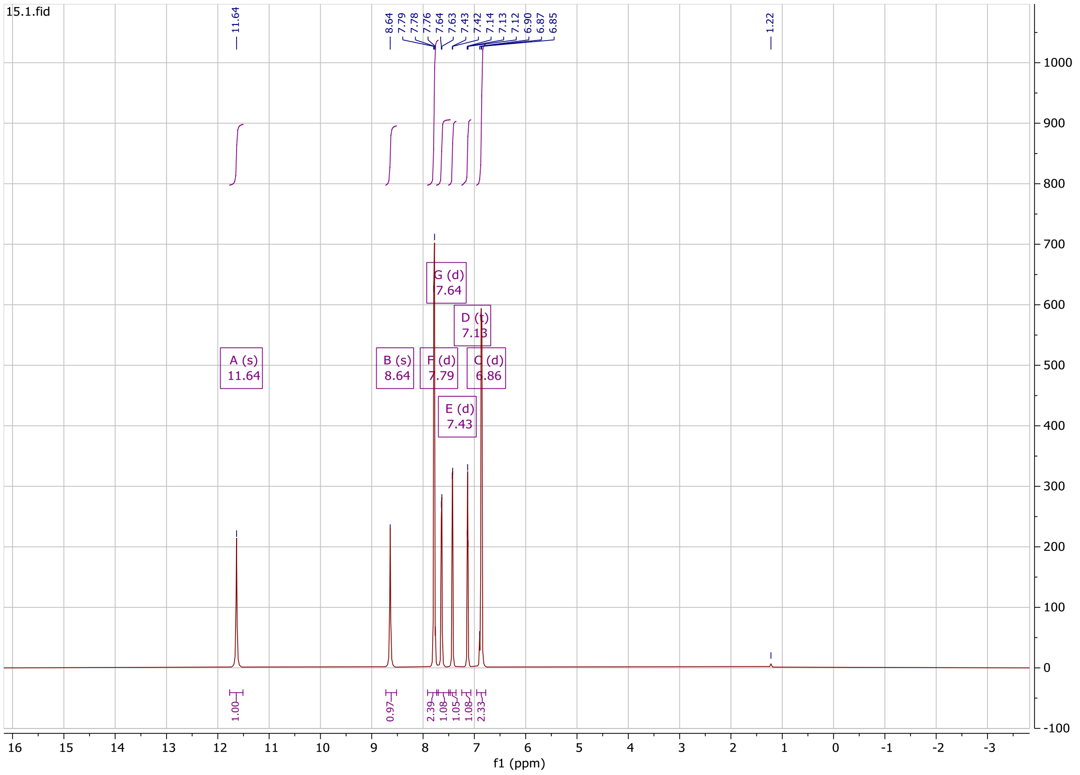
15.1.fid
11.64
8.64 7.79 7.78 7.76 7.64 7.63 7.43 7.42 7.14 7.13 7.12 6.90 6.87 6.85
1.22
A (s) 11.64
B (s) 8.64
F (d) 7.79
G (d) 7.64
E (d) 7.43
D (t) 7.13
C (d) 6.86
1.00
0.97
2.39
1.08
1.05
1.08
2.33
f1 (ppm)

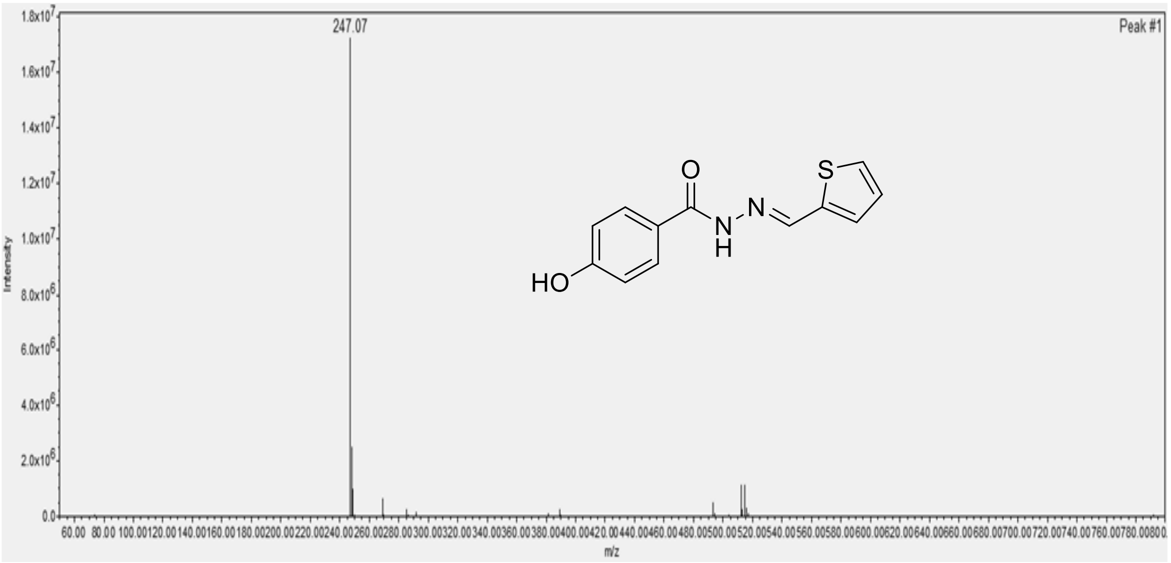

Spectrums of **Nfz-4**

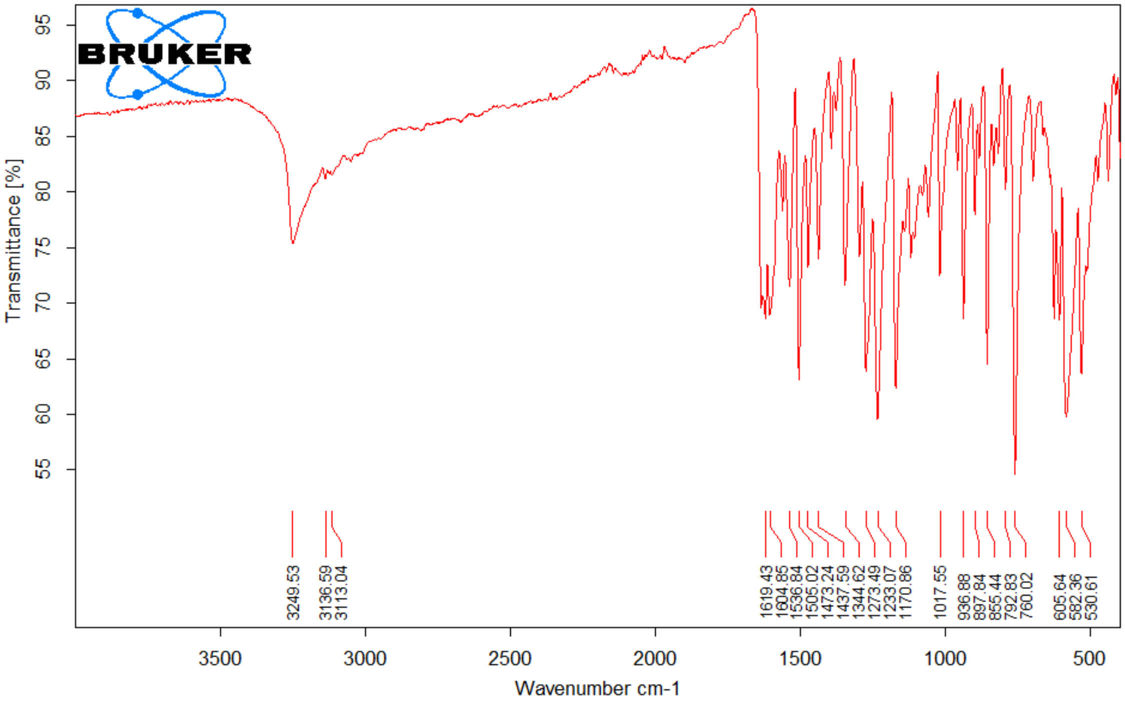

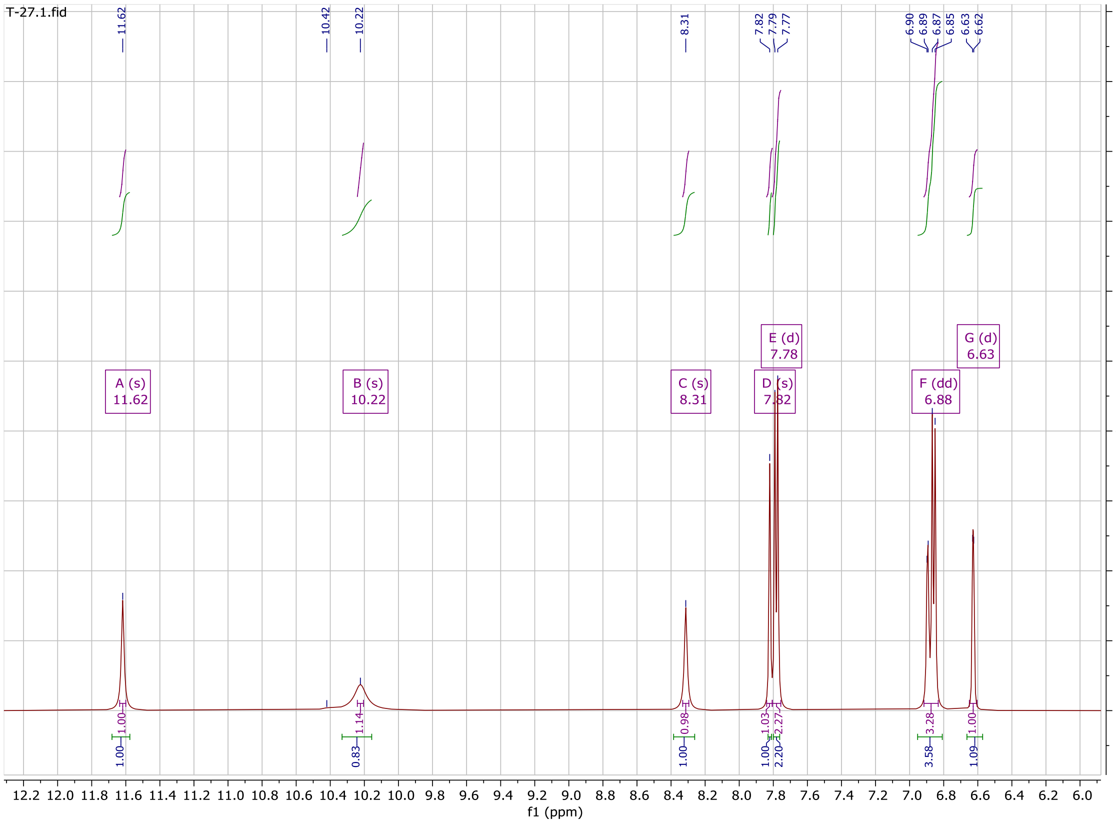

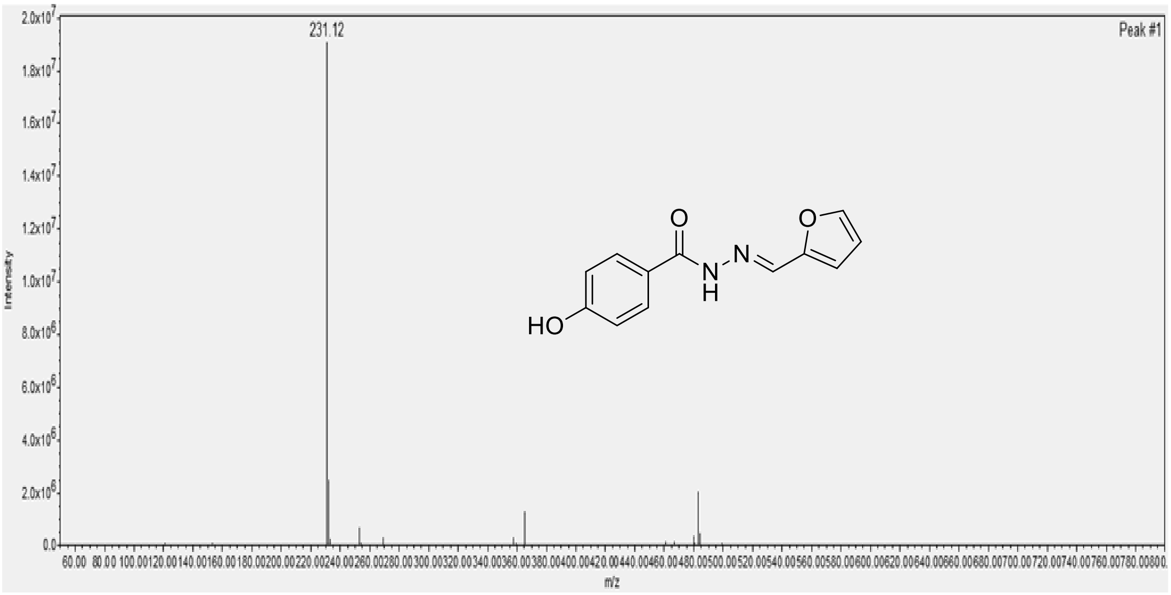

Spectrums of **Nfz-5**

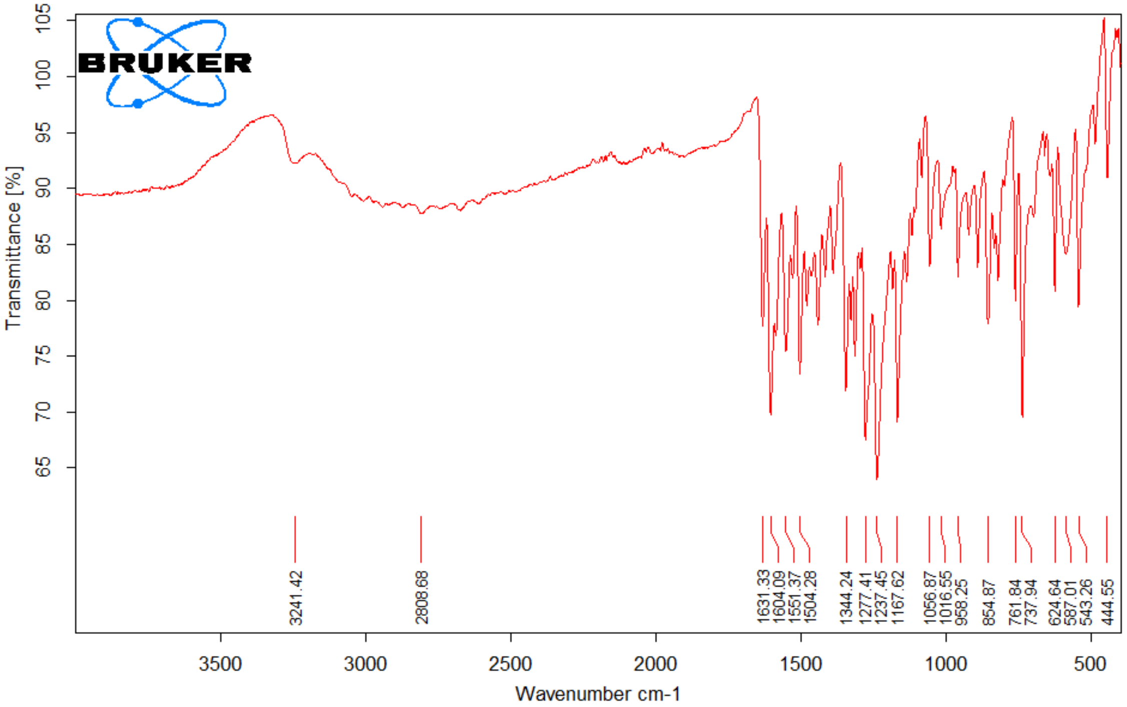

BRUKER
Transmittance [%]
Wavenumber cm-1
3241.42
2808.68
1631.33
1604.09
1551.37
1504.28
1344.24
1277.41
1237.45
1167.62
1056.87
1016.55
958.25
854.87
761.84
737.94
624.64
587.01
543.26
444.55

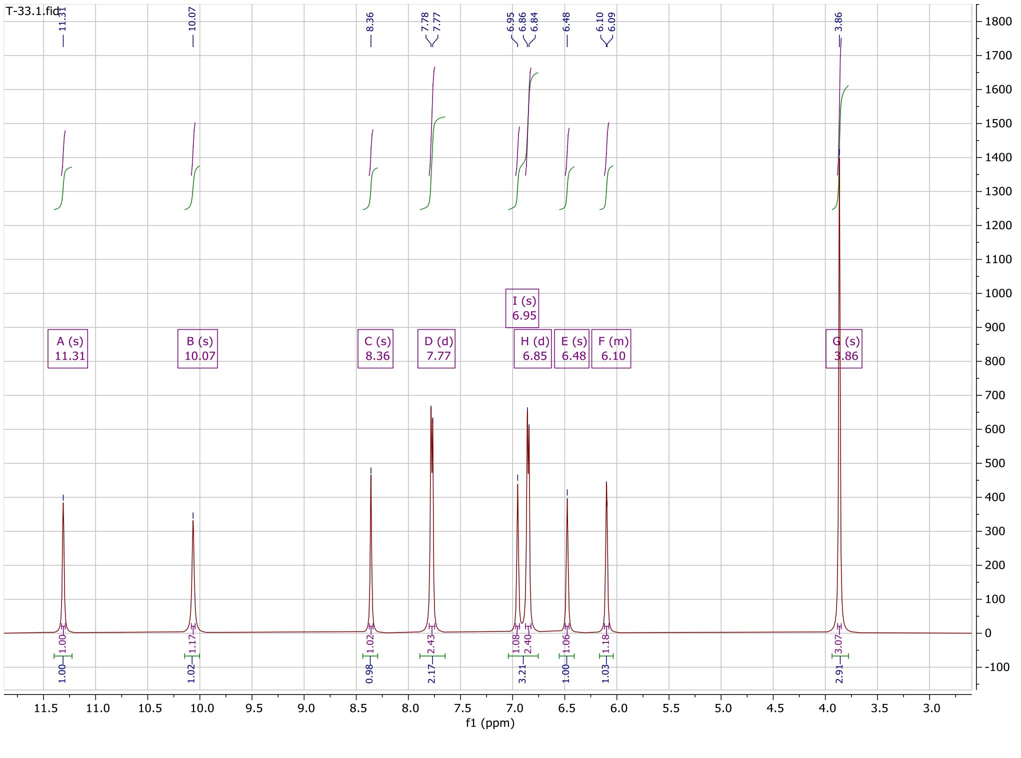

T-33.1.fid
A (s) 11.31
B (s) 10.07
C (s) 8.36
D (d) 7.77
I (s) 6.95
H (d) 6.85
E (s) 6.48
F (m) 6.10
G (s) 3.86
f1 (ppm)

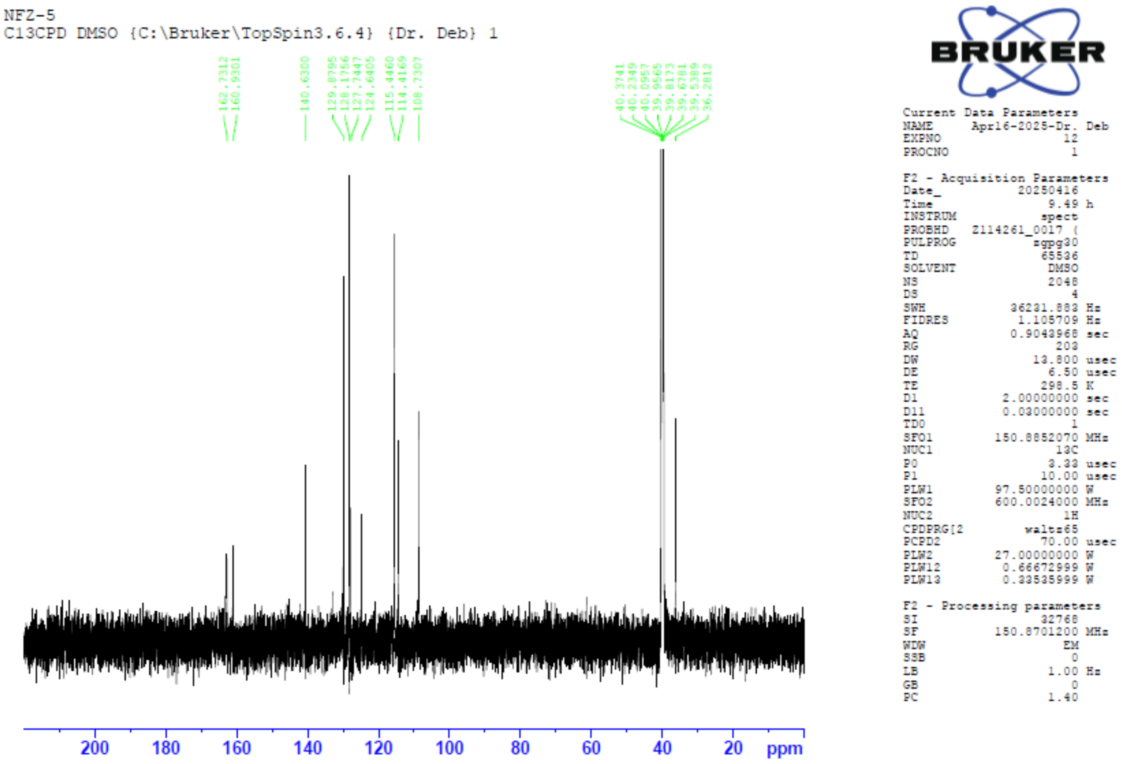

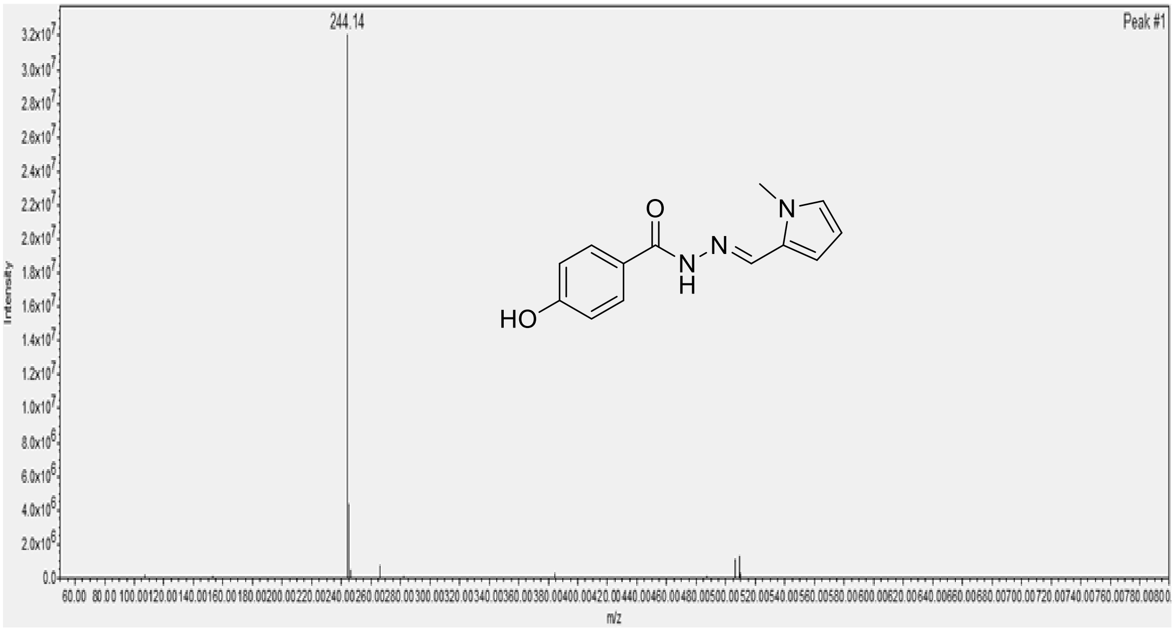

Spectrums of **Nfz-6**

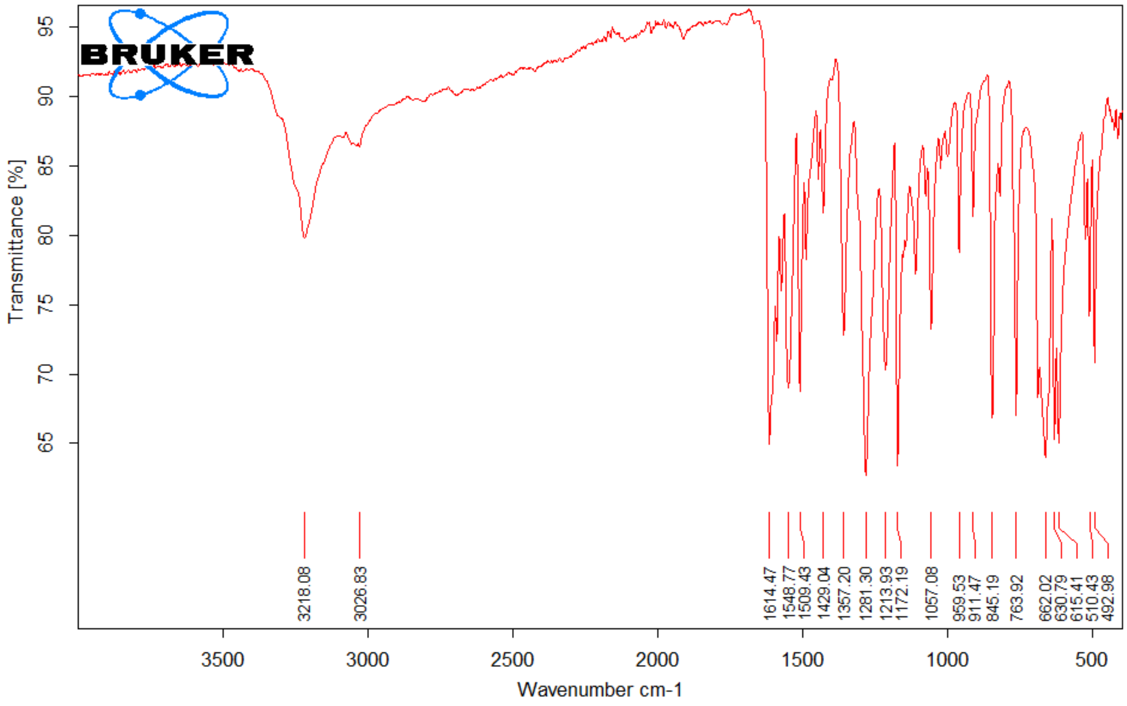
BRUKER
Transmittance [%]
Wavenumber cm-1
3218.08
3026.83
1614.47
1548.77
1509.43
1429.04
1357.20
1281.30
1213.93
1172.19
1057.08
959.53
911.47
845.19
763.92
662.02
630.79
615.41
510.43
492.98

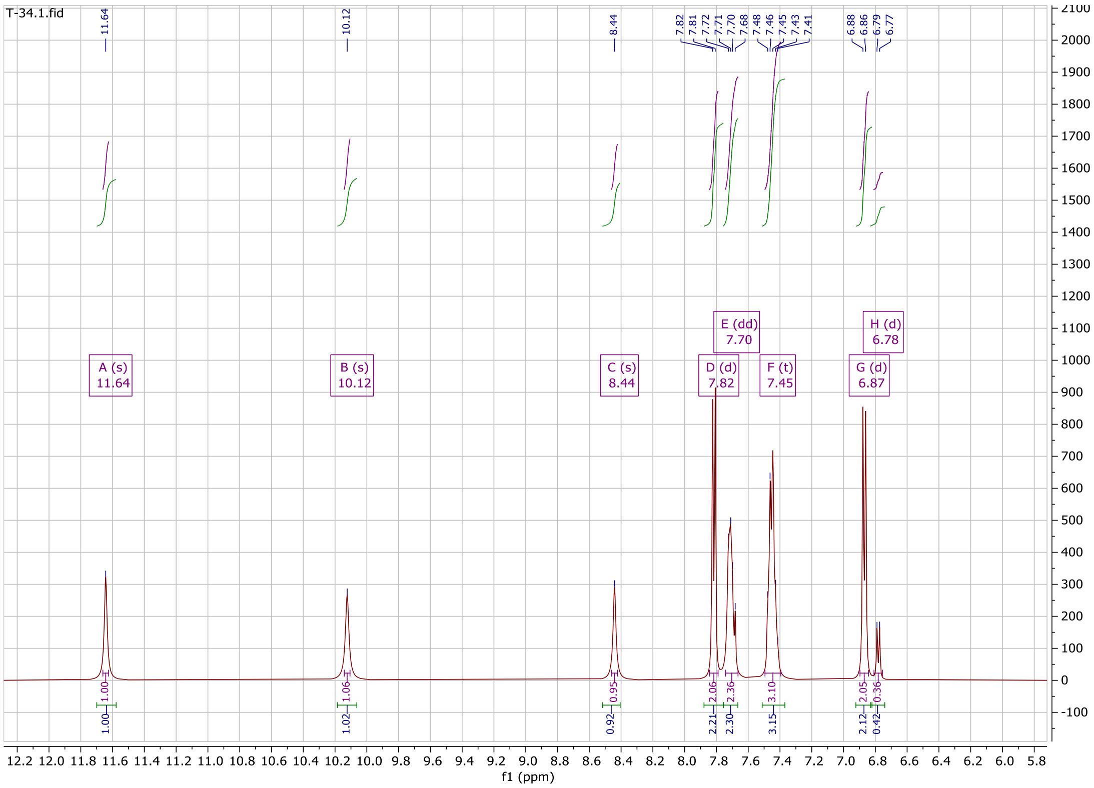
T-34.1.fid
A (s) 11.64
B (s) 10.12
C (s) 8.44
D (d) 7.82
E (dd) 7.70
F (t) 7.45
G (d) 6.87
H (d) 6.78
f1 (ppm)

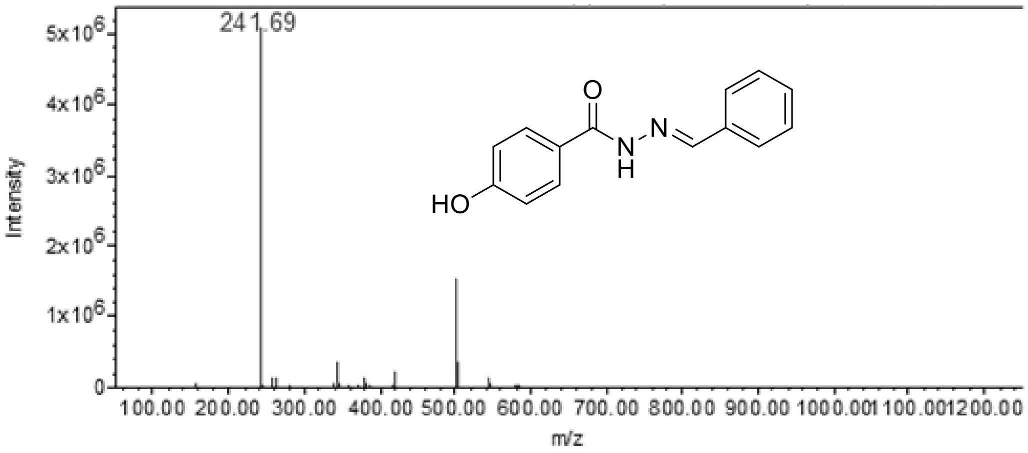

Spectrums of **Nfz-7**

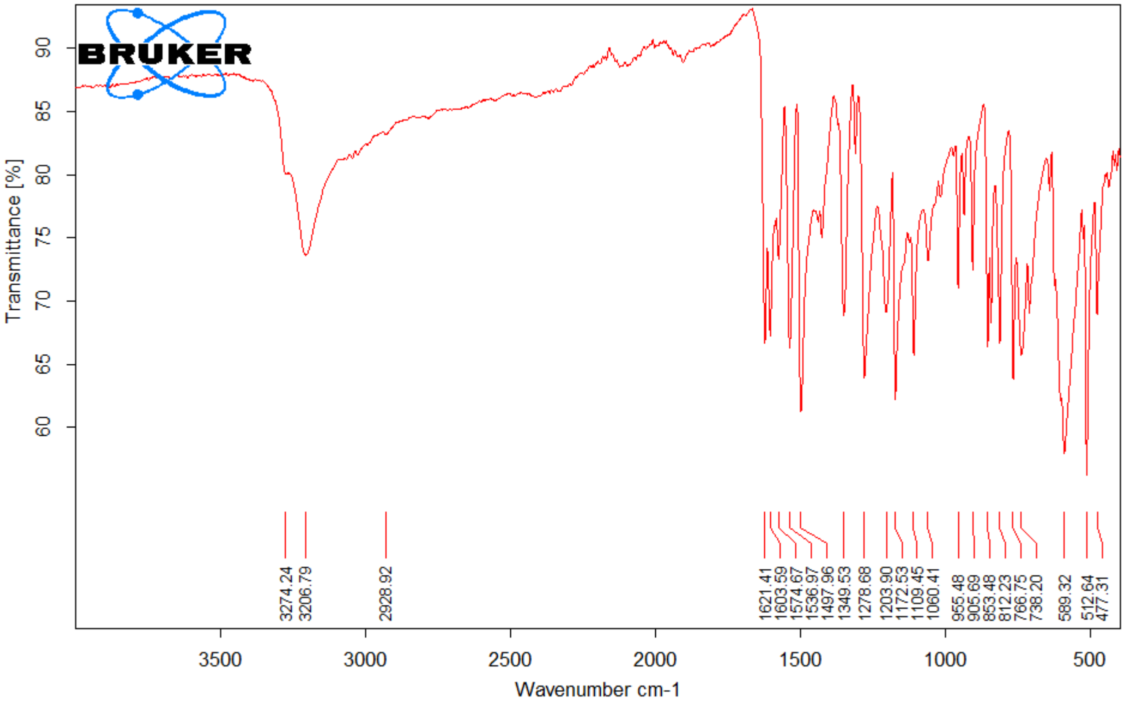

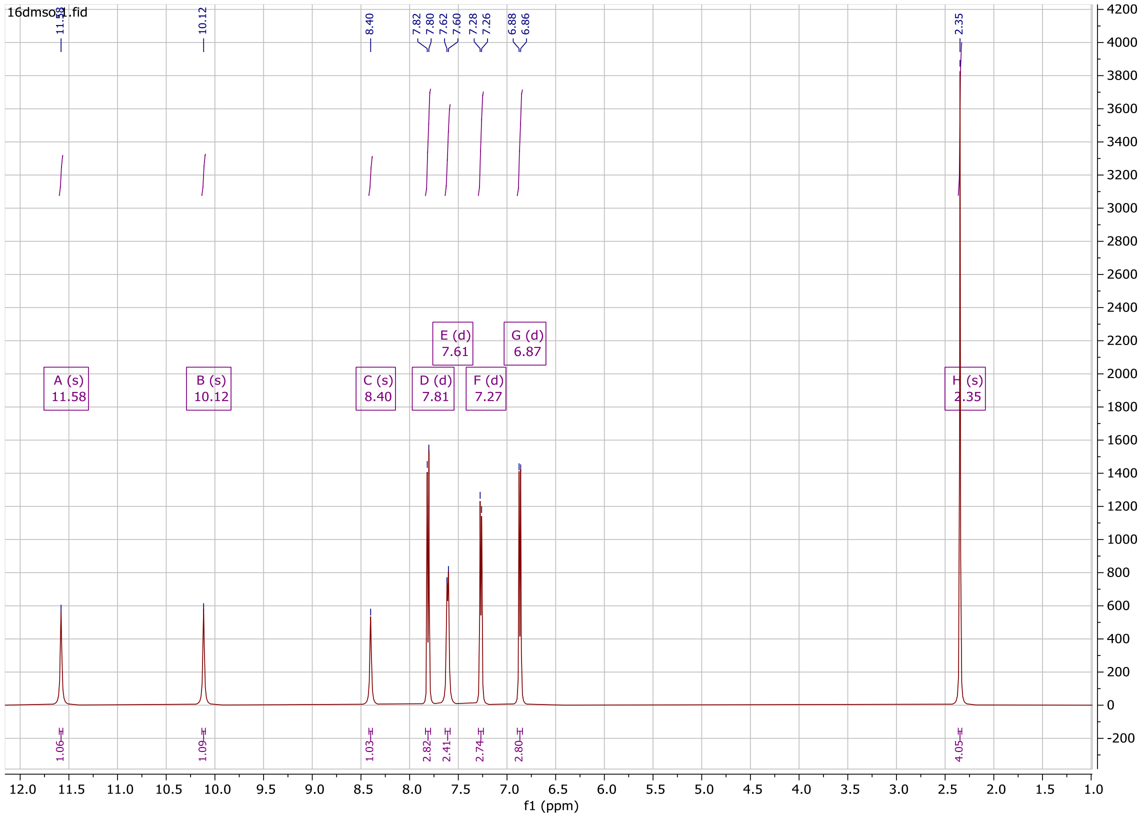

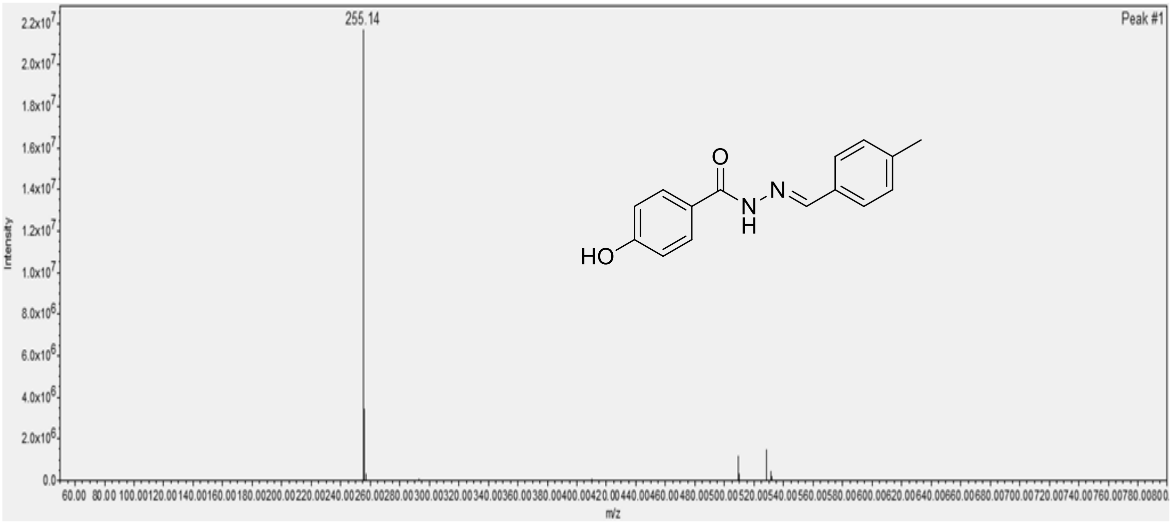

Spectrums of **Nfz-8**

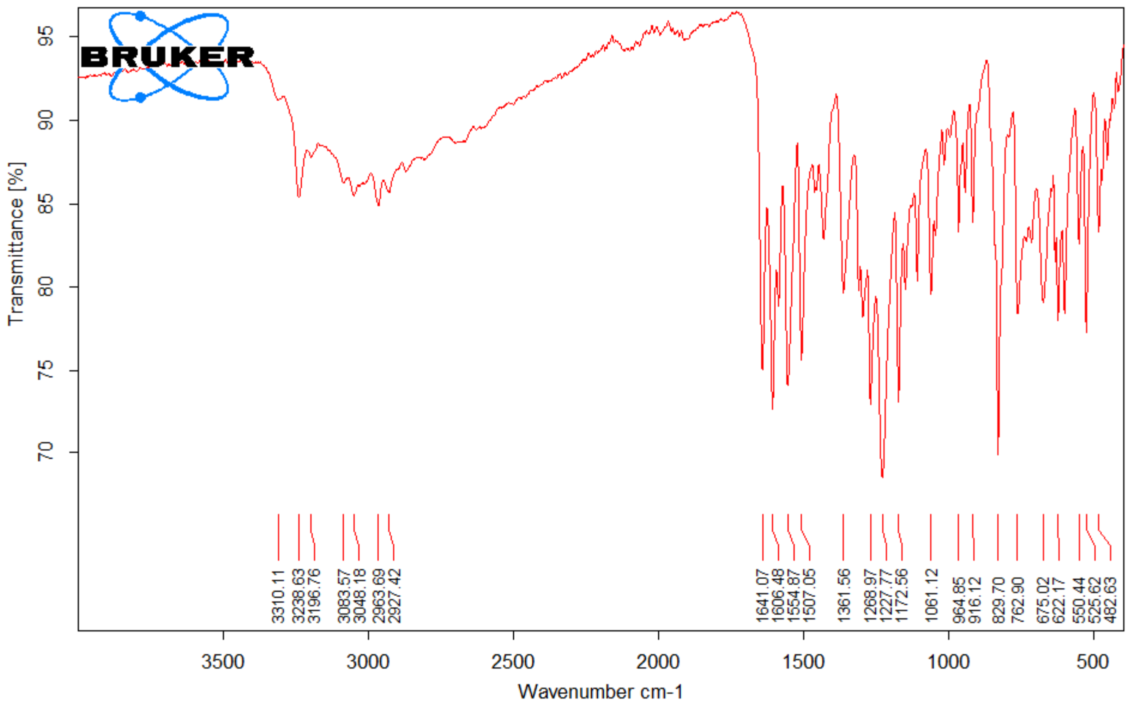
BRUKER
Transmittance [%]
Wavenumber cm-1
3310.11
3238.63
3196.76
3083.57
3048.18
2963.69
2927.42
1641.07
1606.48
1554.87
1507.05
1361.56
1268.97
1227.77
1172.56
1061.12
964.85
916.12
829.70
762.90
675.02
622.17
550.44
525.62
482.63

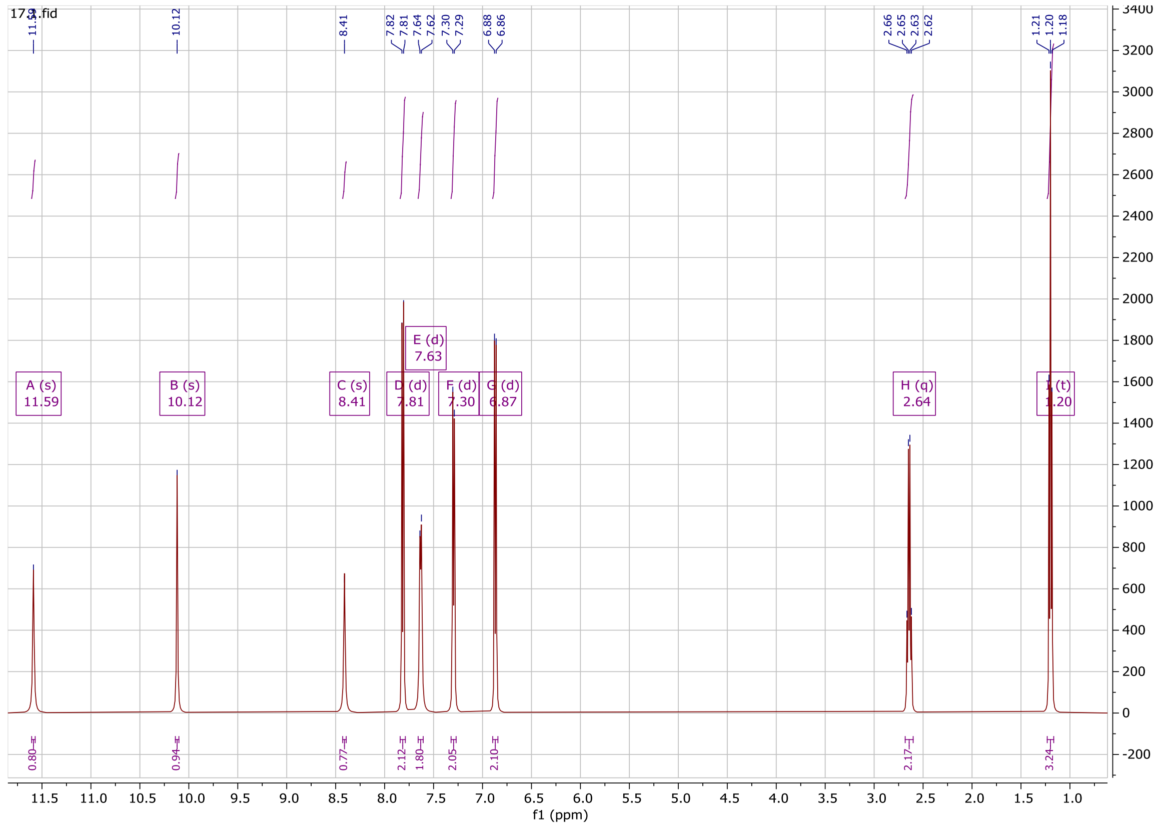
A (s)
11.59
B (s)
10.12
C (s)
8.41
D (d)
7.81
E (d)
7.63
F (d)
7.30
G (d)
6.87
H (q)
2.64
I (t)
1.20
f1 (ppm)

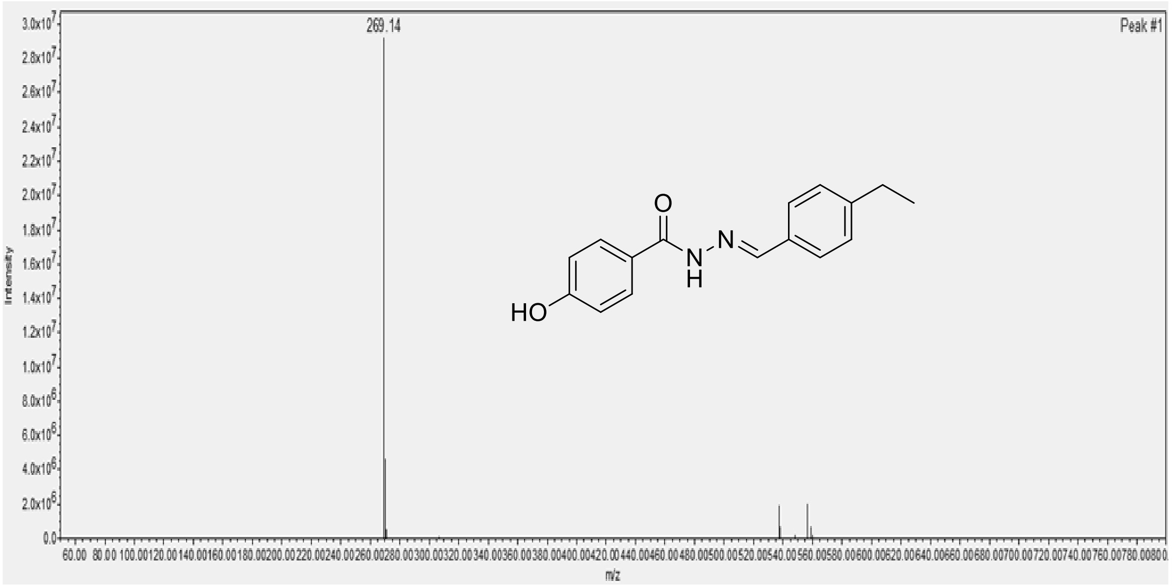

Spectrums of **Nfz-9**

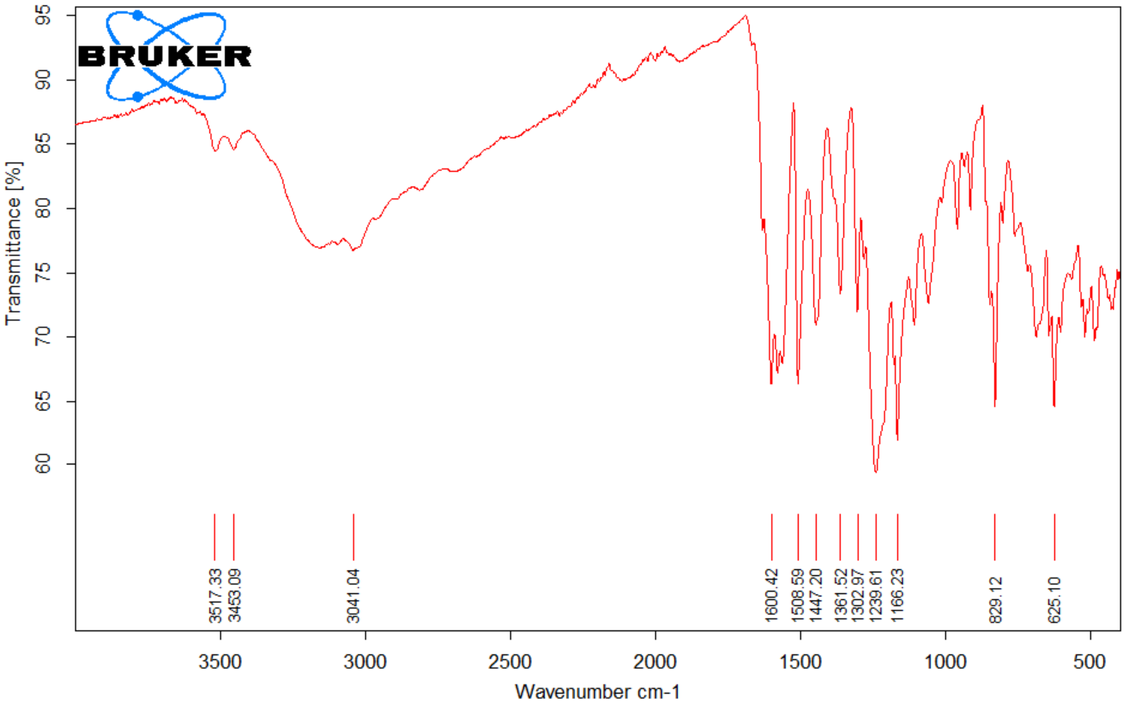

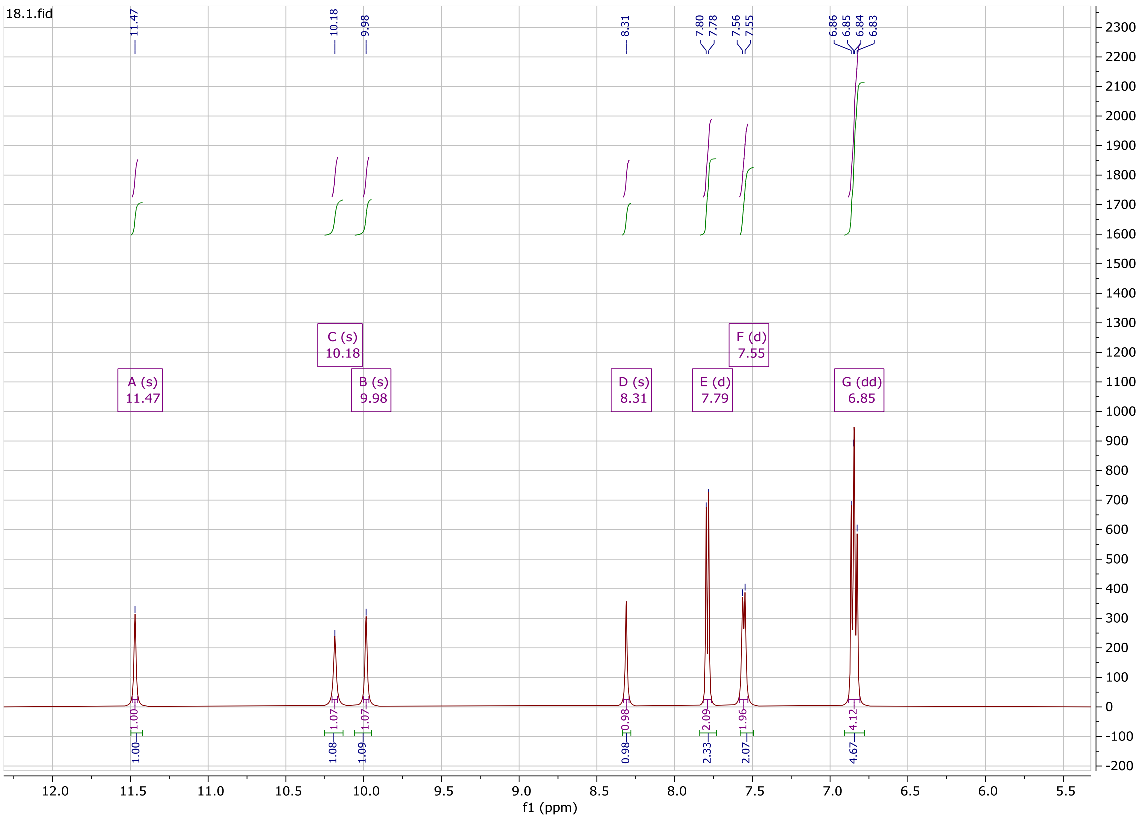

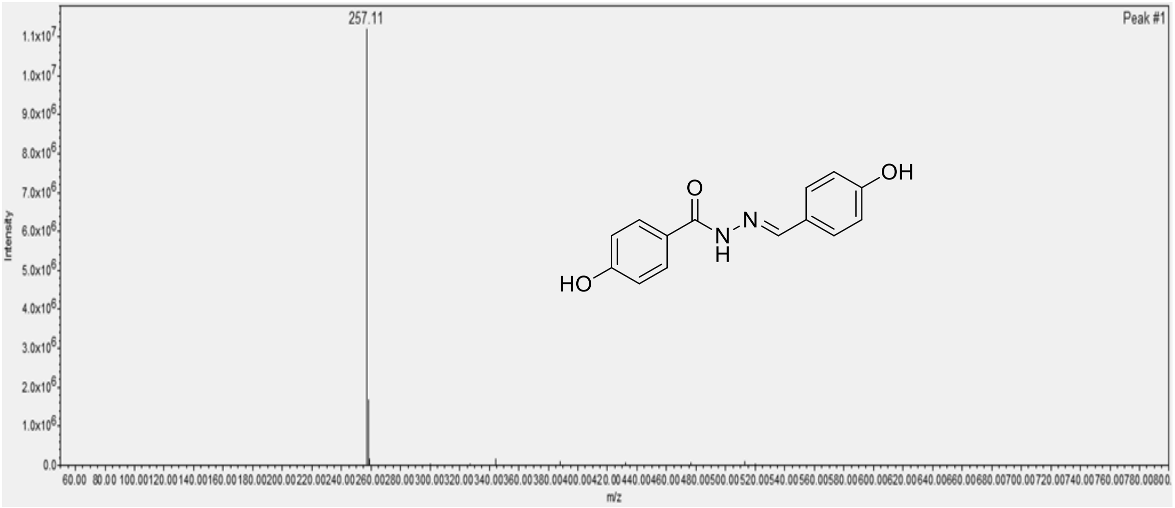

Spectrums of **Nfz-10**

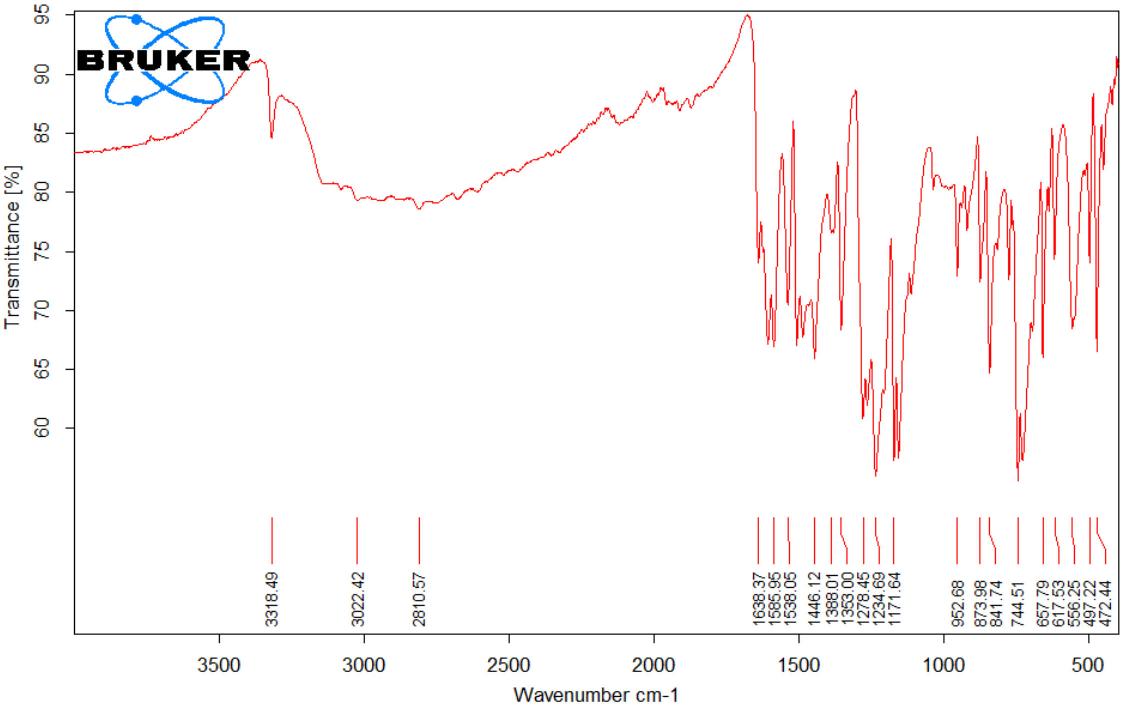
BRUKER
Transmittance [%]
95
90
85
80
75
70
65
60
3318.49
3022.42
2810.57
1638.37
1585.95
1538.05
1446.12
1388.01
1353.00
1278.45
1234.69
1171.64
952.68
873.98
841.74
744.51
657.79
617.53
556.25
497.22
472.44
3500
3000
2500
2000
1500
1000
500
Wavenumber cm-1

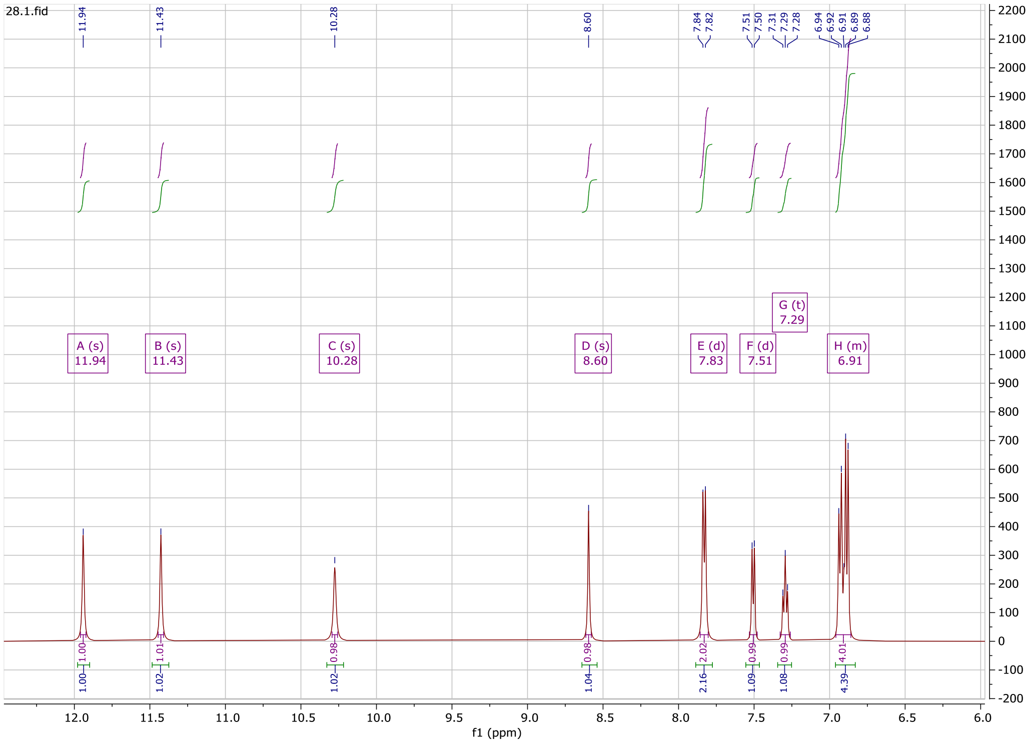
28.1.fid
11.94
11.43
10.28
8.60
7.84
7.82
7.51
7.50
7.31
7.29
7.28
6.94
6.92
6.91
6.89
6.88
A (s)
11.94
B (s)
11.43
C (s)
10.28
D (s)
8.60
E (d)
7.83
F (d)
7.51
G (t)
7.29
H (m)
6.91
1.00
1.01
0.98
0.98
2.02
0.99
0.99
4.01
1.00
1.02
1.02
1.04
2.16
1.09
1.08
4.39
12.0
11.5
11.0
10.5
10.0
9.5
9.0
8.5
8.0
7.5
7.0
6.5
6.0
f1 (ppm)
2200
2100
2000
1900
1800
1700
1600
1500
1400
1300
1200
1100
1000
900
800
700
600
500
400
300
200
100
0
-100
-200

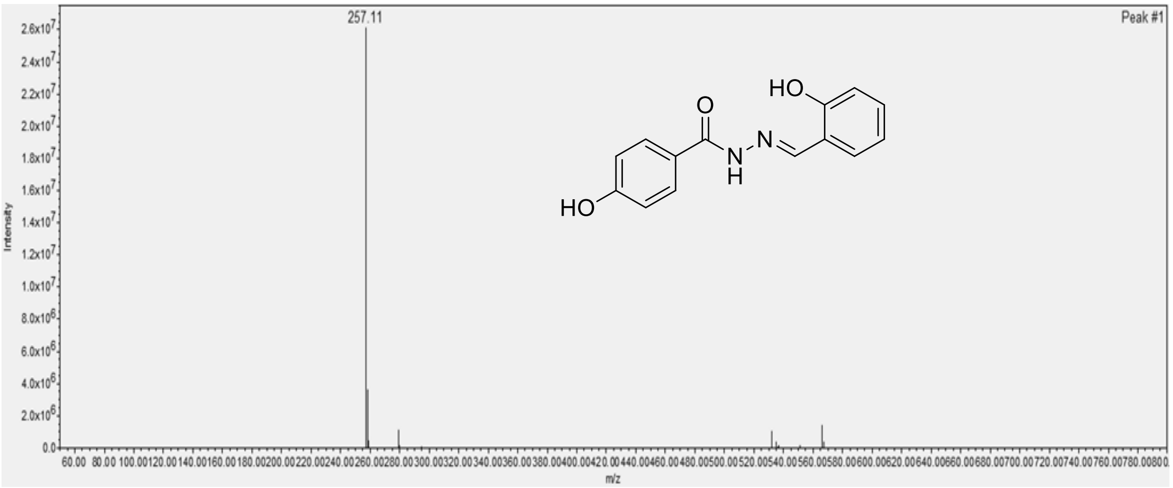

Spectrums of **Nfz-11**

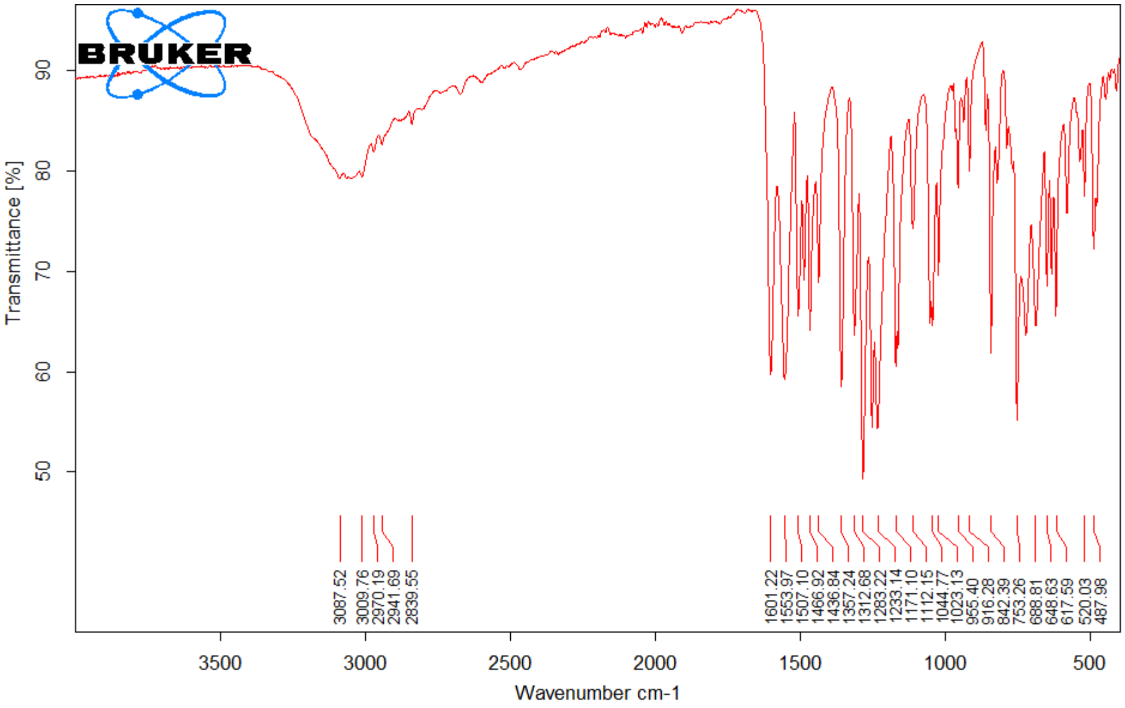

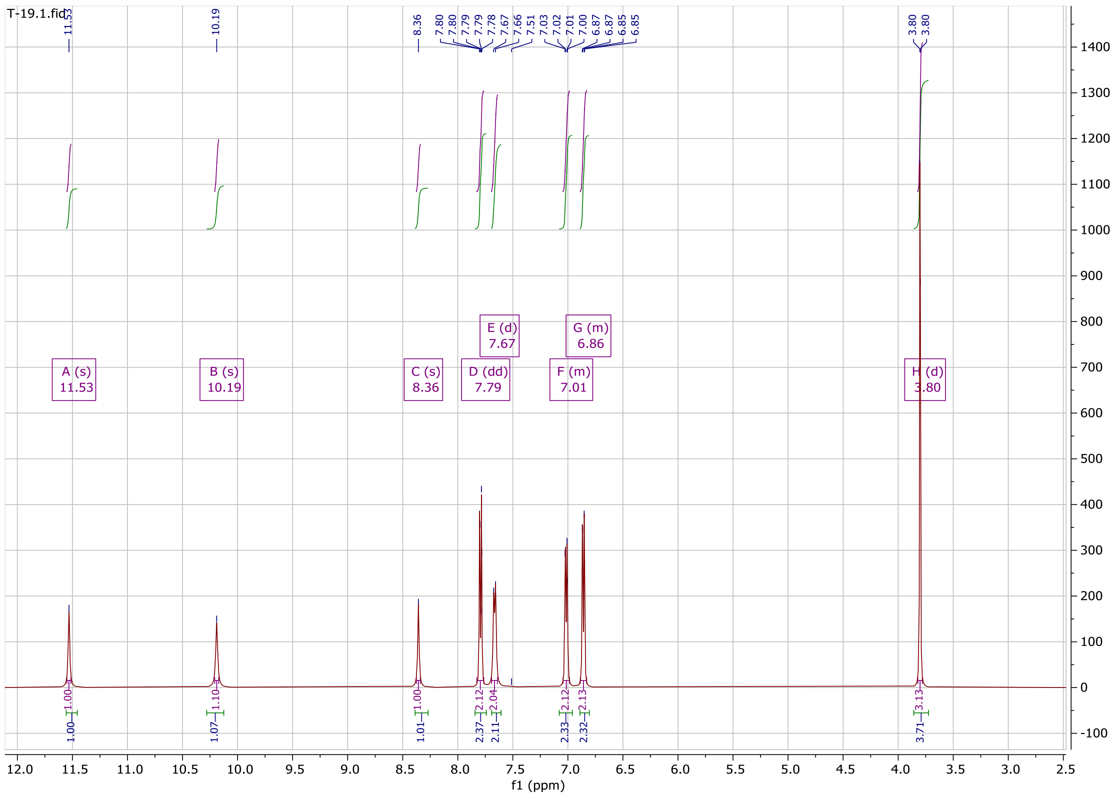

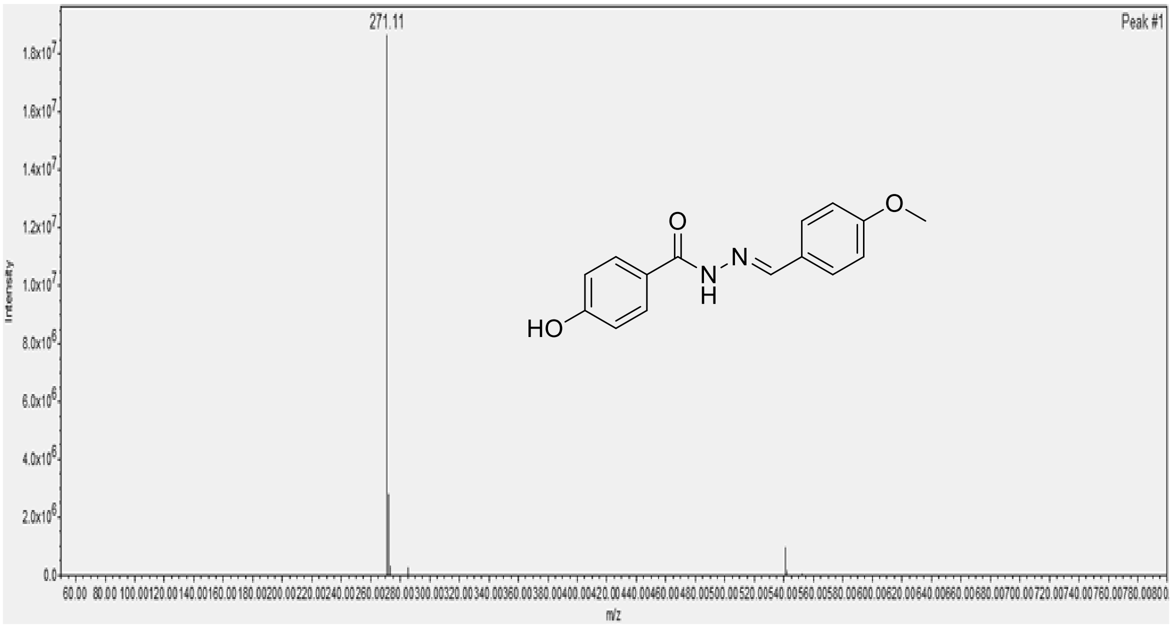

Spectrums of **Nfz-12**

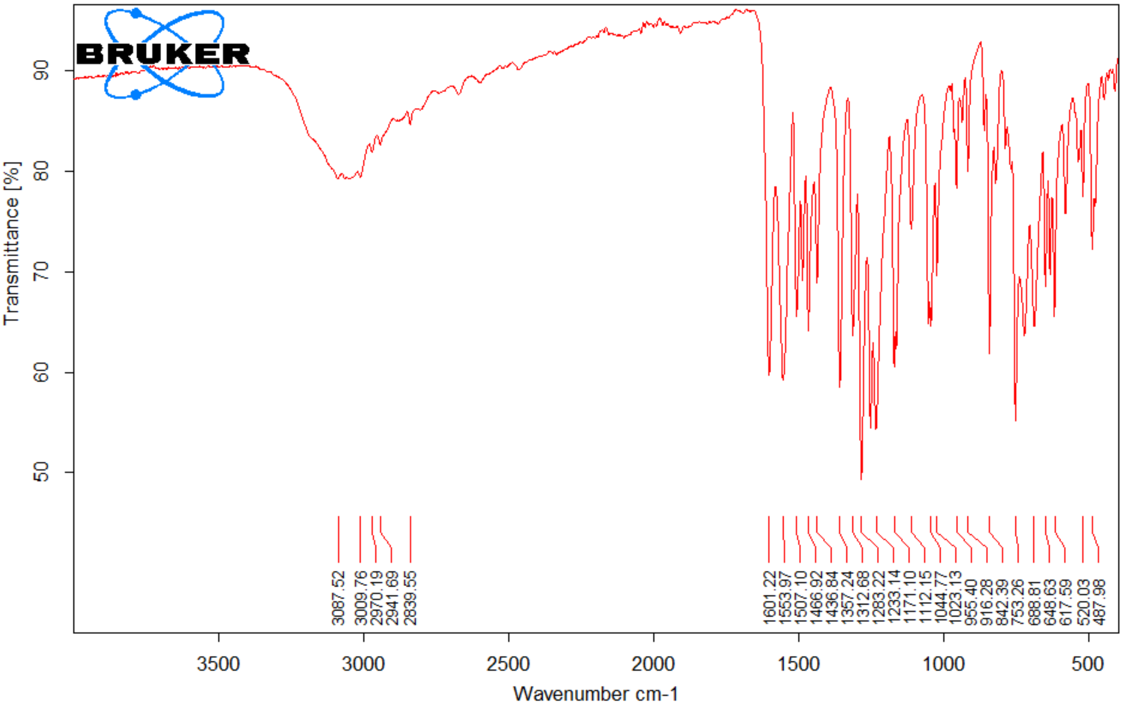
BRUKER
Transmittance [%]
Wavenumber cm-1
3087.52
3009.76
2970.19
2941.69
2839.55
1601.22
1553.97
1507.10
1466.92
1436.84
1357.24
1312.68
1283.22
1233.14
1171.10
1112.15
1044.77
1023.13
955.40
916.28
842.39
753.26
688.81
648.63
617.59
520.03
487.98

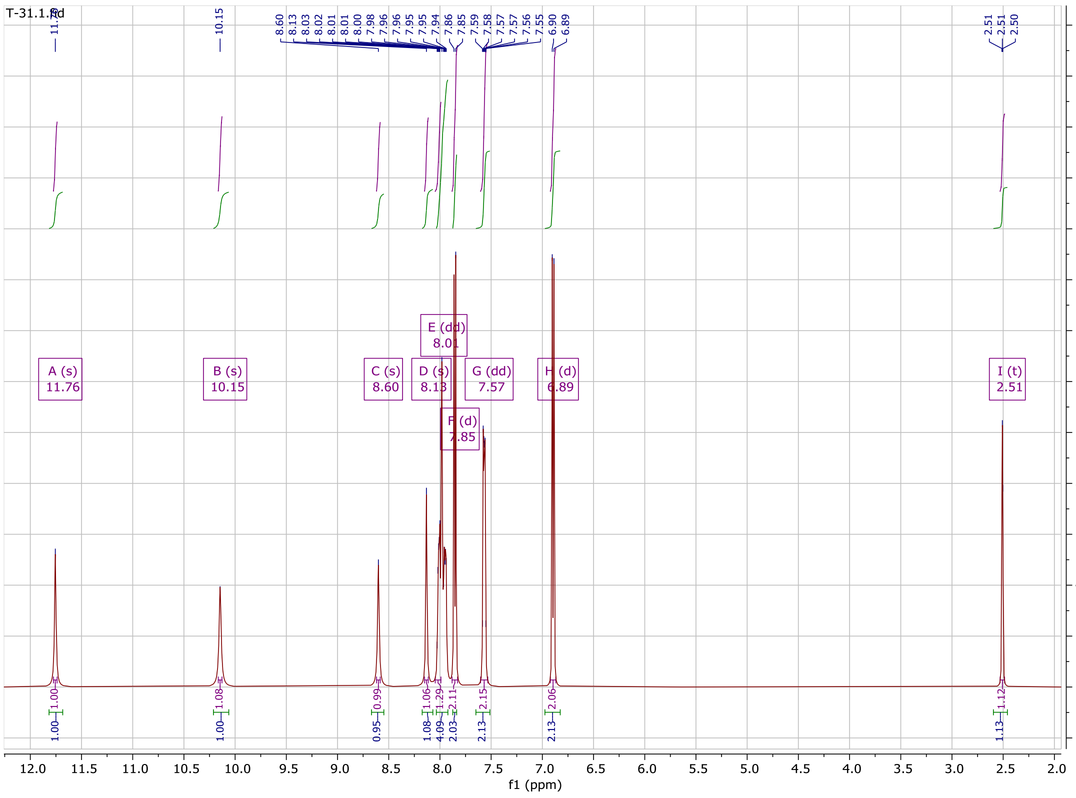
T-31.1.fid
A (s)
11.76
B (s)
10.15
C (s)
8.60
D (s)
8.13
E (dd)
8.01
F (d)
7.85
G (dd)
7.57
H (d)
6.89
I (t)
2.51
f1 (ppm)

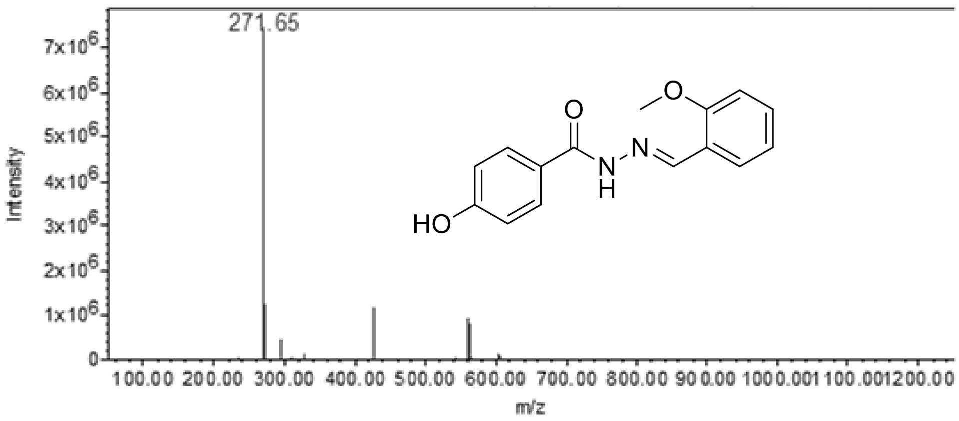

Spectrums of **Nfz-13**

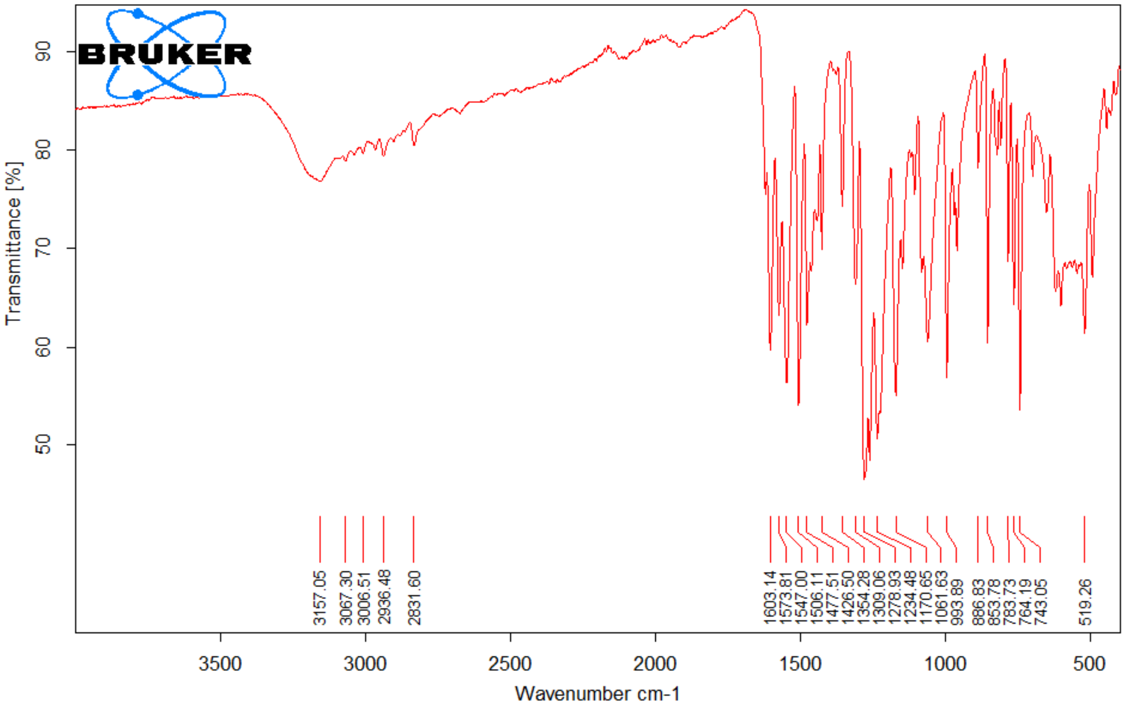

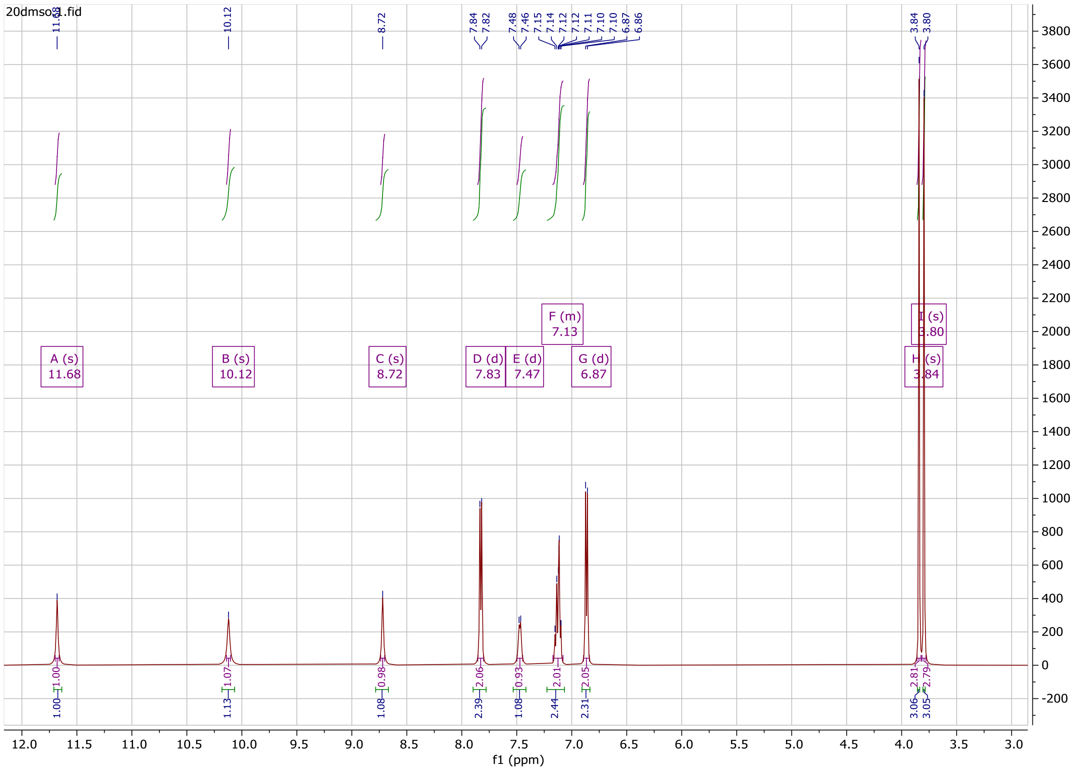
20dmso_1.fid
A (s) 11.68
B (s) 10.12
C (s) 8.72
D (d) 7.83
E (d) 7.47
F (m) 7.13
G (d) 6.87
H (s) 3.84
I (s) 3.80
f1 (ppm)

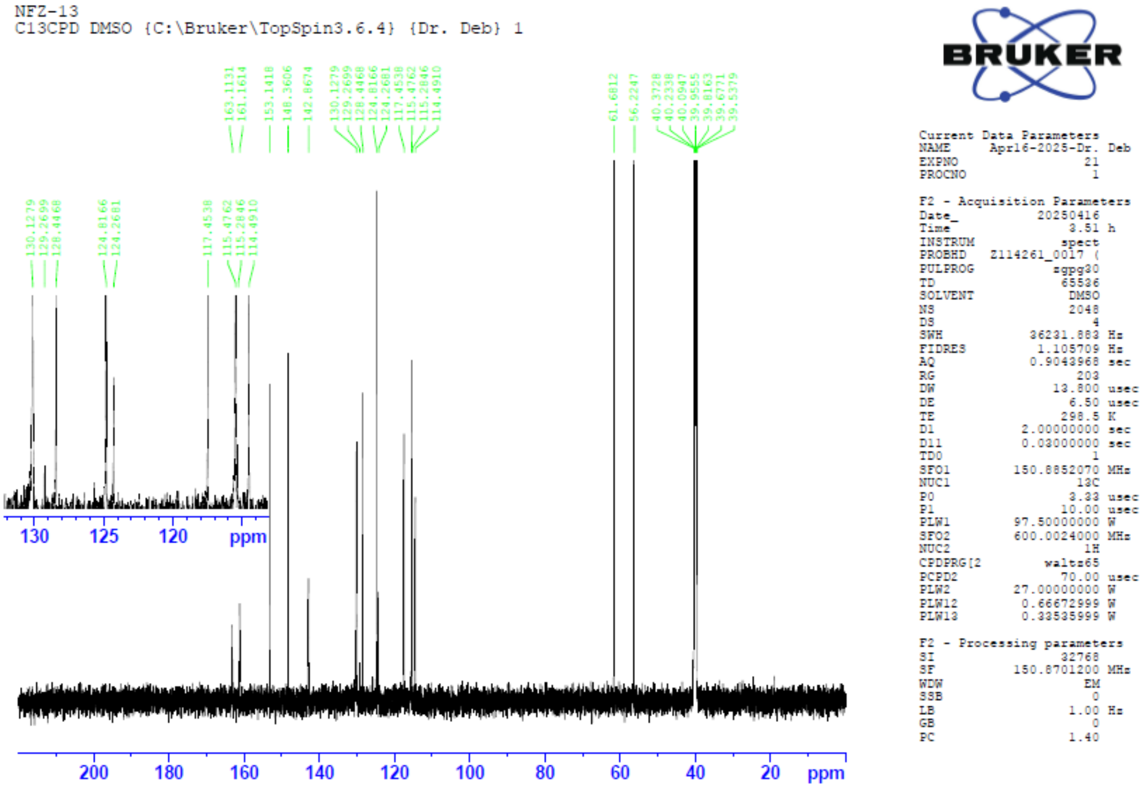
NFZ-13
C13CPD DMSO {C:\Bruker\TopSpin3.6.4} {Dr. Deb} 1
163.1131
161.1614
153.1418
148.3606
142.8674
130.1279
129.2699
128.4468
124.8166
124.2681
117.4538
115.4762
115.2846
114.4910
61.6812
56.2247
40.3728
40.2338
40.0947
39.9555
39.8163
39.6771
39.5379
ppm
BRUKER
Current Data Parameters
NAME Apr16-2025-Dr. Deb
EXPNO 21
PROCNO 1
F2 - Acquisition Parameters
Date_ 20250416
Time 3.51 h
INSTRUM spect
PROBHD Z114261_0017 (
PULPROG zgpg30
TD 65536
SOLVENT DMSO
NS 2048
DS 4
SWH 36231.883 Hz
FIDRES 1.105709 Hz
AQ 0.9043968 sec
RG 203
DW 13.800 usec
DE 6.50 usec
TE 298.5 K
D1 2.00000000 sec
D11 0.03000000 sec
TD0 1
SFO1 150.8852070 MHz
NUC1 13C
P0 3.33 usec
P1 10.00 usec
PLW1 97.50000000 W
SFO2 600.0024000 MHz
NUC2 1H
CPDPRG[2 waltz65
PCPD2 70.00 usec
PLW2 27.00000000 W
PLW12 0.66672999 W
PLW13 0.33535999 W
F2 - Processing parameters
SI 32768
SF 150.8701200 MHz
WDW EM
SSB 0
LB 1.00 Hz
GB 0
PC 1.40

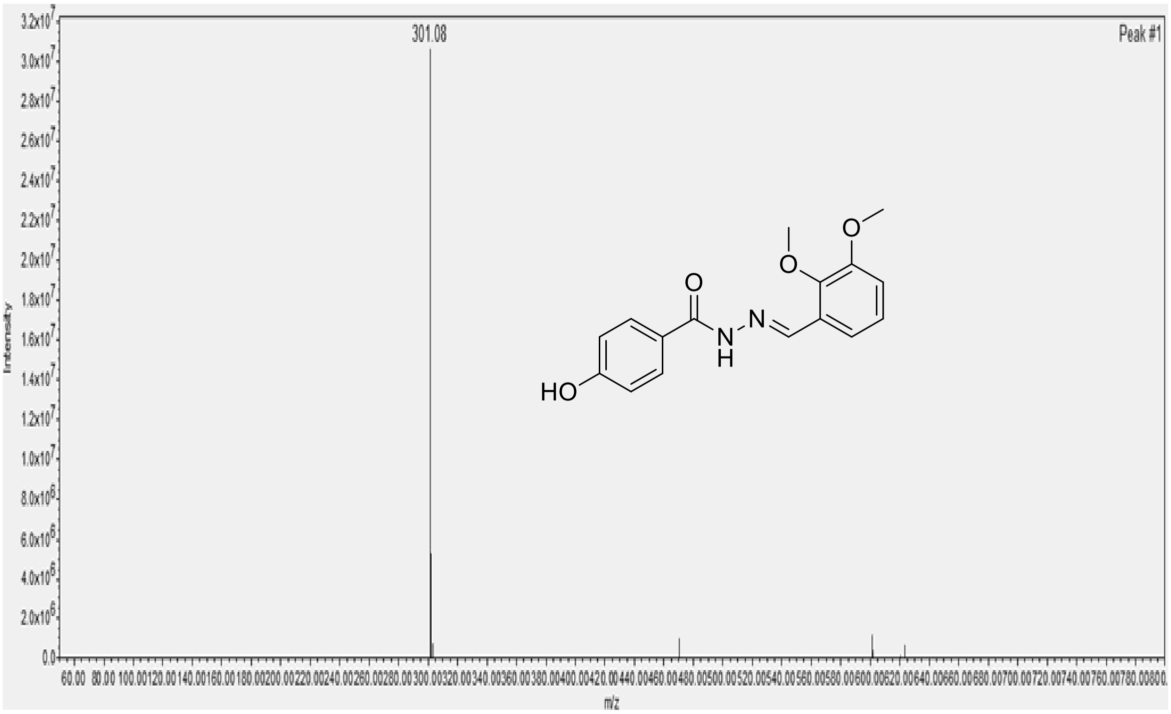

Spectrums of **Nfz-14**

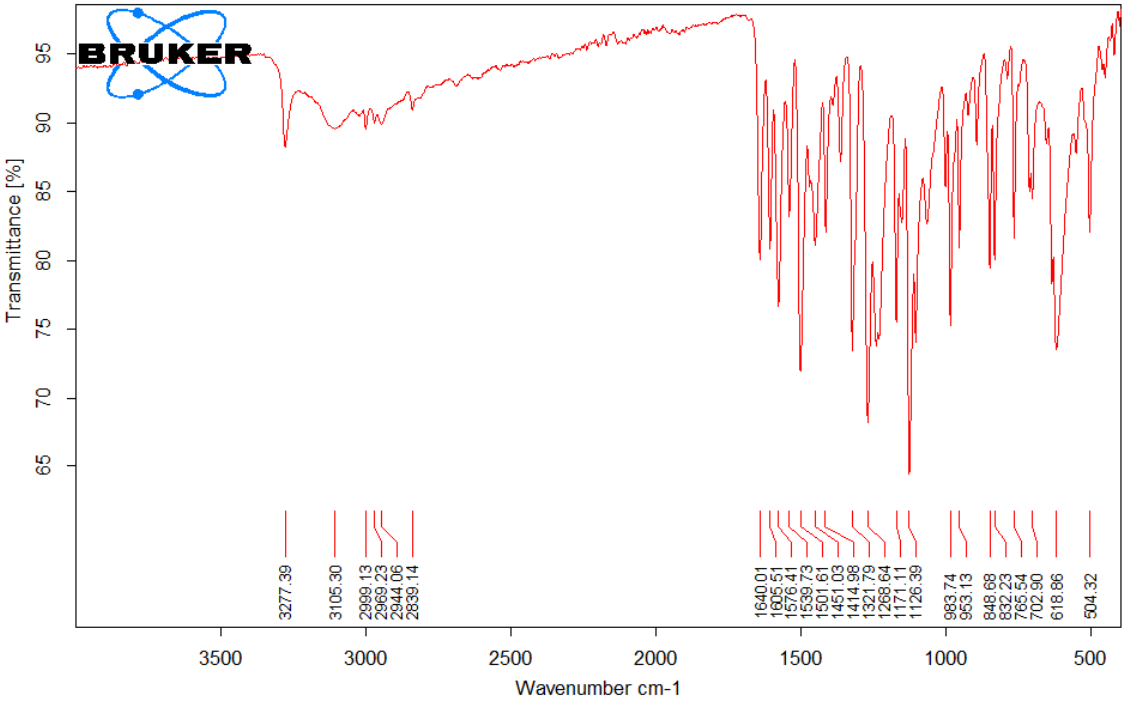

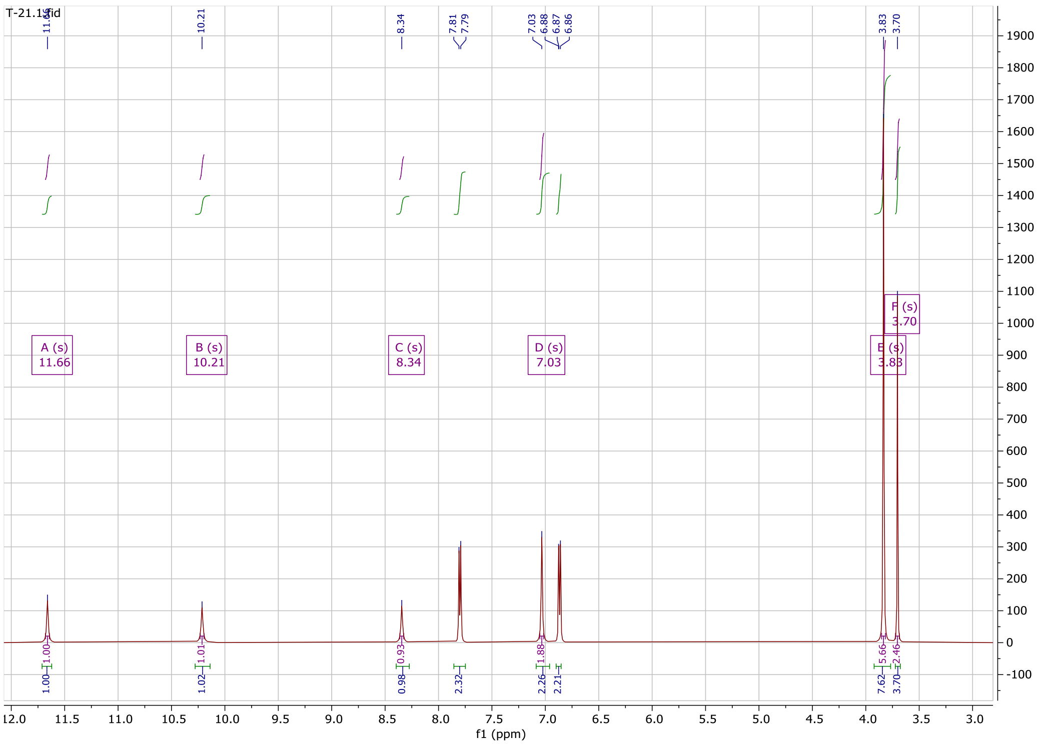
T-21.1.fid
A (s) 11.66
B (s) 10.21
C (s) 8.34
D (s) 7.03
E (s) 3.83
F (s) 3.70
f1 (ppm)

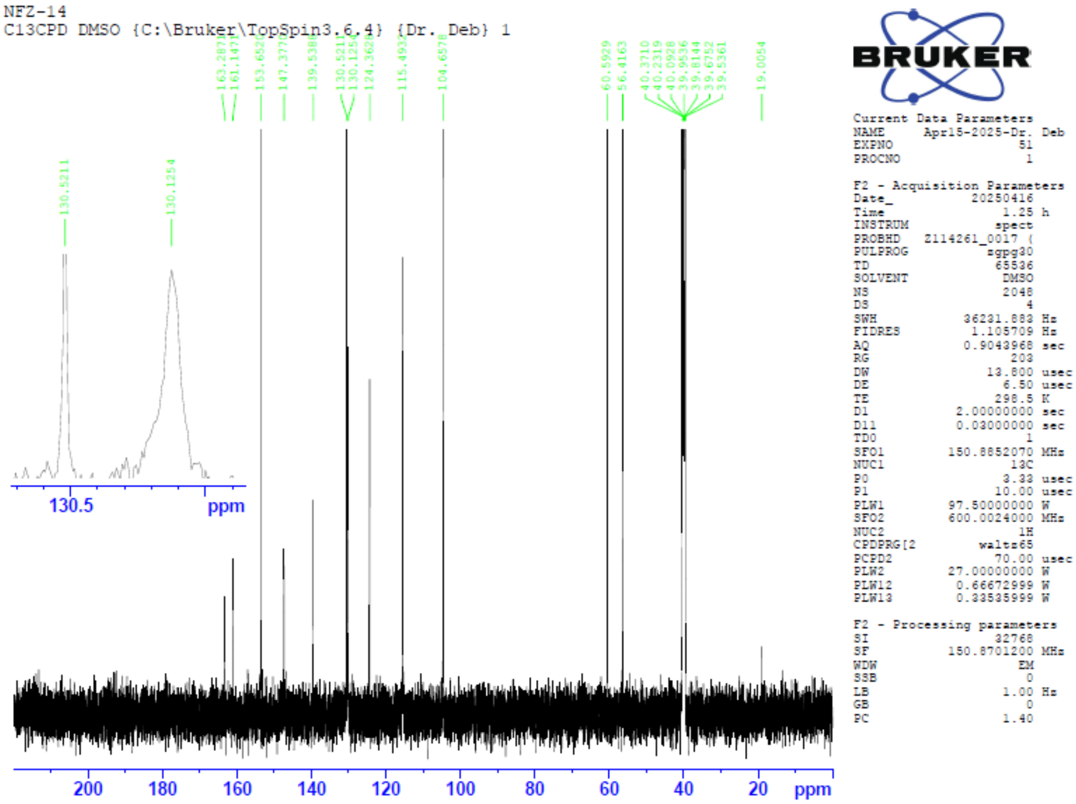
NFZ-14
C13CPD DMSO {C:\Bruker\TopSpin3.6.4} {Dr. Deb} 1
ppm
Current Data Parameters
NAME Apr15-2025-Dr. Deb
EXPNO 51
PROCNO 1
F2 - Acquisition Parameters
Date_ 20250416
Time 1.25 h
INSTRUM spect
PULPROG zgpg30
TD 65536
SOLVENT DMSO
NS 2048
DS 4
SWH 36231.883 Hz
FIDRES 1.105709 Hz
AQ 0.9043968 sec
RG 203
DW 13.800 usec
DE 6.50 usec
TE 298.5 K
D1 2.00000000 sec
D11 0.03000000 sec
TD0 1
SFO1 150.8852070 MHz
NUC1 13C
P0 3.33 usec
P1 10.00 usec
PLW1 97.50000000 W
SFO2 600.0024000 MHz
NUC2 1H
CPDPRG[2 waltz65
PCPD2 70.00 usec
PLW2 27.00000000 W
PLW12 0.66672999 W
PLW13 0.33535999 W
F2 - Processing parameters
SI 32768
SF 150.8701200 MHz
WDW EM
SSB 0
LB 1.00 Hz
GB 0
PC 1.40

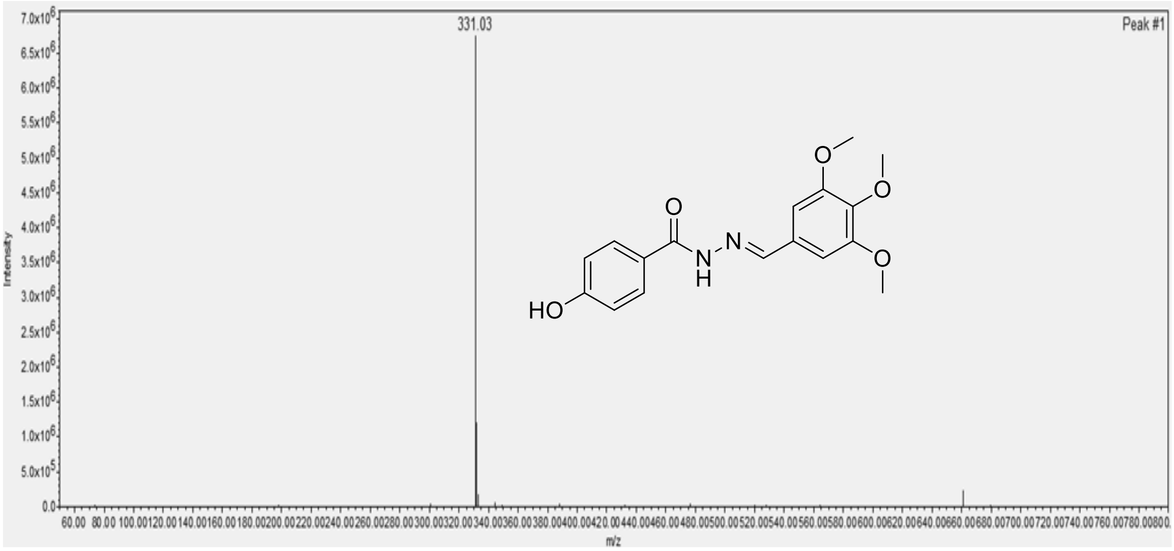

Spectrums of **Nfz-15**

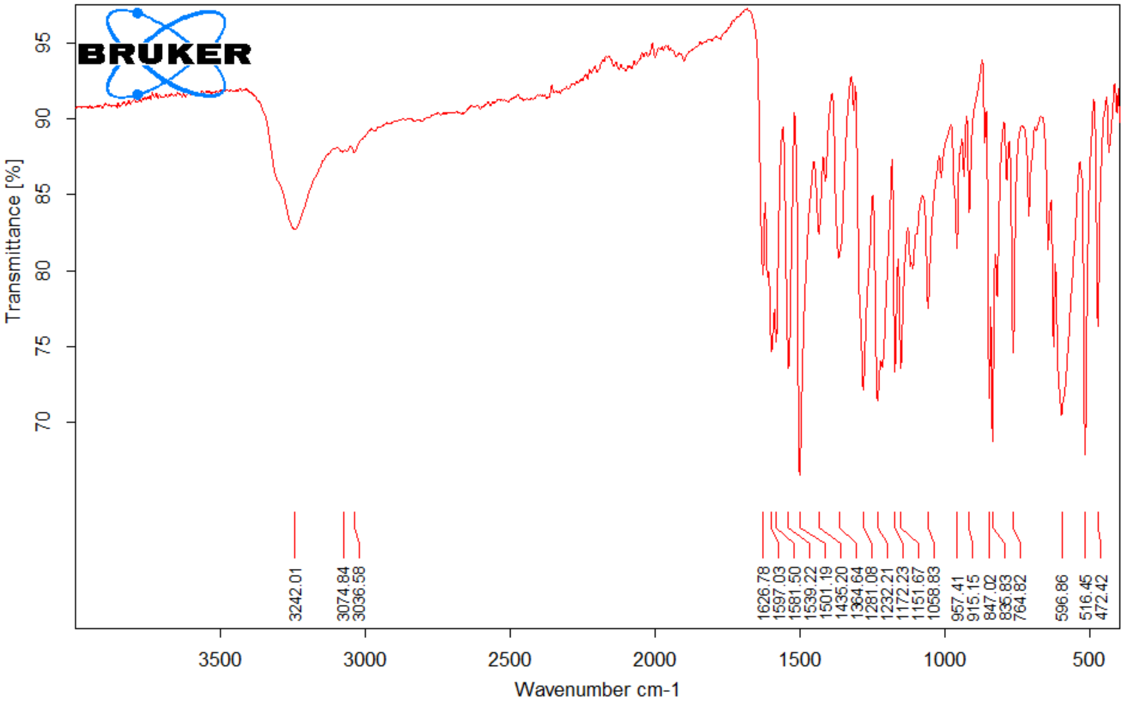

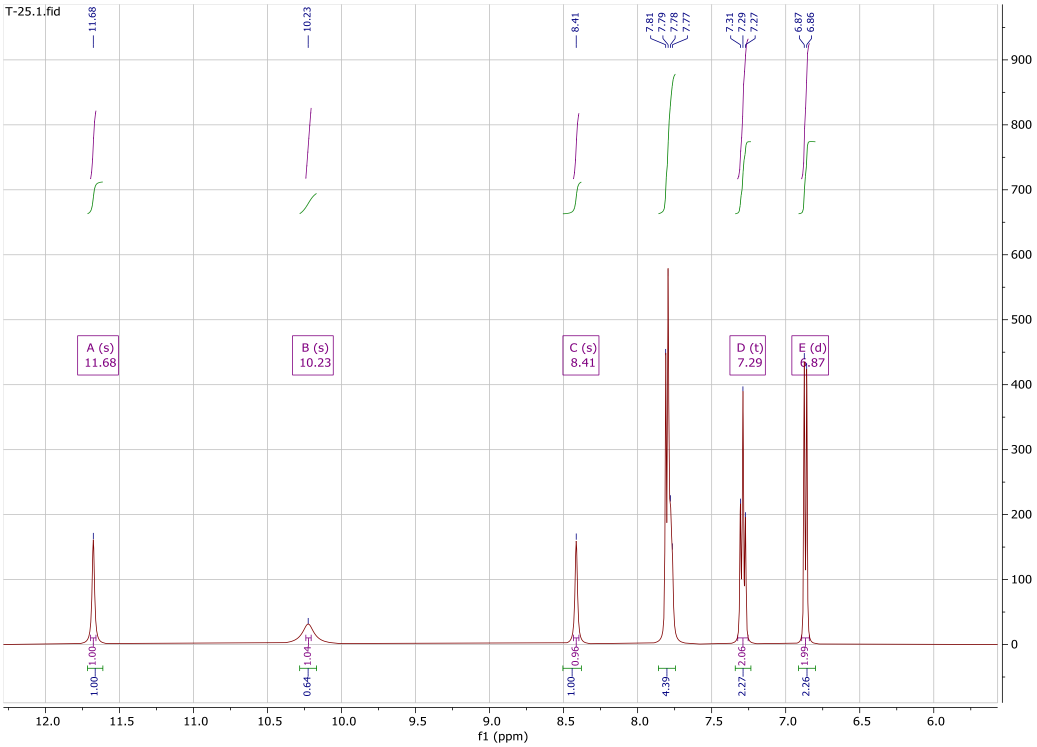

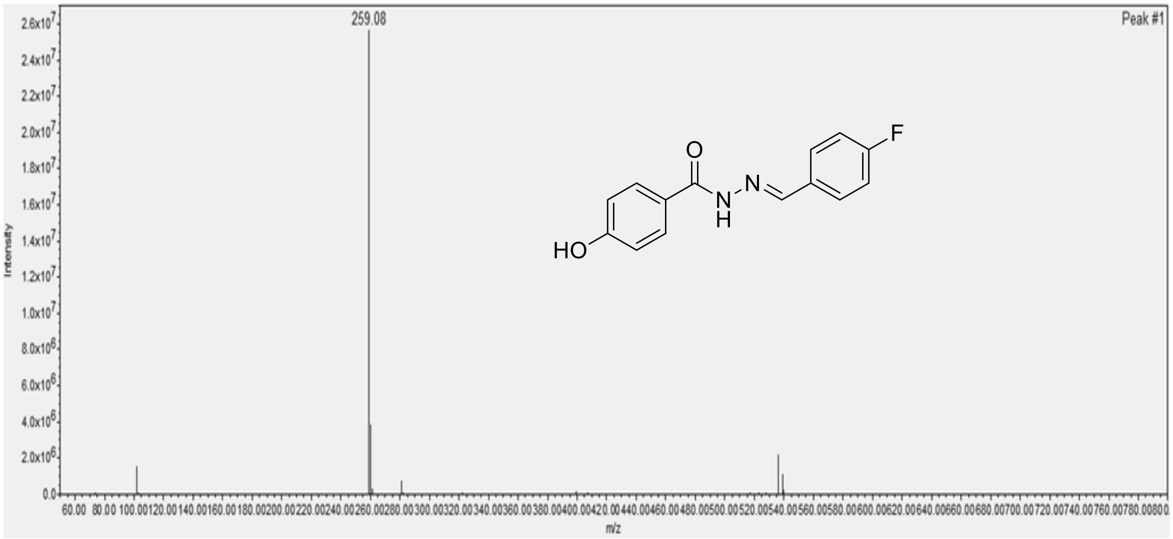

Spectrums of **Nfz-16**

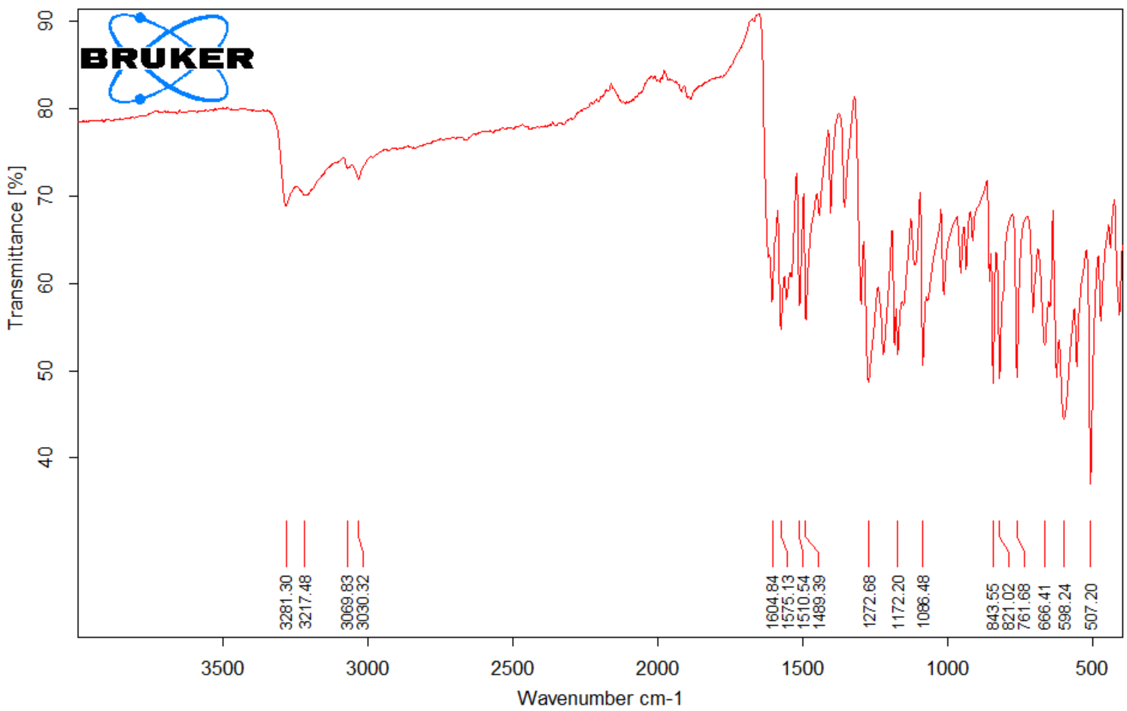

BRUKER
Transmittance [%]
Wavenumber cm-1
3281.30
3217.48
3069.83
3030.32
1604.84
1575.13
1510.54
1489.39
1272.68
1172.20
1086.48
843.55
821.02
761.68
666.41
598.24
507.20

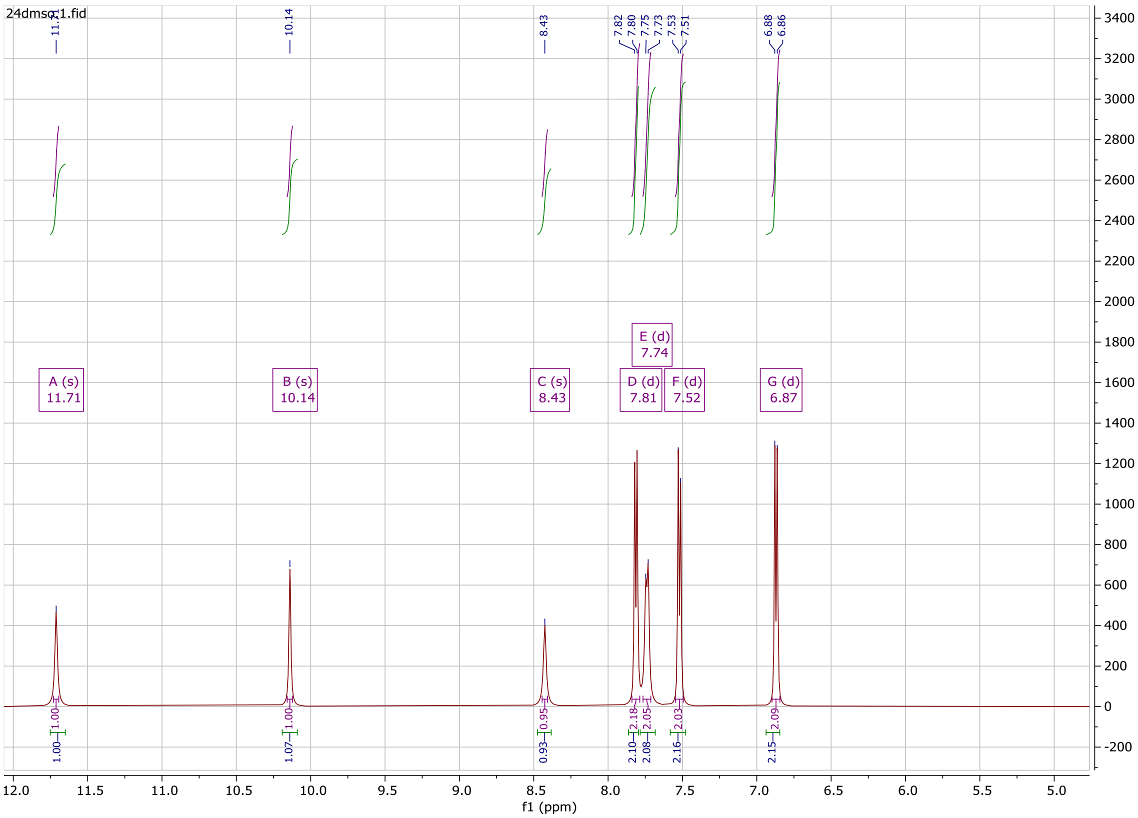

24dmso.1.fid
A (s)
11.71
B (s)
10.14
C (s)
8.43
D (d)
7.81
E (d)
7.74
F (d)
7.52
G (d)
6.87
f1 (ppm)

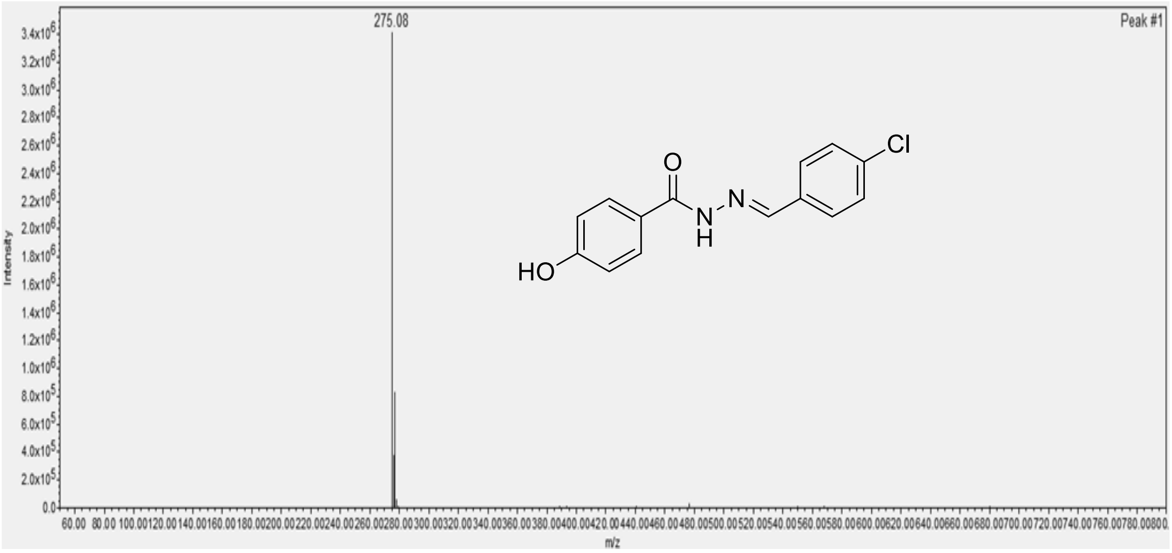

Spectrums of **Nfz-17**

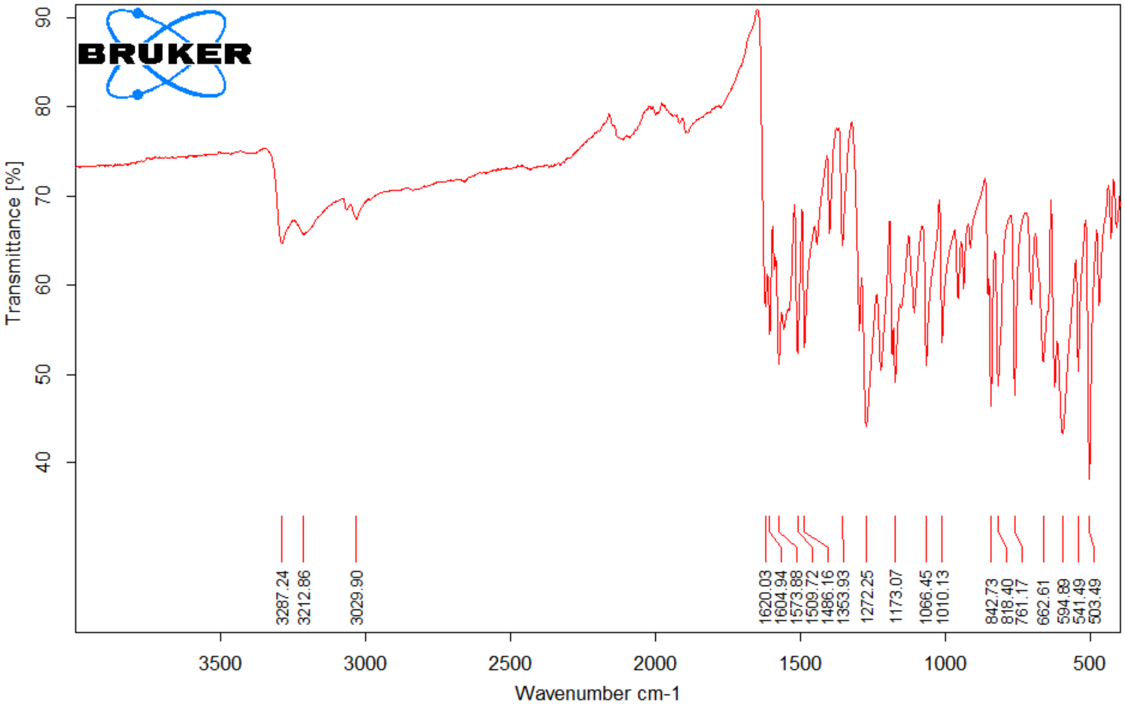

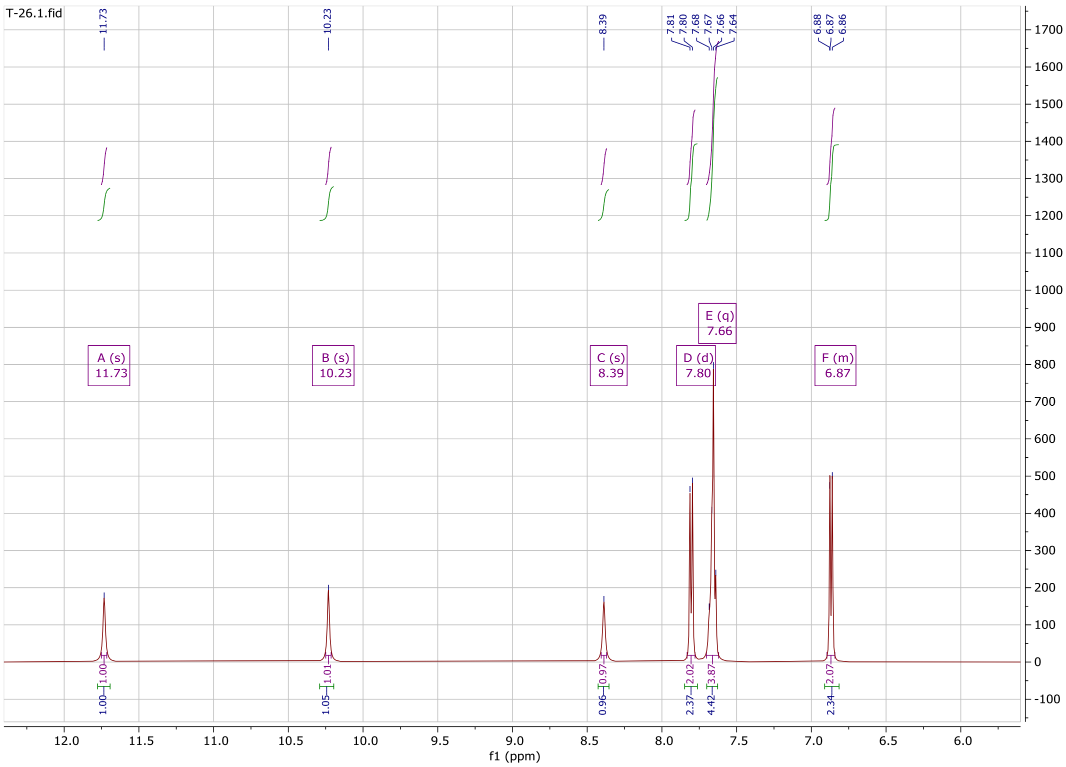

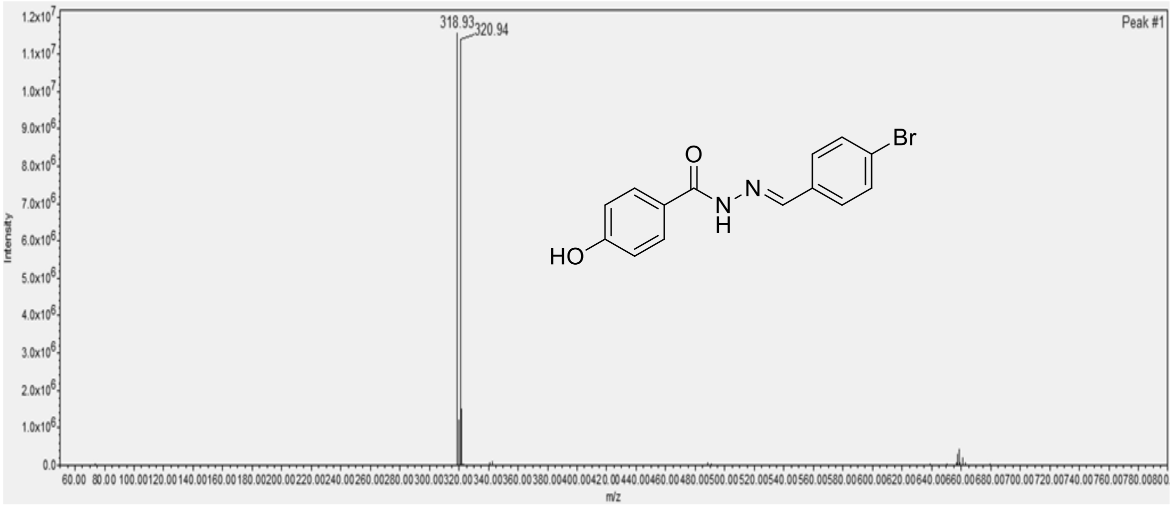

Spectrums of **Nfz-18**

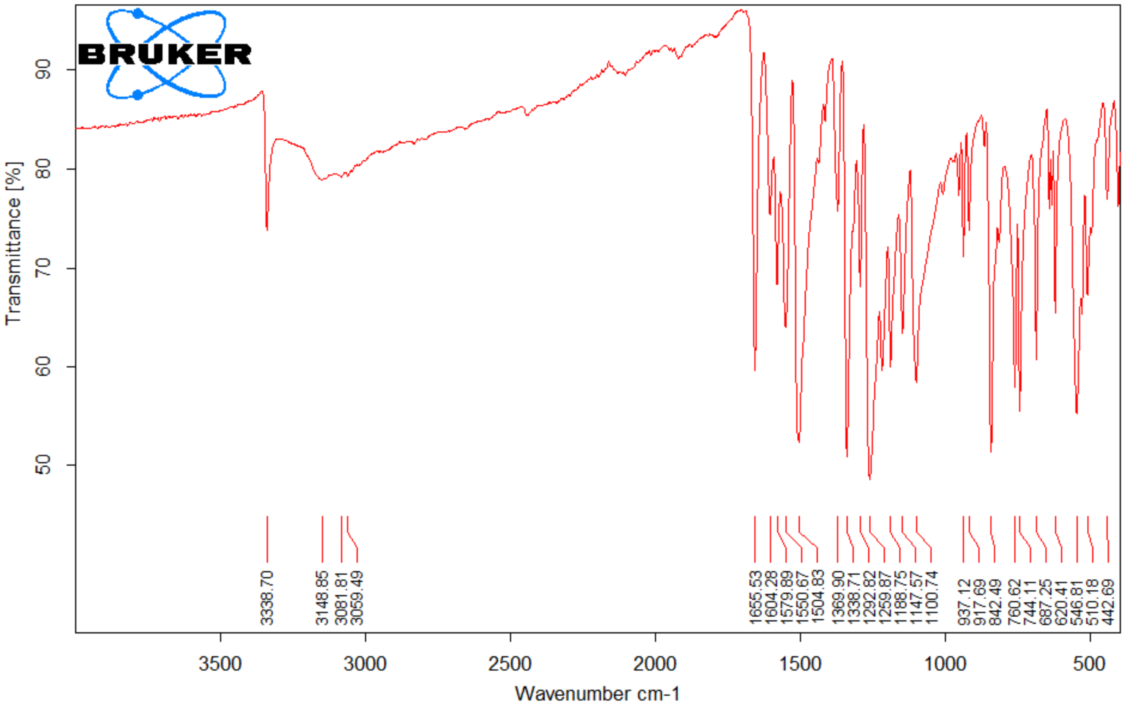
BRUKER
Transmittance [%]
Wavenumber cm-1
3338.70
3148.85
3081.81
3059.49
1655.53
1604.28
1579.89
1550.67
1504.83
1369.90
1338.71
1292.82
1259.87
1188.75
1147.57
1100.74
937.12
917.69
842.49
760.62
744.11
687.25
620.41
546.81
510.18
442.69

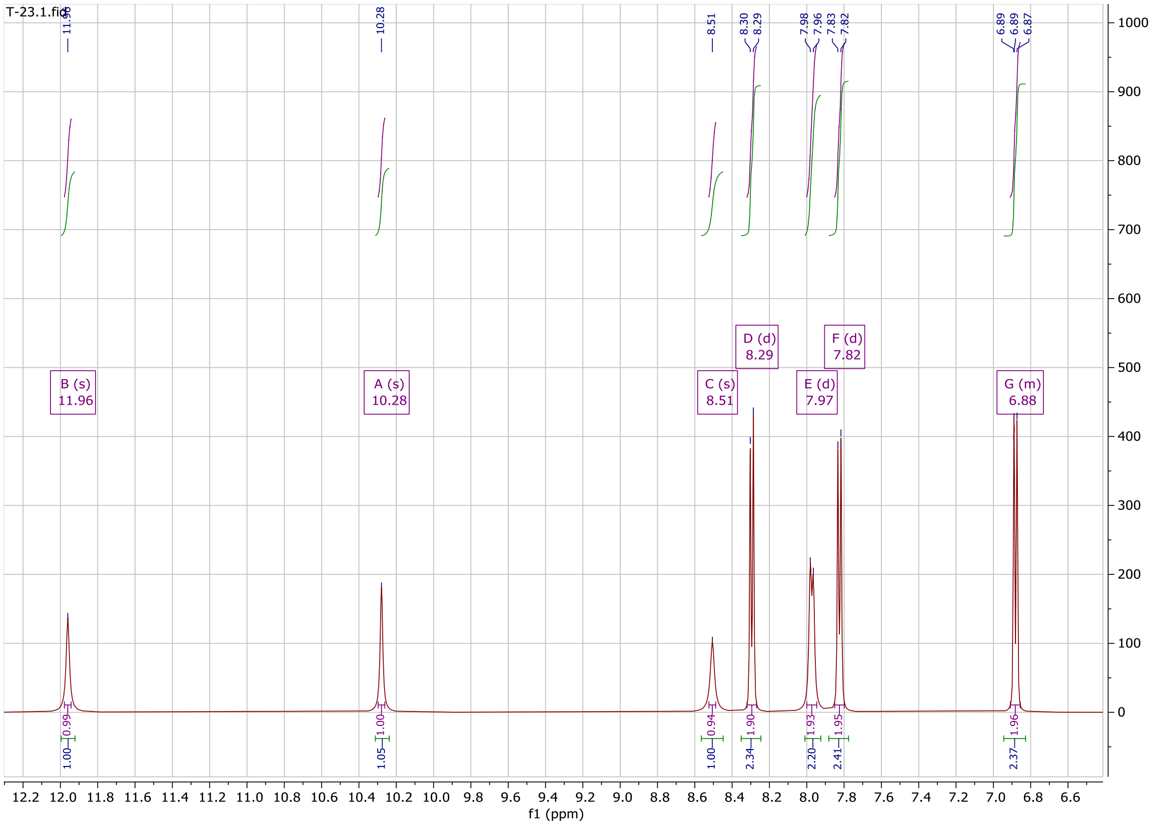
T-23.1.fid
B (s)
11.96
A (s)
10.28
C (s)
8.51
D (d)
8.29
E (d)
7.97
F (d)
7.82
G (m)
6.88
f1 (ppm)

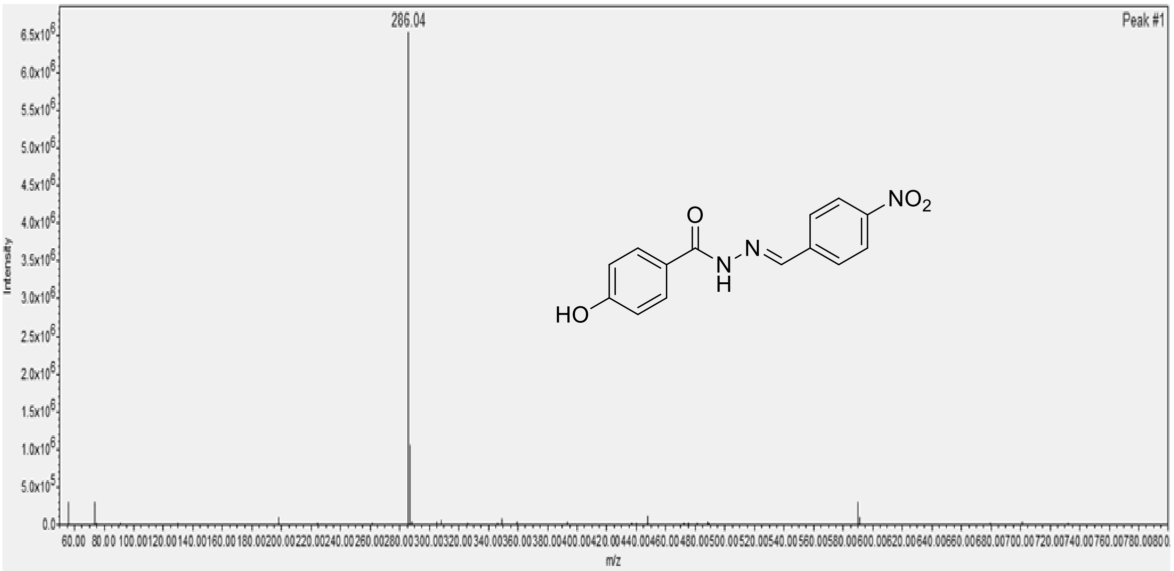

Spectrums of **Nfz-19**

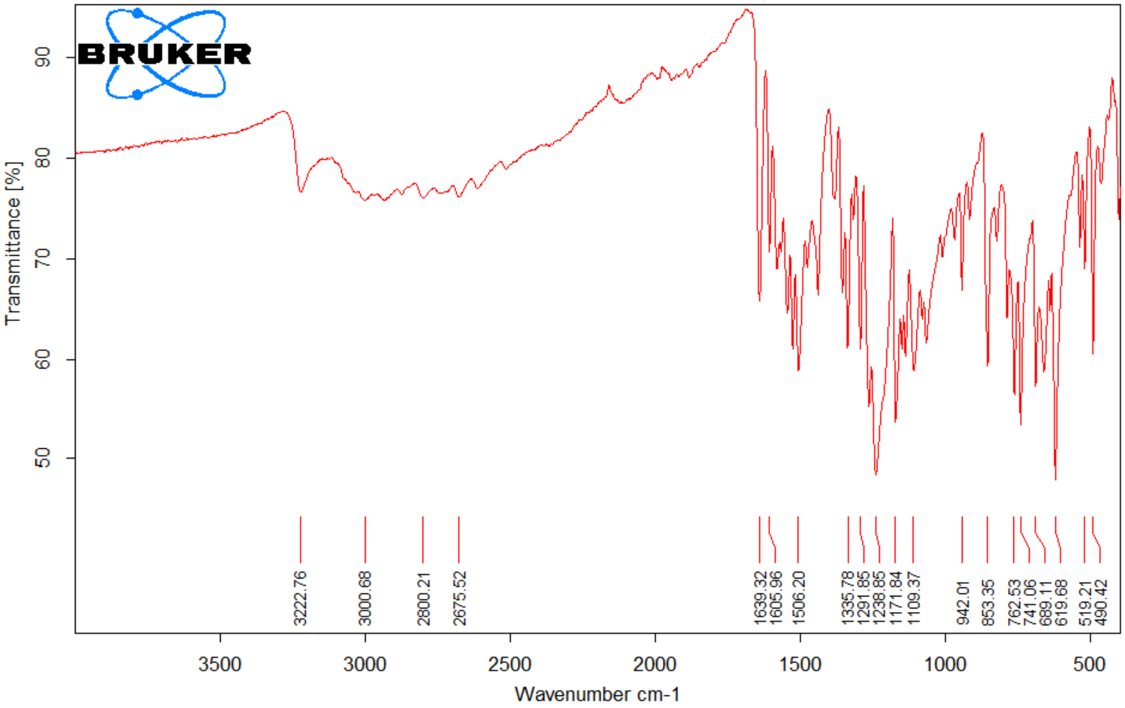

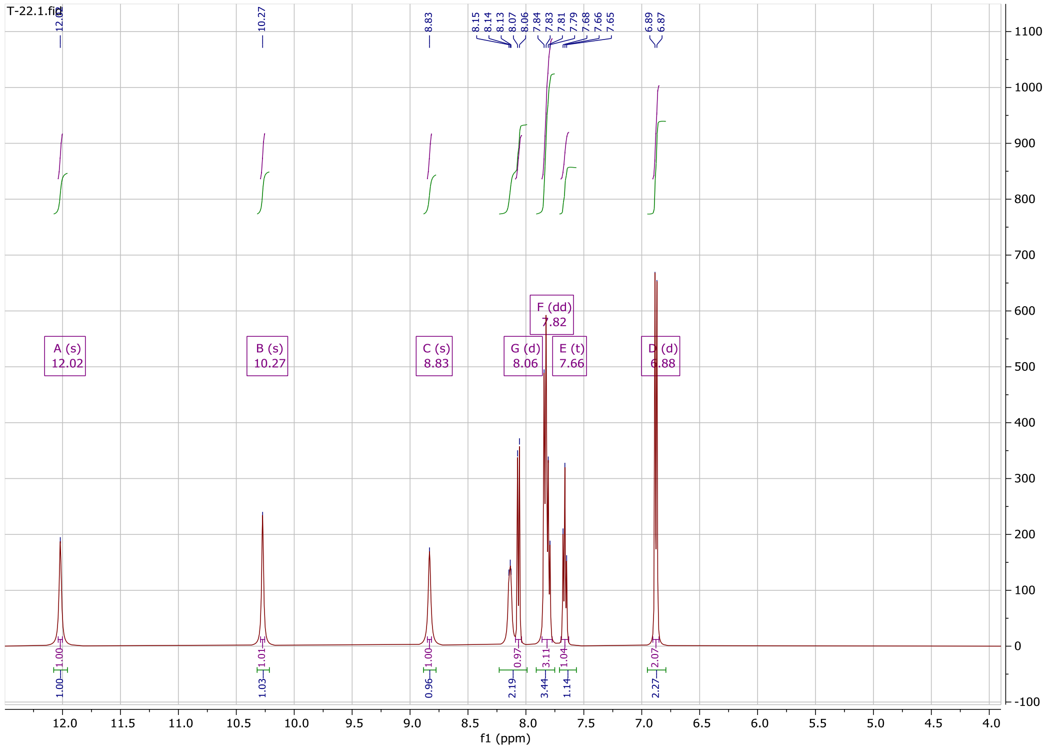

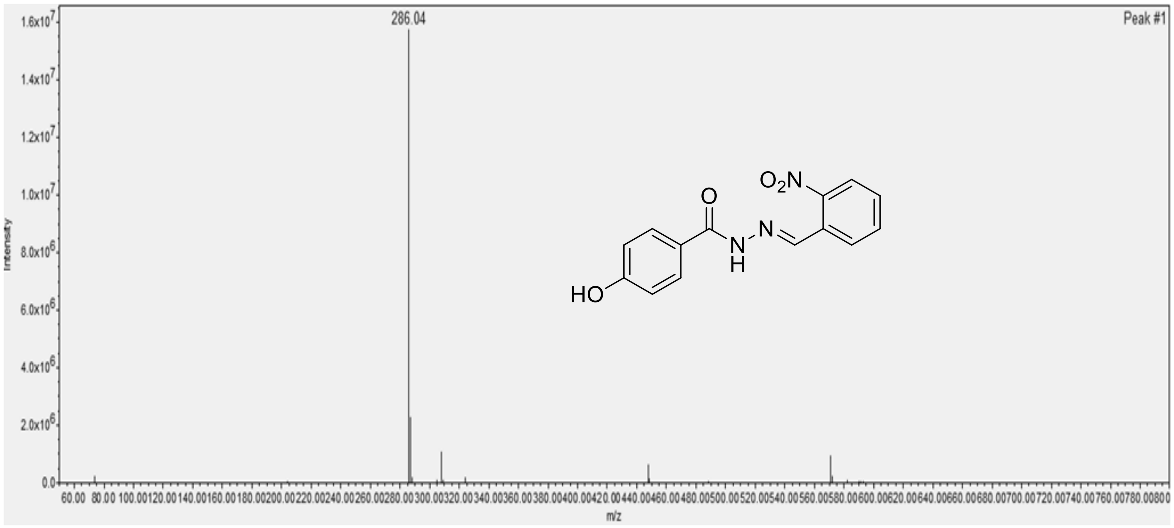

Spectrums of **Nfz-20**

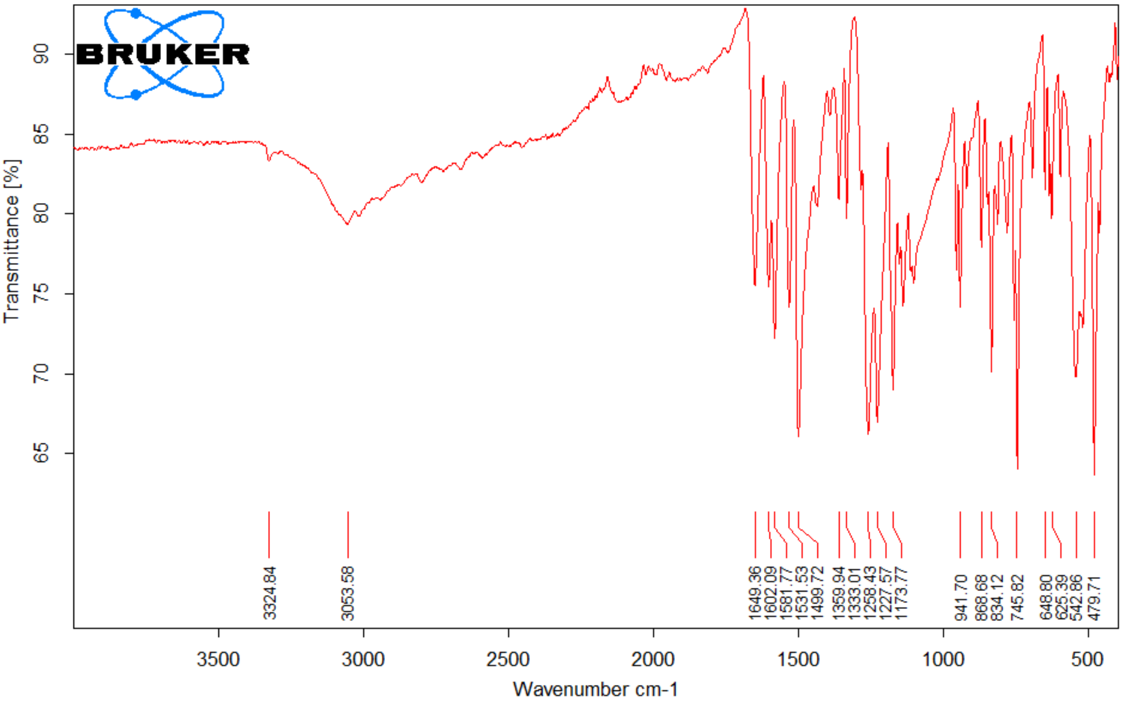
BRUKER
Transmittance [%]
Wavenumber cm-1
3324.84
3053.58
1649.36
1602.09
1581.77
1531.53
1499.72
1359.94
1333.01
1258.43
1227.57
1173.77
941.70
868.68
834.12
745.82
648.80
625.39
542.86
479.71

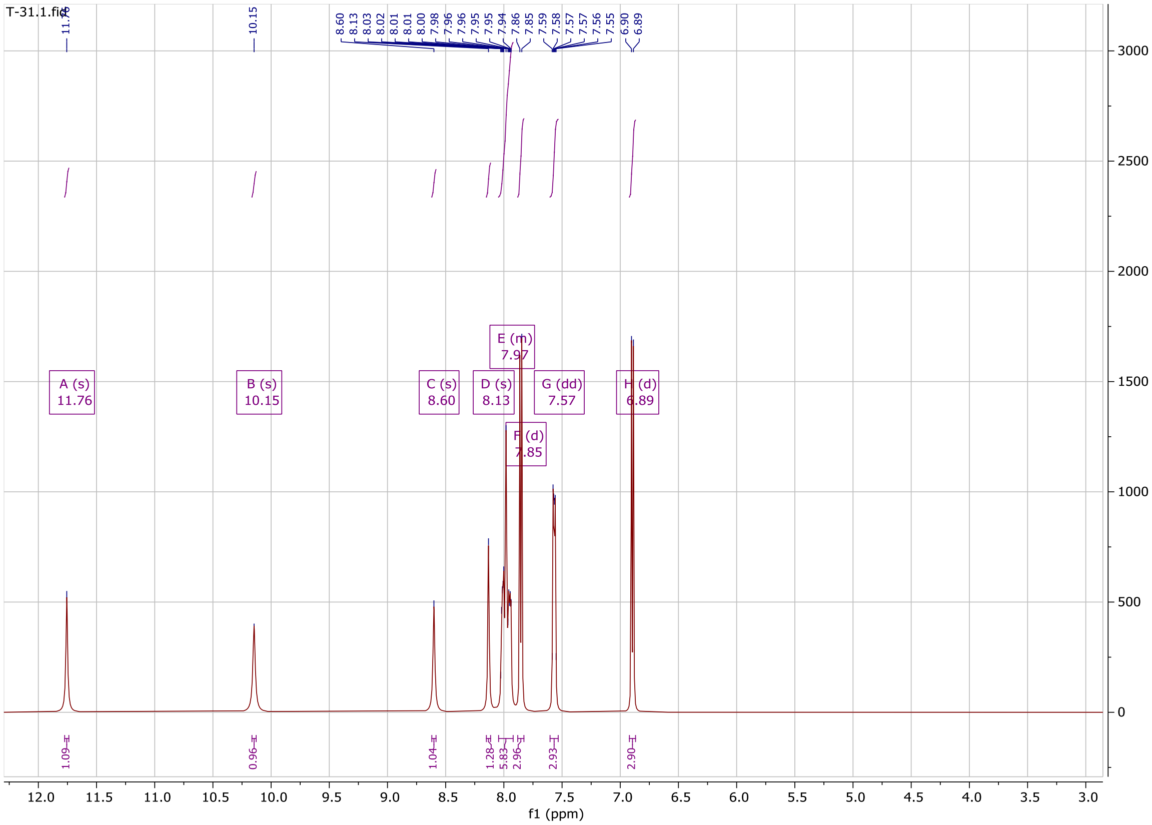
T-31.1.fid
A (s) 11.76
B (s) 10.15
C (s) 8.60
D (s) 8.13
E (m) 7.97
F (d) 7.85
G (dd) 7.57
H (d) 6.89
1.09
0.96
1.04
1.28
5.83
2.96
2.93
2.90
f1 (ppm)

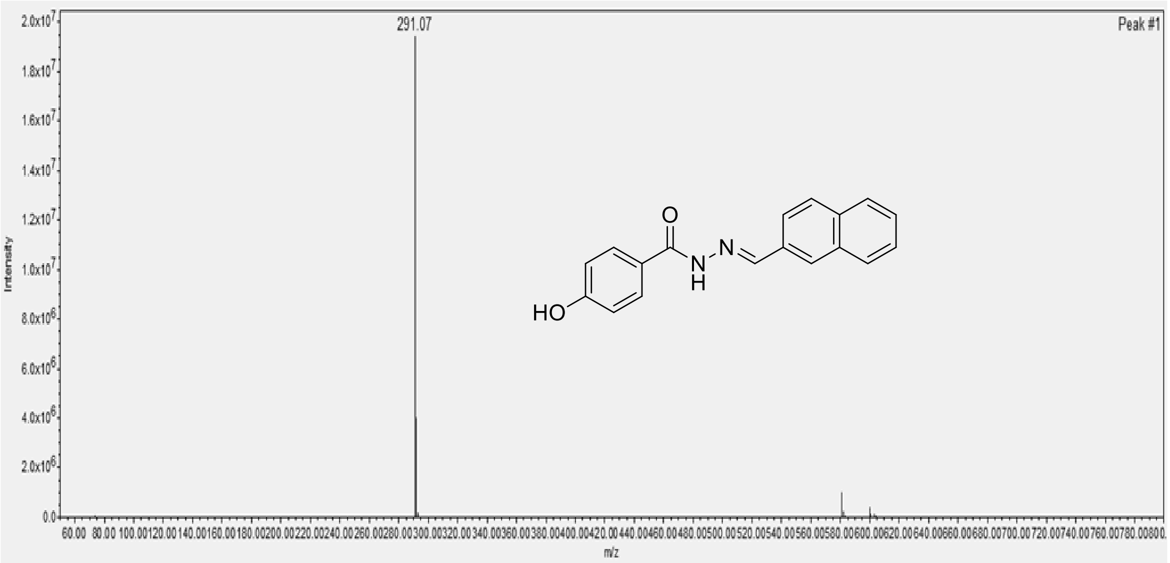

Spectrums of **Nfz-21**

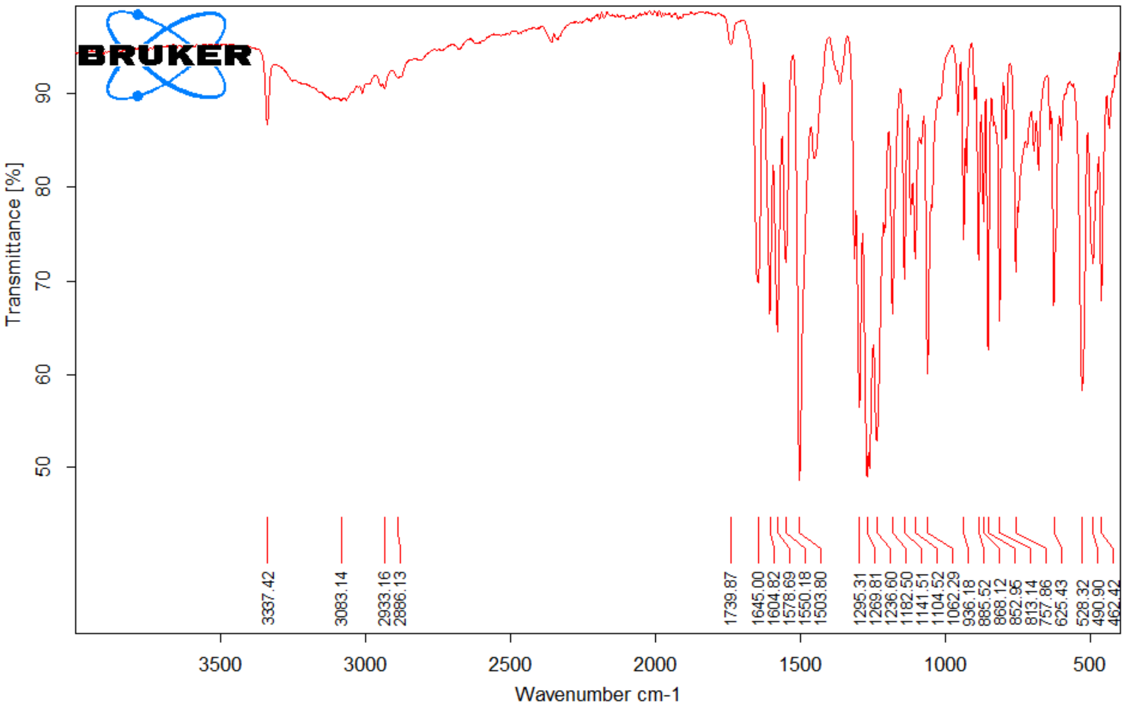

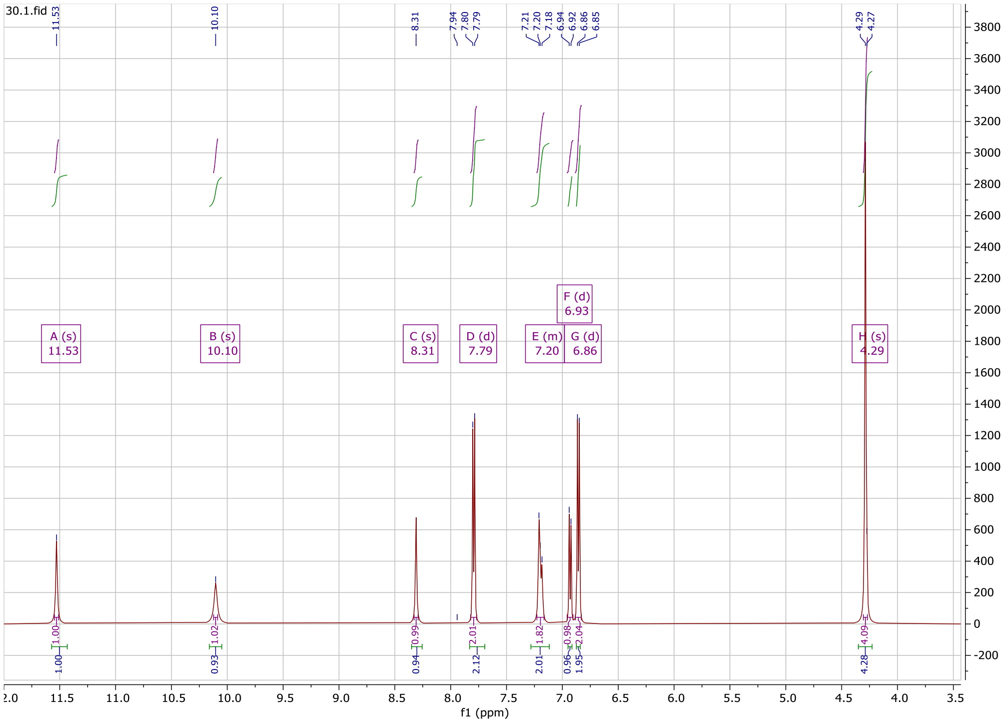

30.1.fid
11.53
10.10
8.31
7.94
7.80
7.79
7.21
7.20
7.18
6.94
6.92
6.86
6.85
4.29
4.27
A (s)
11.53
B (s)
10.10
C (s)
8.31
D (d)
7.79
E (m)
7.20
F (d)
6.93
G (d)
6.86
H (s)
4.29
1.00
1.02
0.99
2.01
1.82
0.98
2.04
4.09
1.00
0.93
0.94
2.12
2.01
0.96
1.95
4.28
f1 (ppm)

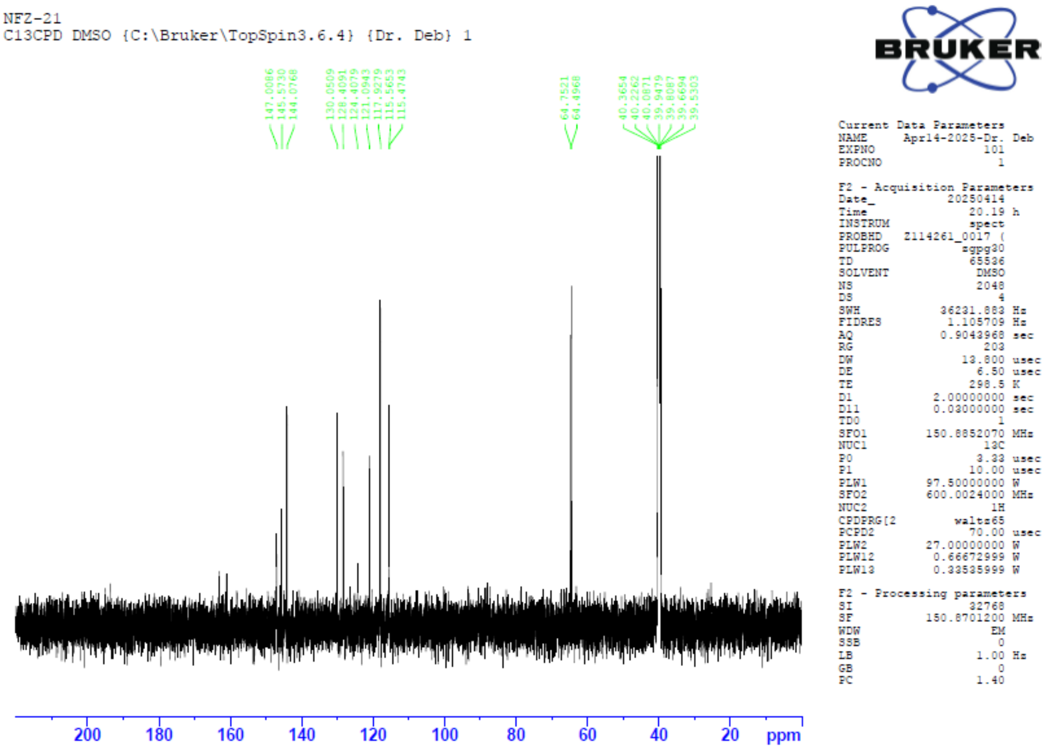

NFZ-21
C13CPD DMSO {C:\Bruker\TopSpin3.6.4} {Dr. Deb} 1
147.0086
145.5730
144.0768
130.0509
128.4051
124.4079
121.0943
117.9279
115.5653
115.4743
64.7521
64.4968
40.3654
40.2262
40.0871
39.9479
39.8087
39.6694
39.5303
ppm
Current Data Parameters
NAME Apr14-2025-Dr. Deb
EXPNO 101
PROCNO 1
F2 - Acquisition Parameters
Date_ 20250414
Time 20.19 h
INSTRUM spect
PROBHD Z114261_0017 (
PULPROG zgpg30
TD 65536
SOLVENT DMSO
NS 2048
DS 4
SWH 36231.883 Hz
FIDRES 1.105709 Hz
AQ 0.9043968 sec
RG 203
DW 13.800 usec
DE 6.50 usec
TE 298.5 K
D1 2.00000000 sec
D11 0.03000000 sec
TD0 1
SFO1 150.8852070 MHz
NUC1 13C
P0 3.33 usec
P1 10.00 usec
PLW1 97.50000000 W
SFO2 600.0024000 MHz
NUC2 1H
CPDPRG[2 waltz65
PCPD2 70.00 usec
PLW2 27.00000000 W
PLW12 0.66672999 W
PLW13 0.33535999 W
F2 - Processing parameters
SI 32768
SF 150.8701200 MHz
WDW EM
SSB 0
LB 1.00 Hz
GB 0
PC 1.40

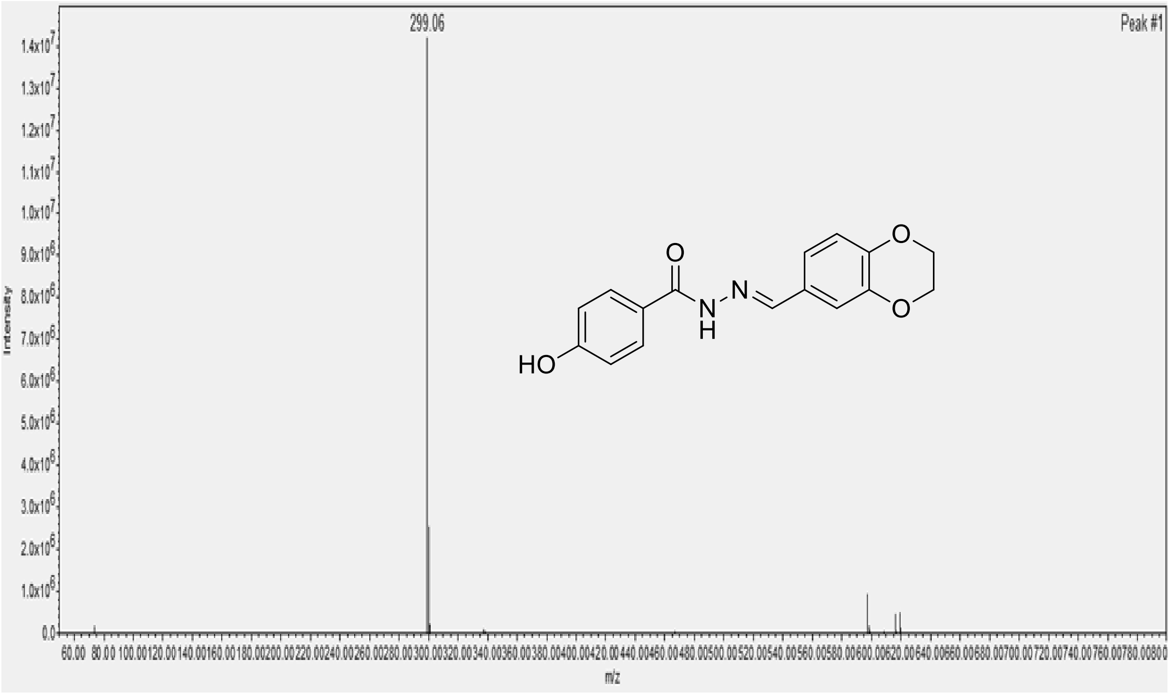

Spectrums of **Nfz-22**

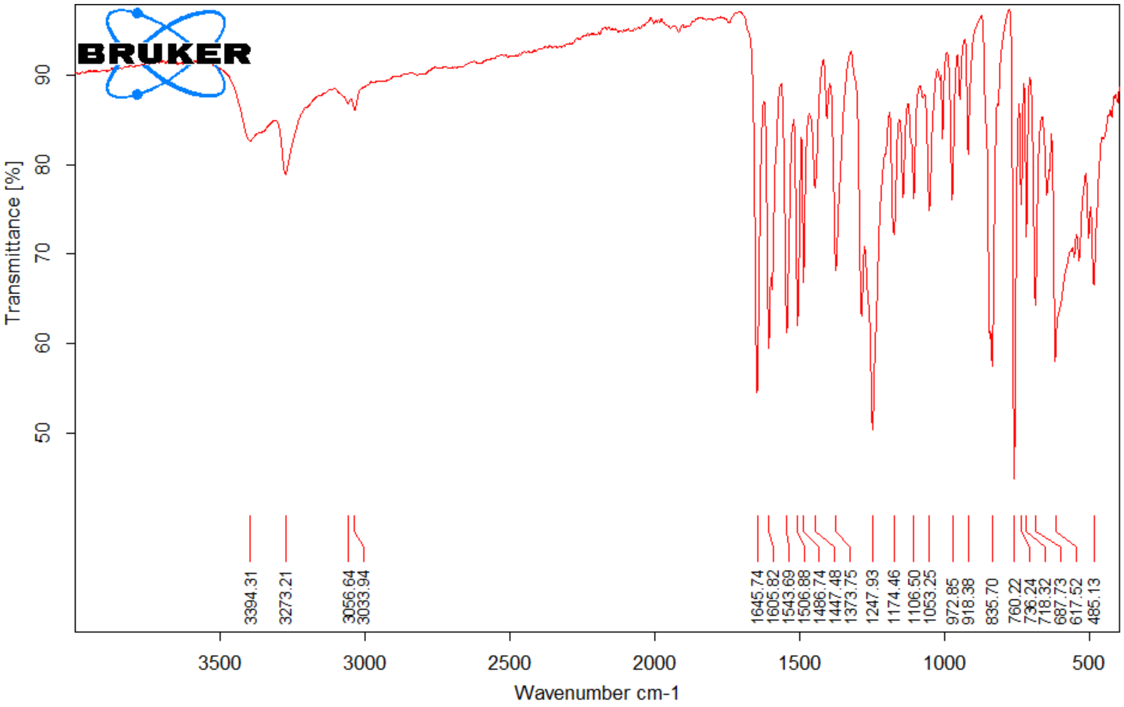

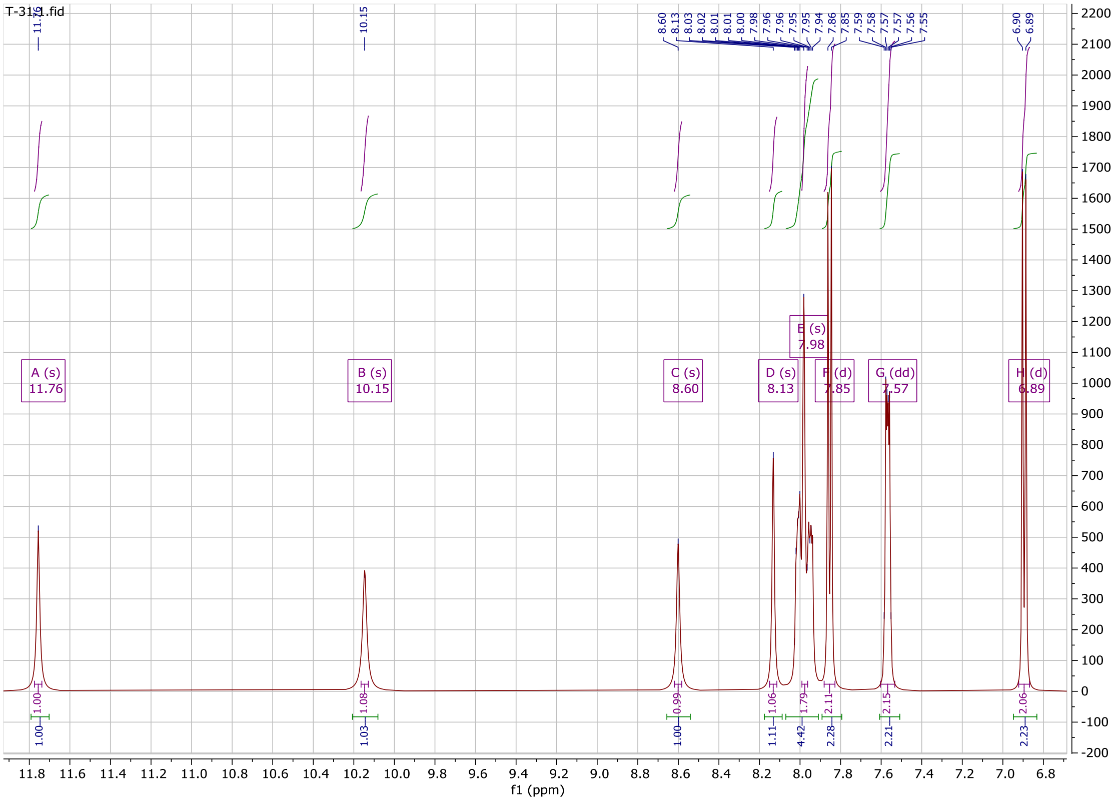

T-31.1.fid
11.76
10.15
8.60 8.13 8.03 8.02 8.01 8.01 8.00 7.98 7.96 7.96 7.95 7.95 7.94 7.86 7.85 7.59 7.58 7.57 7.57 7.56 7.55
6.90 6.89
A (s) 11.76
B (s) 10.15
C (s) 8.60
D (s) 8.13
E (s) 7.98
F (d) 7.85
G (dd) 7.57
H (d) 6.89
1.00 1.08 0.99 1.06 1.79 2.11 2.15 2.06
1.00 1.03 1.00 1.11 4.42 2.28 2.21 2.23
f1 (ppm)

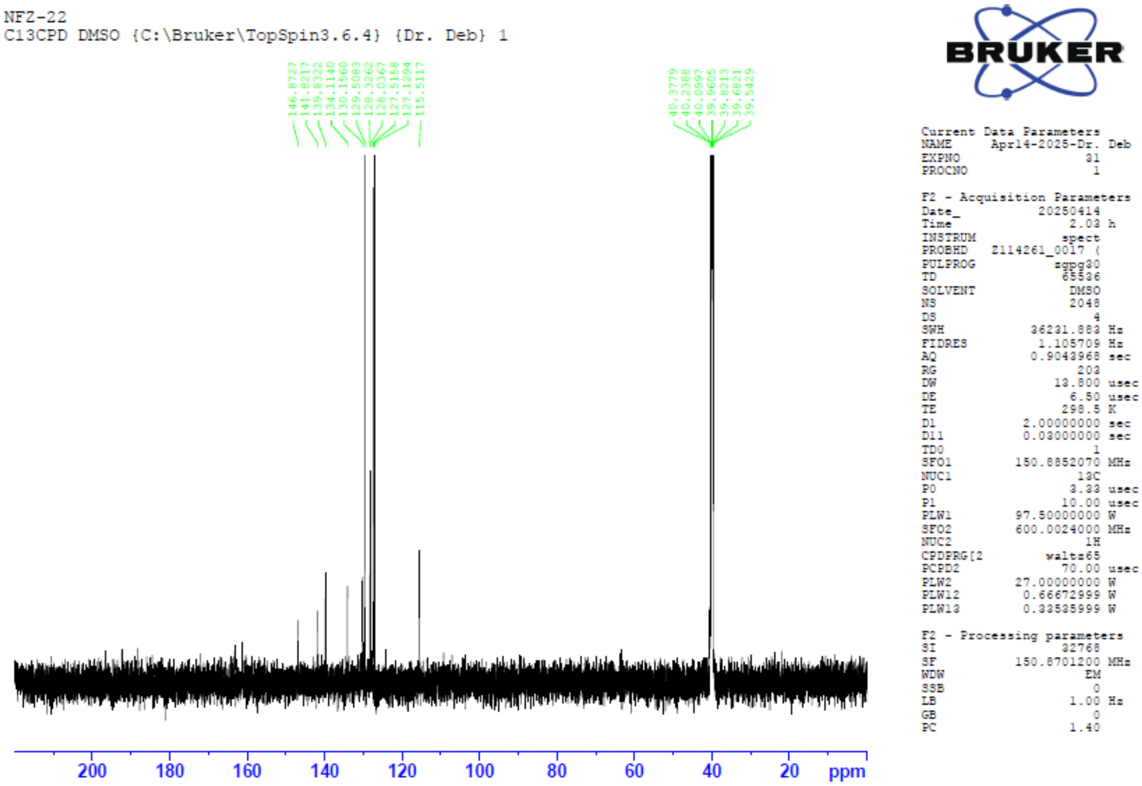

NFZ-22
C13CPD DMSO {C:\Bruker\TopSpin3.6.4} {Dr. Deb} 1
146.8727 141.8217 139.8322 134.1140 130.1560 129.5083 128.3262 128.0367 127.5158 127.1294 115.5117
40.3779 40.2388 40.0997 39.9605 39.8213 39.6821 39.5429
BRUKER
Current Data Parameters
NAME Apr14-2025-Dr. Deb
EXPNO 31
PROCNO 1
F2 - Acquisition Parameters
Date_ 20250414
Time 2.03 h
INSTRUM spect
PROBHD Z114261_0017 (
PULPROG zgpg30
TD 65536
SOLVENT DMSO
NS 2048
DS 4
SWH 36231.883 Hz
FIDRES 1.105709 Hz
AQ 0.9043968 sec
RG 203
DW 13.800 usec
DE 6.50 usec
TE 298.5 K
D1 2.00000000 sec
D11 0.03000000 sec
TD0 1
SFO1 150.8852070 MHz
NUC1 13C
P0 3.33 usec
P1 10.00 usec
PLW1 97.50000000 W
SFO2 600.0024000 MHz
NUC2 1H
CPDPRG[2 waltz65
PCPD2 70.00 usec
PLW2 27.00000000 W
PLW12 0.66672999 W
PLW13 0.33535999 W
F2 - Processing parameters
SI 32768
SF 150.8701200 MHz
WDW EM
SSB 0
LB 1.00 Hz
GB 0
PC 1.40
ppm

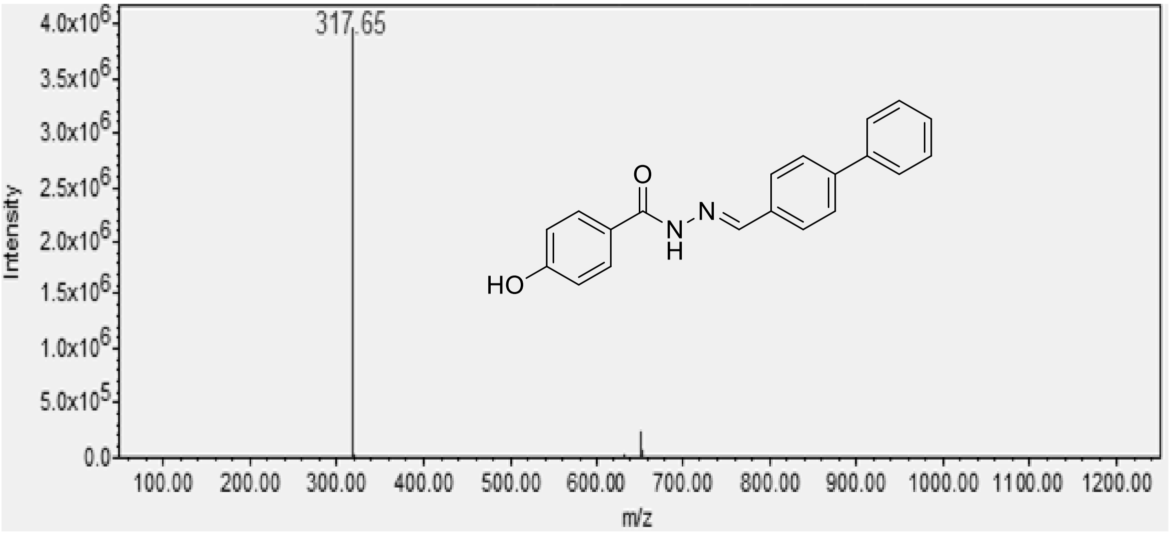

Spectrums of **Nfz-23**

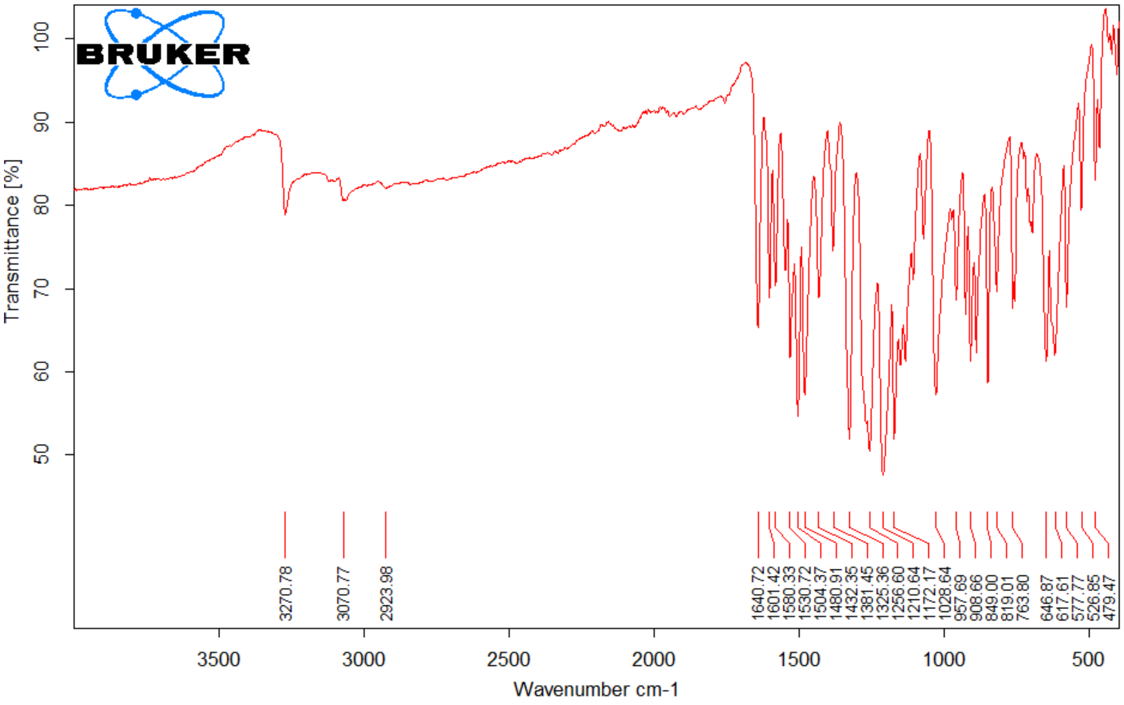

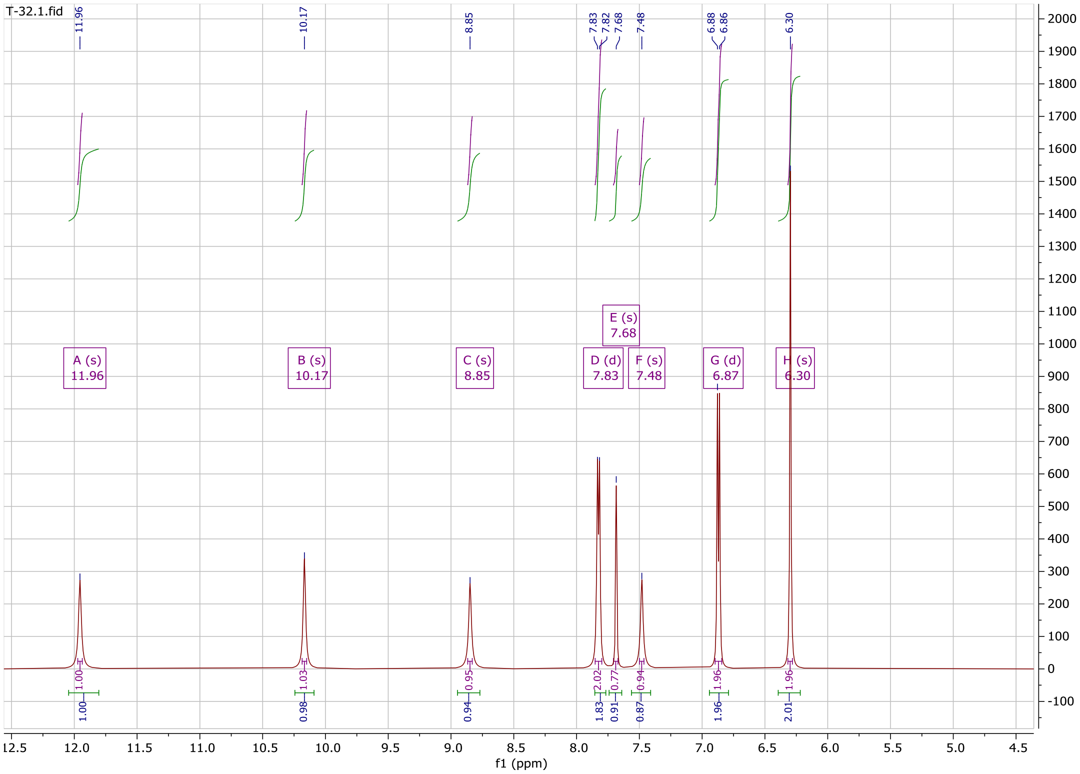

NFZ-23
C13CPD DMSO {C:\Bruker\TopSpin3.6.4} {Dr. Deb} 1

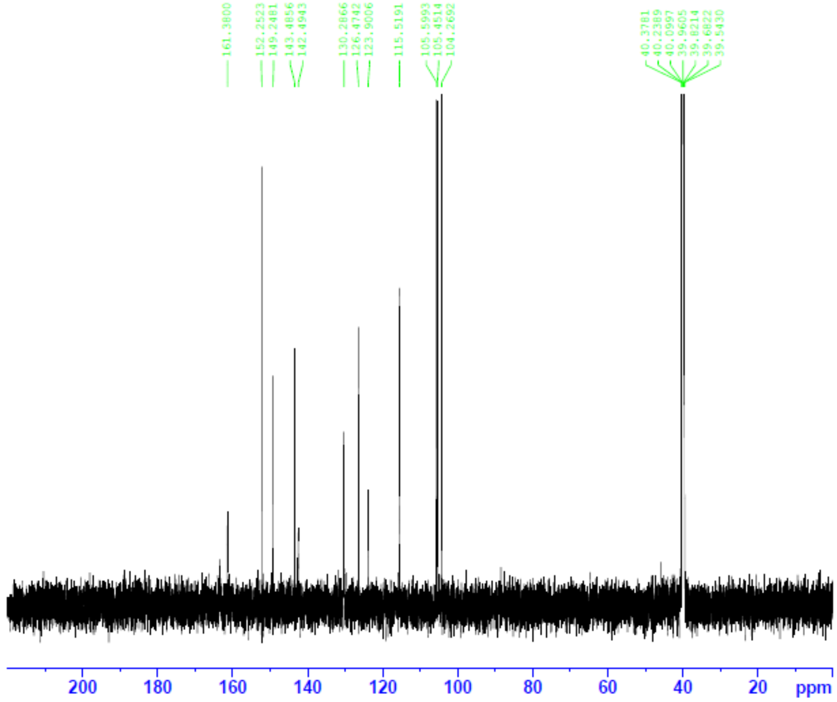


```
Current Data Parameters
NAME       Apr16-2025-Dr. Deb
EXPNO                   51
PROCNO                   1

F2 - Acquisition Parameters
Date_           20250416
Time               12.16 h
INSTRUM            spect
PROBHD   Z114261_0017 (
PULPROG          zgpg30
TD                 65536
SOLVENT             DMSO
NS                  2048
DS                     4
SWH            36231.883 Hz
FIDRES          1.105709 Hz
AQ             0.9043968 sec
RG                   203
DW                13.800 usec
DE                  6.50 usec
TE                 298.7 K
D1            2.00000000 sec
D11           0.03000000 sec
TD0                    1
SFO1         150.8852070 MHz
NUC1                 13C
P0                  3.33 usec
P1                 10.00 usec
PLW1         97.50000000 W
SFO2         600.0024000 MHz
NUC2                  1H
CPDPRG[2         waltz65
PCPD2              70.00 usec
PLW2         27.00000000 W
PLW12         0.66672999 W
PLW13         0.33535999 W

F2 - Processing parameters
SI                 32768
SF           150.8701200 MHz
WDW                   EM
SSB                    0
LB                  1.00 Hz
GB                     0
PC                  1.40
```

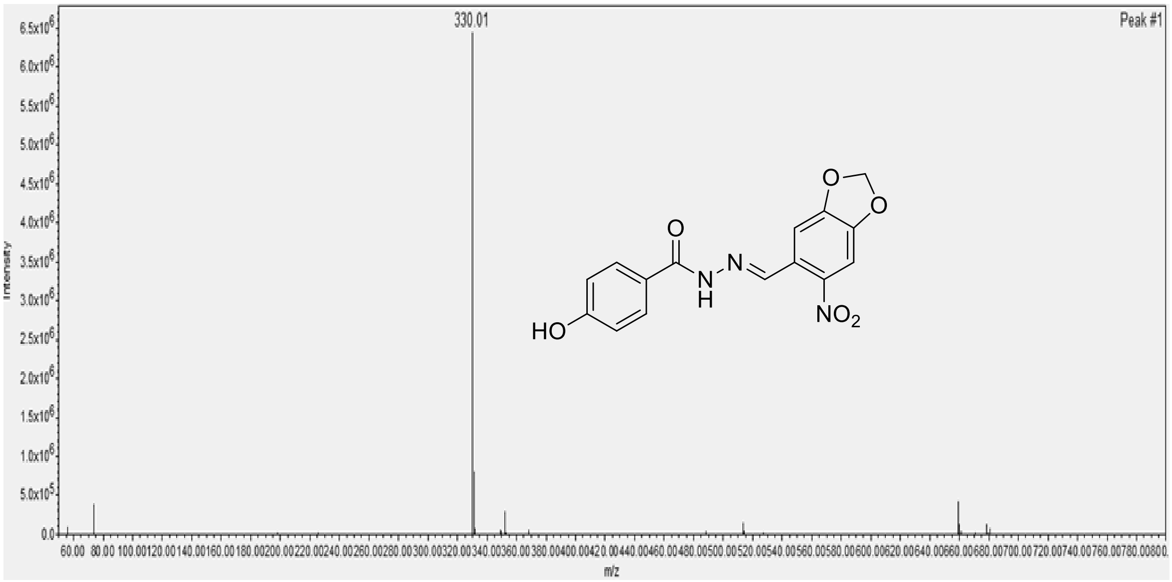

Spectrums of **Nfz-24**

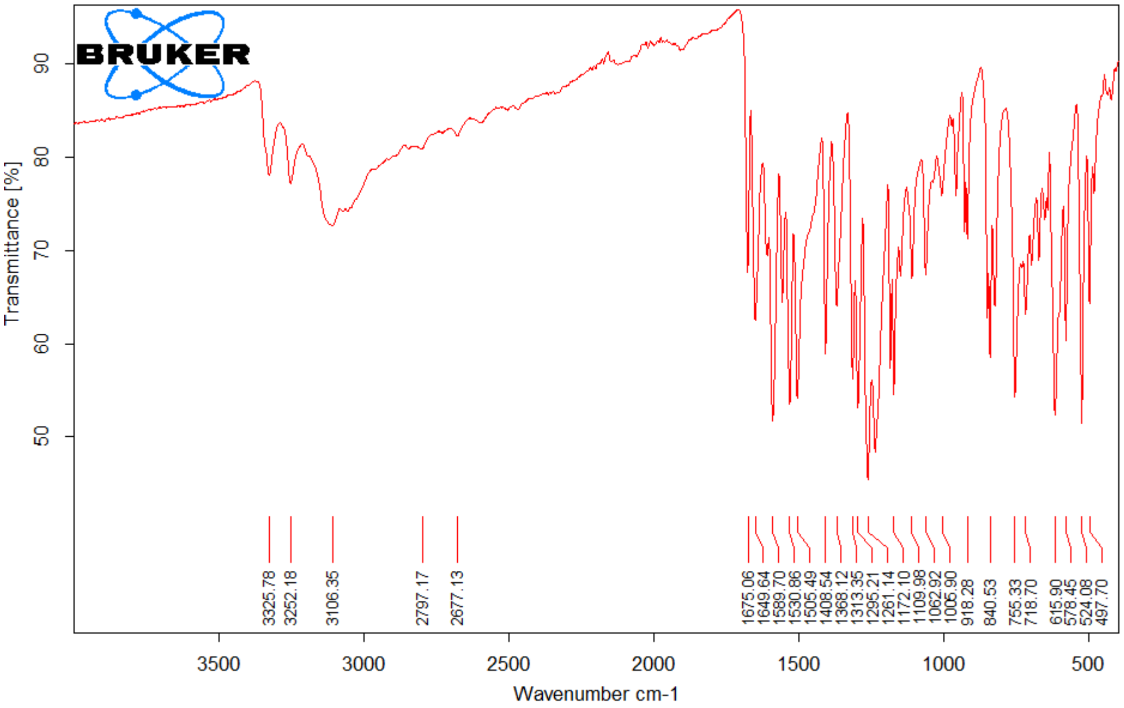

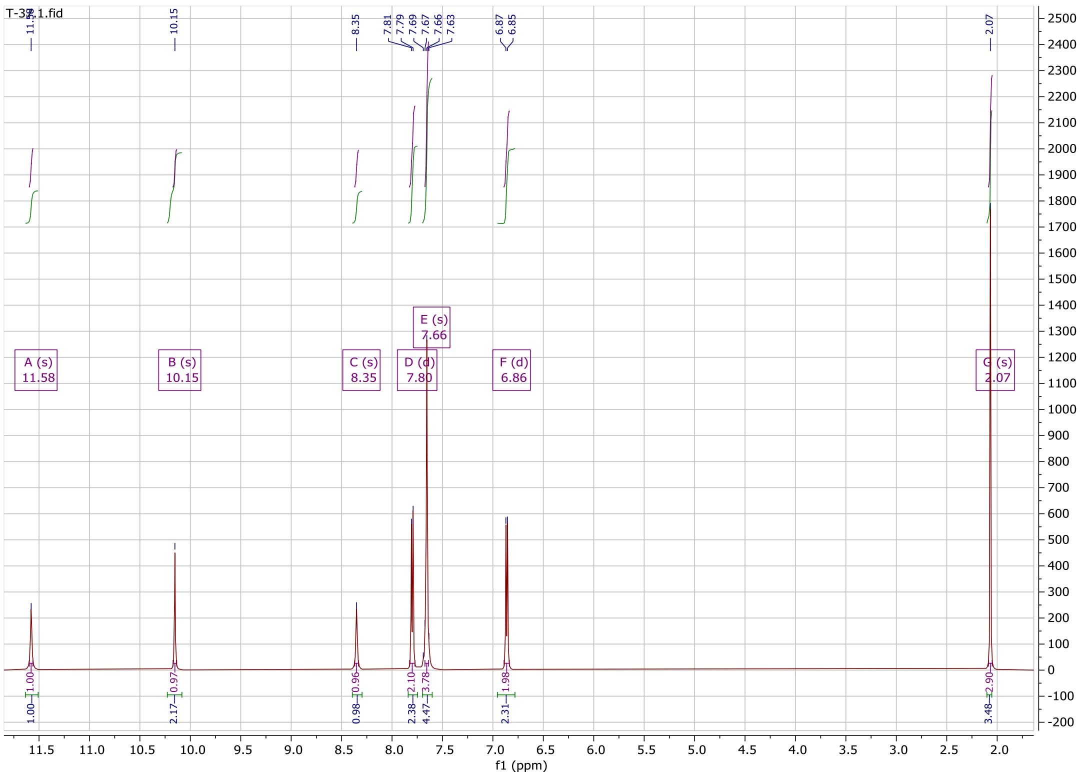

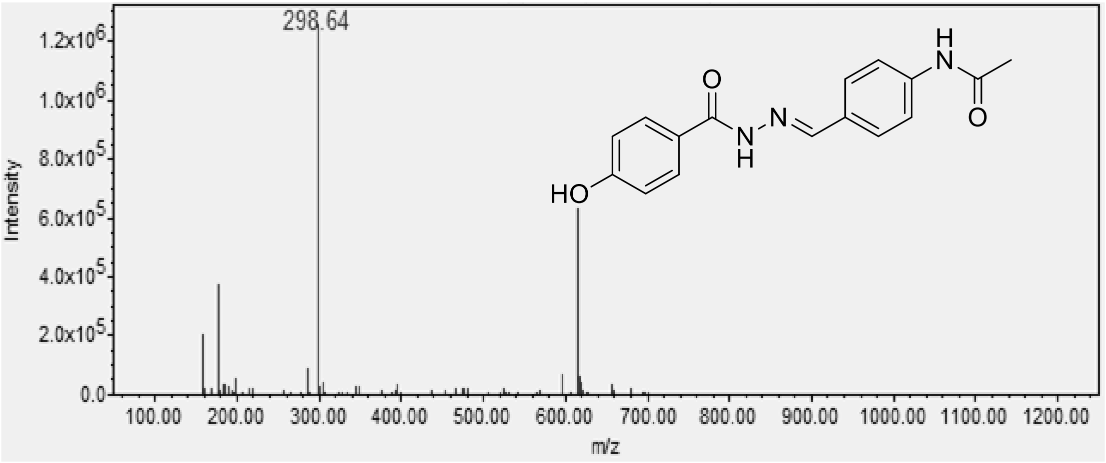

Spectrums of **Nfz-25**

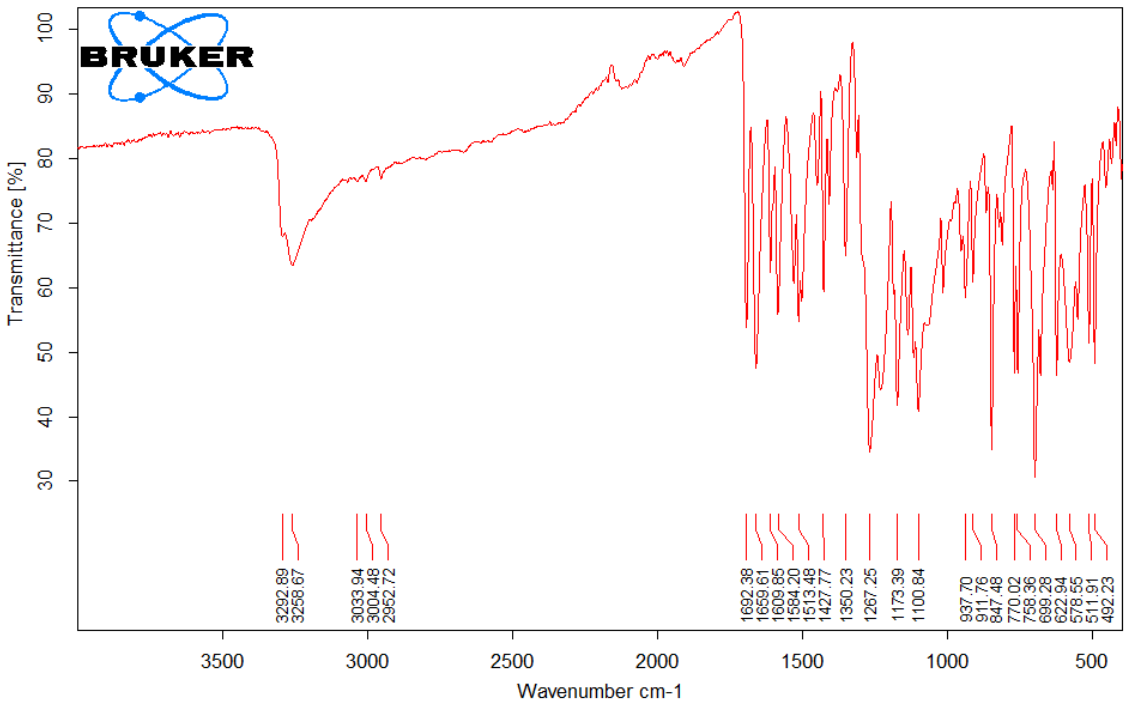

BRUKER
Transmittance [%]
Wavenumber cm-1
3292.89
3258.67
3033.94
3004.48
2952.72
1692.38
1659.61
1609.85
1584.20
1513.48
1427.77
1350.23
1267.25
1173.39
1100.84
937.70
911.76
847.48
770.02
758.36
699.28
622.94
578.55
511.91
492.23

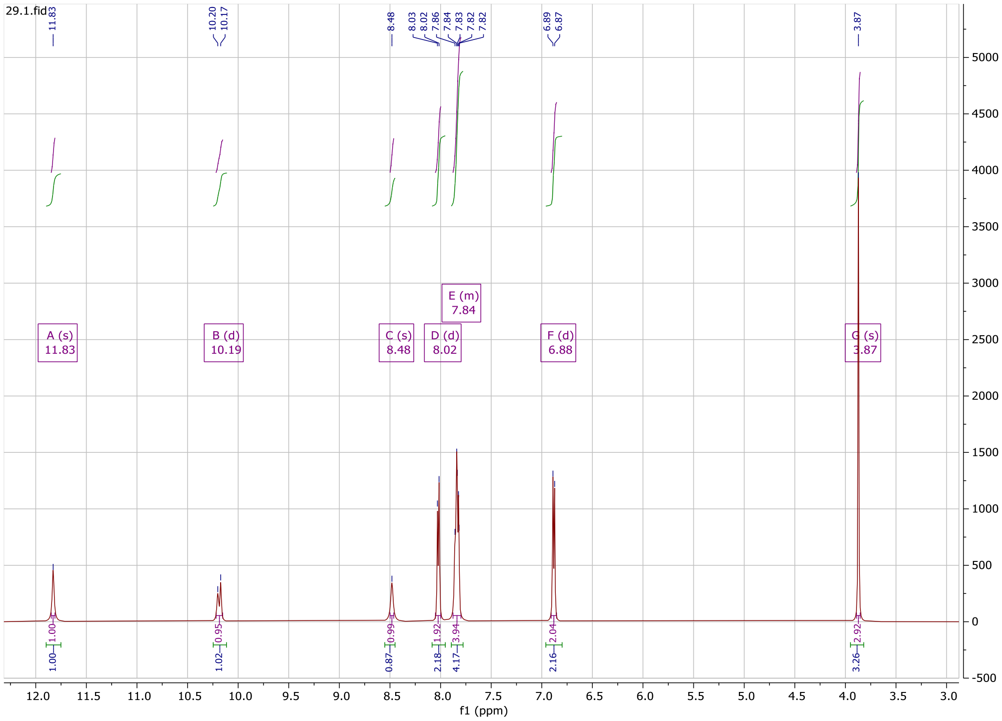

29.1.fid
A (s) 11.83
B (d) 10.19
C (s) 8.48
D (d) 8.02
E (m) 7.84
F (d) 6.88
G (s) 3.87
f1 (ppm)

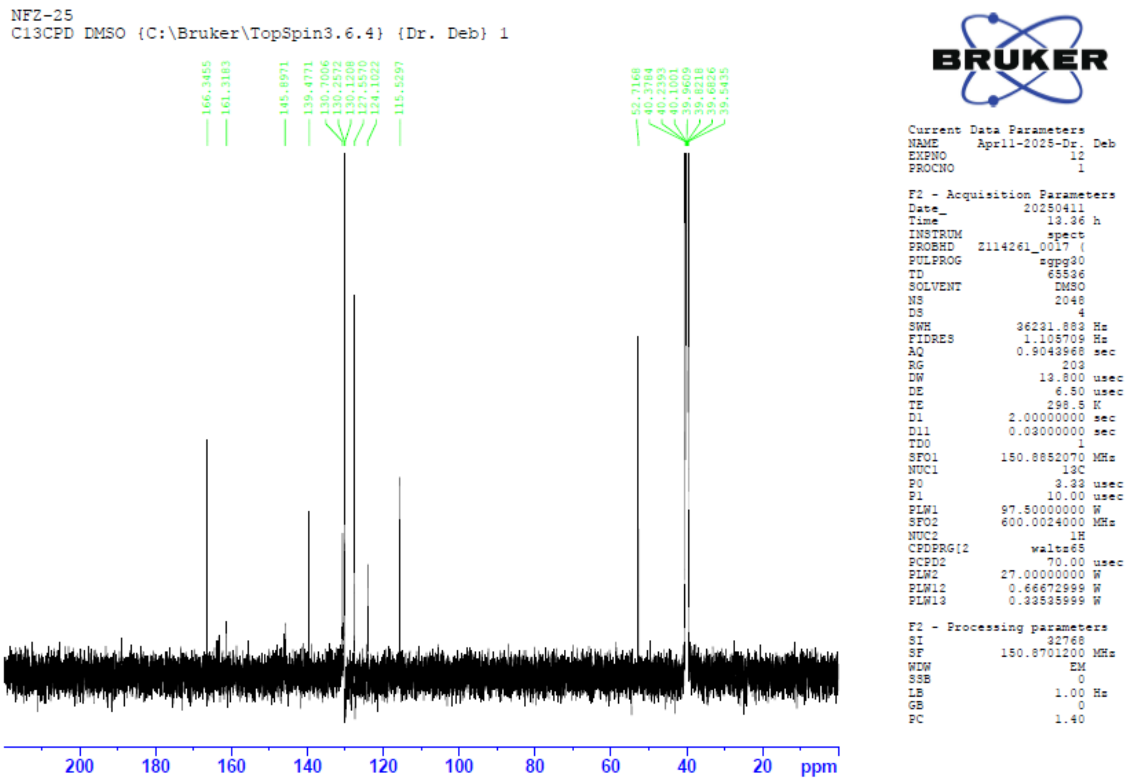
NFZ-25
C13CPD DMSO {C:\Bruker\TopSpin3.6.4} {Dr. Deb} 1
166.3455
161.3183
145.8971
139.4771
130.7006
130.2572
130.1208
127.5570
124.1022
115.5297
52.7168
40.3784
40.2393
40.1001
39.9609
39.8218
39.6826
39.5435
200
180
160
140
120
100
80
60
40
20
ppm
Current Data Parameters
NAME Apr11-2025-Dr. Deb
EXPNO 12
PROCNO 1
F2 - Acquisition Parameters
Date_ 20250411
Time 13.36 h
INSTRUM spect
PROBHD Z114261_0017 (
PULPROG zgpg30
TD 65536
SOLVENT DMSO
NS 2048
DS 4
SWH 36231.883 Hz
FIDRES 1.105709 Hz
AQ 0.9043968 sec
RG 203
DW 13.800 usec
DE 6.50 usec
TE 298.5 K
D1 2.00000000 sec
D11 0.03000000 sec
TD0 1
SFO1 150.8852070 MHz
NUC1 13C
P0 3.33 usec
P1 10.00 usec
PLW1 97.50000000 W
SFO2 600.0024000 MHz
NUC2 1H
CPDPRG[2 waltz65
PCPD2 70.00 usec
PLW2 27.00000000 W
PLW12 0.66672999 W
PLW13 0.33535999 W
F2 - Processing parameters
SI 32768
SF 150.8701200 MHz
WDW EM
SSB 0
LB 1.00 Hz
GB 0
PC 1.40

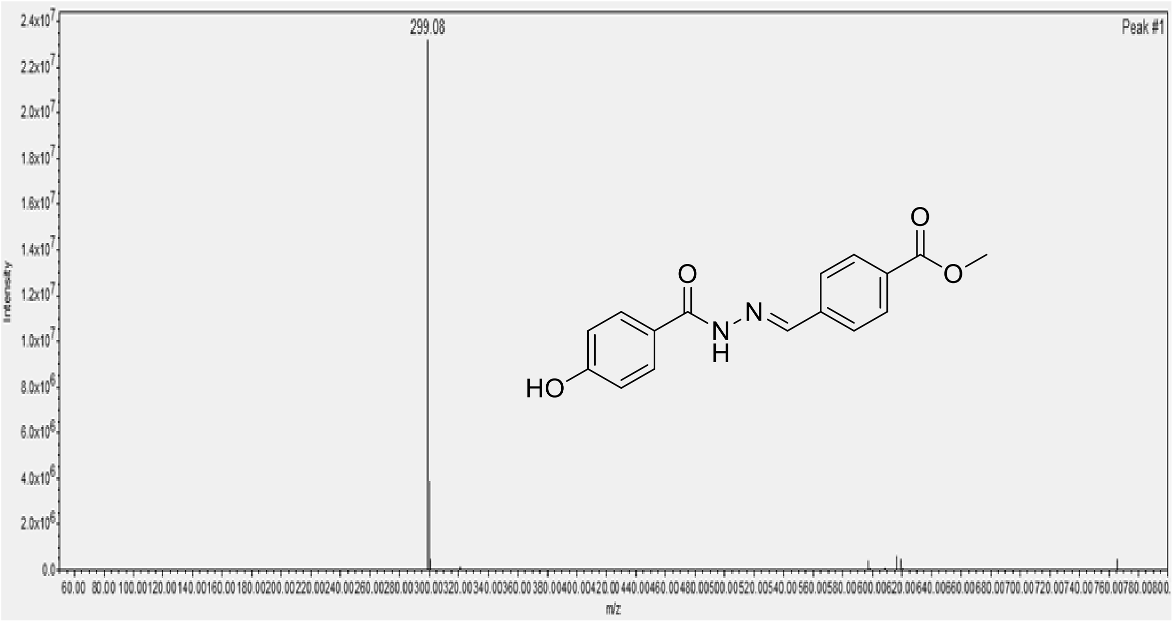
299.08
Peak #1
Intensity
m/z
HO
O
N
H
N
O
O
